# Supplementary figures and images for: Risk and prognosis of second primary malignancies in patients with follicular lymphoma in the era of rituximab: A population study based on the SEER database
Source: PLoS One. 2025 May 28;20(5):e0324532. doi: 10.1371/journal.pone.0324532 (PMC12118830; doi:10.1371/journal.pone.0324532)

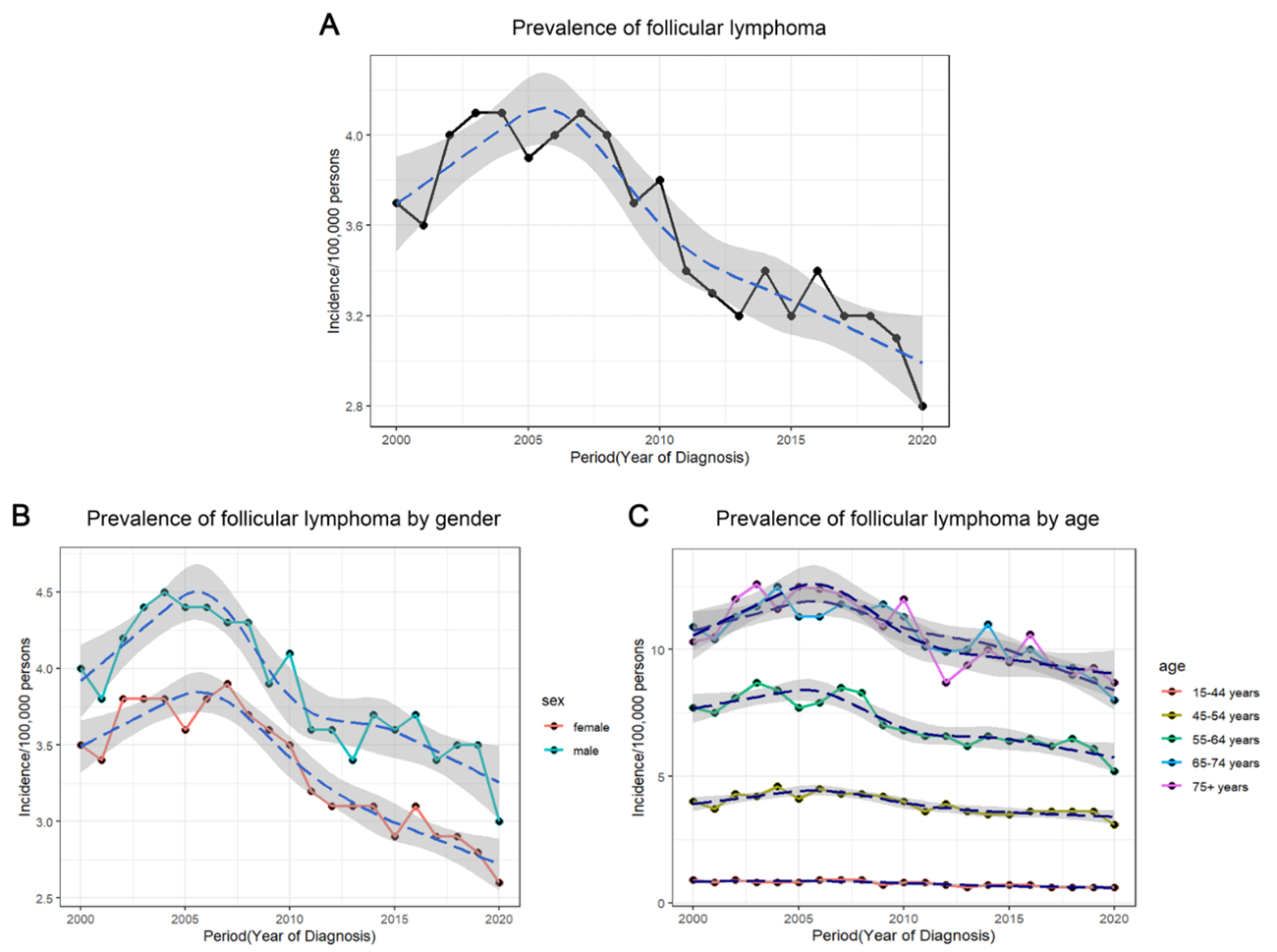

Supplement: S1 Fig — (A)Prevalence of follicular lymphoma by gender (B)Prevalence of follicular lymphoma by age (C) per 10,000: Surveillance, Epidemiology, and End Results (SEER) 2000–2020. (TIF) [file pone.0324532.s001.tif]
