# Supplementary material for: Risk and prognosis of second primary malignancies in patients with follicular lymphoma in the era of rituximab: A population study based on the SEER database
Source: PLoS One. 2025 May 28;20(5):e0324532. doi: 10.1371/journal.pone.0324532 (PMC12118830; doi:10.1371/journal.pone.0324532)
Supplement: S1 Table — (DOCX) [file pone.0324532.s002.docx]

S1 Table

| **Clinical Characteristic** | **Prevalence Rate**  **(n/100,000 person-years)** |
| --- | --- |
| **Sex** |  |
| Male | 3.8 |
| Female | 3.3 |
| **Race** |  |
| White | 4.0 |
| Black | 1.6 |
| Others^a^ | 1.8 |
| **Age** |  |
| 15-44 | 0.8 |
| 45-54 | 3.9 |
| 55-64 | 7 |
| 65-74 | 10.4 |
| 75+ | 10.5 |
| **FL–subtype** |  |
| Grade 1 | 0.8 |
| Grade 2 | 1.1 |
| Grade 3 | 0.6 |
| **Site** |  |
| NHL–Extranodal | 0.5 |
| NHL–Nodal | 3.0 |

a Others for race represented American Indians/AK native and Asian/Pacific Islanders
