# Supplementary material for: Risk and prognosis of second primary malignancies in patients with follicular lymphoma in the era of rituximab: A population study based on the SEER database
Source: PLoS One. 2025 May 28;20(5):e0324532. doi: 10.1371/journal.pone.0324532 (PMC12118830; doi:10.1371/journal.pone.0324532)
Supplement: S2 Table — (DOCX) [file pone.0324532.s003.docx]

S2 Table

| **Tratments(N,%)** | **All patients** | **non-SPMs** | **SPMs** | **P value^a^** |
| --- | --- | --- | --- | --- |
| All patients | N=33104 | 29282 (88.5%) | 3822 (11.5%) | - |
|  |  |  |  |  |
| Radiotherapy+Chemotherapy+Surgery | 1052（3.2%） | 877（3.0%） | 175（4.6%） | **<0.001** |
| Radiotherapy+Chemotherapy | 1409（4.3%） | 1209（4.1%） | 199（5.2%） |  |
| Radiotherapy+Surgery | 1831（5.5%） | 1582（5.4%） | 248（6.5%） |  |
| Chemotherapy+Surgery | 6434（19.4%） | 5574（19.0%） | 860（22.5%） |  |
| Radiotherapy only | 2073（6.3%） | 1875（6.4%） | 196（5.1%） |  |
| Chemotherapy only | 13253（40.0%） | 11914（40.7%） | 1330（34.8%） |  |
| Surgery only | 5727（17.3%） | 4982（17.0%） | 745（19.5%） |  |
| Not received | 1347（4.1%） | 1269（4.3%） | 69（1.8%） | **<0.001** |
| Multiple treatment | 10724(32.4%) | 9242 (31.6%) | 1482 (38.8%) |  |
| Single treatment | 21042 (63.6%) | 18771 (64.1%) | 2271 (59.4%) |  |

a χ2 test was used for comparison. Significant values (P<0 .05) are highlighted in bold.
