# Supplementary material for: Risk and prognosis of second primary malignancies in patients with follicular lymphoma in the era of rituximab: A population study based on the SEER database
Source: PLoS One. 2025 May 28;20(5):e0324532. doi: 10.1371/journal.pone.0324532 (PMC12118830; doi:10.1371/journal.pone.0324532)
Supplement: S3 Table — (DOCX) [file pone.0324532.s004.docx]

S3 Table

| **Primary location(N,%)** | **All patients** | **non-SPMs** | **SPMs** | **P value^a^** | **time to SPMs,m** | | |
| --- | --- | --- | --- | --- | --- | --- | --- |
|  |  |  |  |  | **Median**^b^ | **IQR** | **Range** |
| All patients | N=33104 | 29282 (88.5%) | 3822 (11.5%) | **-** |  |  |  |
| Lymph nodes of multiple regions | 13948 (42.1%) | 12409(42.4%) | 1539(40.3%) | **0.012** | 62 | 29-105 | 6-245 |
| Lymph node, NOS | 3697 (11.2%) | 3303(11.3%) | 394(10.3%) | 0.078 | 53 | 22.75-97.25 | 6-240 |
| Lymph nodes of head, face & neck | 3580 (10.8%) | 3078(10.5%) | 502(13.1%) | **<0.001** | 61 | 31.75-109 | 6-240 |
| Intra-abdominal lymph nodes | 2832 (8.6%) | 2556(8.7%) | 276(7.2%) | **0.002** | 63 | 31-101 | 6-224 |
| Lymph nodes of inguinal region or leg | 2488 (7.5%) | 2183(7.5%) | 305(8.0%) | 0.289 | 59 | 31-109.5 | 6-234 |
| Lymph nodes of axilla or arm | 1015 (3.1%) | 894(3.1%) | 121(3.2%) | 0.683 | 79 | 35-116 | 6-223 |
| Skin | 754 (2.3%) | 646(2.2%) | 108(2.8%) | **0.018** | 56 | 30.5-99.75 | 6-208 |
| other | 637 (1.9%) | 554(1.9%) | 83(2.2%) | 0.214 | 73 | 35-127 | 6-227 |
| Parotid gland | 458 (1.4%) | 397(1.4%) | 61(1.6%) | 0.249 | 67 | 36-116.5 | 8-237 |
| Tonsil | 330 (1.0%) | 289(1.0%) | 41(1.1%) | 0.536 | 70 | 29-115.5 | 6-208 |
| Intrathoracic lymph nodes | 319 (1.0%) | 288(1.0%) | 31(0.8%) | 0.290 | 46 | 25-100 | 7-143 |
| Small intestine, NOS | 286 (0.9%) | 253(0.9%) | 33(0.9%) | 0.972 | 69 | 42.5-141.5 | 10-217 |
| soft tissue | 281 (0.8%) | 241(0.8%) | 40(1.0%) | 0.167 | 68 | 39.75-115.75 | 6-239 |
| duodenum | 263 (0.8%) | 237(0.8%) | 26(0.7%) | 0.483 | 74.5 | 38.75-123.5 | 13-213 |
| Pelvic lymph nodes | 245 (0.7%) | 215(0.7%) | 30(0.8%) | 0.753 | 37.5 | 21-74.25 | 6-208 |
| colon | 214 (0.6%) | 191(0.7%) | 23(0.6%) | 0.695 | 61 | 18-111 | 8-140 |
| breast | 202 (0.6%) | 180(0.6%) | 22(0.6%) | 0.903 | 55.5 | 13.75-134.5 | 6-173 |
| ileum | 144 (0.4%) | 124(0.4%) | 20(0.5%) | 0.391 | 47.5 | 27.25-126.25 | 12-176 |
| Orbit, NOS | 139 (0.4%) | 124(0.4%) | 15(0.4%) | 0.948 | 55 | 8-90 | 8-174 |
| Spleen | 125 (0.4%) | 111(0.4%) | 14(0.4%) | 0.888 | 52 | 29-84.25 | 9-108 |
| Jejunum | 115 (0.4%) | 101(0.3%) | 14(0.4%) | 0.483 | 45 | 23-92.25 | 7-201 |
| Thyroid gland | 112 (0.3%) | 102(0.3%) | 10(0.3%) | 0.376 | 79.5 | 38.75-129.5 | 22-218 |
| stomach | 105 (0.3%) | 90(0.3%) | 15(0.4%) | 0.390 | 36 | 26-65 | 9-139 |
| Submandibular gland | 94 (0.3%) | 82(0.3%) | 12(0.3%) | 0.725 | 52.5 | 35-131 | 14-168 |
| Lung | 85 (0.3%) | 74(0.3%) | 11(0.3%) | 0.496 | 54 | 24-106 | 10-143 |
| Vertebral column | 83 (0.3%) | 73(0.2%) | 10(0.3%) | 0.899 | 38.5 | 22.5-139.25 | 17-168 |
| peritoneum | 78 (0.2%) | 69(0.2%) | 9(0.2%) | 0.986 | 79 | 53.5-121.5 | 14-135 |
| pancreas | 75 (0.2%) | 66(0.2%) | 9(0.2%) | 0.915 | 31 | 15.5-85 | 8-95 |
| conjunctiva | 68 (0.2%) | 57(0.2%) | 11(0.3%) | 0.239 | 76 | 42-152 | 29-204 |
| Retroperitoneum | 65 (0.2%) | 58(0.2%) | 7(0.2%) | 0.833 | 50 | 19-97 | 18-175 |
| female genital tract | 57 (0.2%) | 53(0.2%) | 4(0.1%) | 0.279 | 82.5 | 49.25-109 | 46-110 |
| tongue | 56 (0.2%) | 50(0.2%) | 6(0.2%) | 0.835 | 57.5 | 26.75-171 | 23-192 |
| Nasopharynx | 55 (0.2%) | 48(0.2%) | 7(0.2%) | 0.795 | 53 | 48-119 | 24-180 |
| Kidney, NOS | 51 (0.2%) | 44(0.2%) | 7(0.2%) | 0.394 | 69 | 23-78 | 10-143 |
| Palate | 48 (0.1%) | 42(0.1%) | 6(0.2%) | 0.846 | 33 | 21.5-52.5 | 20-57 |

IQR: interquartile range

a χ2 test was used for comparison. Significant values (P <0 .05) are highlighted in bold.

b Kruskall-Wallis test used for calculations and comparisons.
