# Supplementary material for: Risk and prognosis of second primary malignancies in patients with follicular lymphoma in the era of rituximab: A population study based on the SEER database
Source: PLoS One. 2025 May 28;20(5):e0324532. doi: 10.1371/journal.pone.0324532 (PMC12118830; doi:10.1371/journal.pone.0324532)
Supplement: S4 Table — (DOCX) [file pone.0324532.s005.docx]

S4 Table

| **Site** | **Number(N=3822)** |
| --- | --- |
| Lung | 603 (15.78%) |
| Prostate | 482 (12.61%) |
| Gastrointestinal Tract | 419 (10.96%) |
| Breast | 332 (8.69%) |
| Skin/soft tissue | 272 (7.12%) |
| Miscellaneous | 242 (6.33%) |
| Urinary Bladder | 202 (5.29%) |
| Female genital system | 160 (4.19%) |
| Kidney and Renal Pelvis | 132 (3.45%) |
| Head and Neck | 120 (3.14%) |
| Pancreas | 108 (2.83%) |
| Thyroid | 101 (2.64%) |
| Liver | 53 (1.39%) |
| Biliary Tract | 37 (0.97%) |
| Brain | 32 (0.84%) |
| Others^a^ | 79 (2.07%) |
| Hematologic Malignancies |  |
| Myeloid/Monocytic Leukemia | 175 (4.58%) |
| Non-Hodgkin lymphoma | 145 (3.79%) |
| Hodgkin lymphoma | 50 (1.31%) |
| Myeloma | 40 (1.05%) |
| Lymphocytic Leukemia | 33 (0.86%) |
| Other Acute Leukemia | 5 (0.13%) |

a Others included salivary gland, mesothelioma, Kaposi sarcoma, bones and joints, appendix, aleukemic, subleukemic, male genital organs, other urinary organs, other digestive organs, retroperitoneum, peritoneum, omentum, mesentery, and other endocrine including thymus.
