# Supplementary material for: Risk and prognosis of second primary malignancies in patients with follicular lymphoma in the era of rituximab: A population study based on the SEER database
Source: PLoS One. 2025 May 28;20(5):e0324532. doi: 10.1371/journal.pone.0324532 (PMC12118830; doi:10.1371/journal.pone.0324532)
Supplement: S5 Table — (DOCX) [file pone.0324532.s006.docx]

S5 Table

| **characteristic** | **All patients** | **All patients** | **P-value^a^** |
| --- | --- | --- | --- |
|  | **(without<6m)** | **(with<6m)** |  |
|  | N=33104 | N=33610 |  |
| **Sex** |  |  |  |
| Male | 16683 (50.4%) | 16950(50.4%) | 0.932 |
| Female | 16421 (49.6%) | 16660(49.4%) |  |
| **Age at diagnosis** |  |  |  |
| 15-39 | 1955 (5.9%) | 1964(5.8%) | 0.744 |
| 40-60 | 13423 (40.5%) | 13551(40.3%) |  |
| >60 | 17726 (53.6%) | 18095(53.8%) |  |
| **Race** |  |  |  |
| White | 29671 (89.6%) | 30145(89.7%) | 0.967 |
| Black | 1551(4.7%) | 1564(4.7%) |  |
| Others^b^ | 1882 (5.7%) | 1901(5.7%) |  |
| **Ethnicity** |  |  |  |
| Hispanics | 4161 (12.6%) | 4205(12.5%) | 0.820 |
| Non-Hispanics | 28943 (87.4%) | 29405(87.5%) |  |
| **FL-subtype** |  |  |  |
| Grade1-2 | 17409 (52.6%) | 17673(52.6%) | 0.999 |
| Grade3 | 6450 (19.5%) | 6545(19.5%) |  |
| Grade NOS | 9245 (27.9%) | 9392(27.9%) |  |
| **Ann Arbor stage** |  |  |  |
| I/ II | 10925 (33.0%) | 11101(33.0%) | 0.993 |
| III/IV | 13750 (41.5%) | 13945(41.5%) |  |
| Unknown | 8429 (25.5%) | 8564(25.5%) |  |
| **B symptom** |  |  |  |
| None | 10836 (32.7%) | 11026(32.8%) | 0.978 |
| Any | 3176 (9.6%) | 3226(9.6%) |  |
| Unknown | 19092 (57.7%) | 19358(57.6%) |  |
| **Radiotherapy** | 6361 (19.2%) | 6451(19.2%) | 0.944 |
| **Chemotherapy** | 22138 (66.9%) | 22454(66.8%) | 0.855 |
| **Surgery** | 15031 (45.4%) | 15270(45.4%) | 0.943 |
| **Diagnosis-to-treatment-time** |  |  |  |
| ≤1month | 25345 (76.6%) | 25716(76.5%) | 0.882 |
| >1month | 7759 (23.4%) | 7894(23.5%) |  |
| **Marital status** |  |  |  |
| Married | 21618(65.3%) | 21951(65.3%) | 0.999 |
| Single | 4461(13.5%) | 4525(13.5%) |  |
| Others^c^ | 7025(21.2%) | 7134(21.2%) |  |
| **Income** |  |  |  |
| <$65,000 | 10430 (31.5%) | 10591(31.5%) | 0.996 |
| $65,000 - $74,999 | 8492 (25.6%) | 8612(25.6%) |  |
| ≥$75,000 | 14182 (42.8%) | 14407(42.9%) |  |
| **Rural-Ubran** |  |  |  |
| Metropolitan areas | 28636 (86.5%) | 29074(86.5%) | 0.997 |
| Nonmetropolitan counties | 4468 (13.5%) | 4536(13.5%) |  |
| **Site** |  |  |  |
| NHL – Extranodal | 4527 (13.7%) | 4611(13.7%) | 0.869 |
| NHL – Nodal | 28577 (86.3%) | 28999(86.3%) |  |
| **Year of diagnosis** |  |  |  |
| 2000-2004 | 8299 (25.1%) | 8397(25%) | 0.998 |
| 2005-2009 | 8727 (26.4%) | 8864(26.4%) |  |
| 2010-2014 | 7372 (22.3%) | 7484(22.3%) |  |
| 2015-2019 | 7491 (22.6%) | 7636(22.7%) |  |
| 2020 | 1215 (3.7%) | 1229(3.7%) |  |
| **spm** | 3822(11.5%) | 4328(12.9%) | **<0.0001** |

a χ2 test was used for comparison. Significant values (P <0 .05) are highlighted in bold.

b Others for race represented American Indian/AK Native, Asian/Pacific Islander.

c Others for marital status represented divorced, separated, unmarried or domestic partner, widowed.
