# Supplementary material for: Risk and prognosis of second primary malignancies in patients with follicular lymphoma in the era of rituximab: A population study based on the SEER database
Source: PLoS One. 2025 May 28;20(5):e0324532. doi: 10.1371/journal.pone.0324532 (PMC12118830; doi:10.1371/journal.pone.0324532)
Supplement: S6 Table — (DOCX) [file pone.0324532.s007.docx]

S6 Table

| **characteristic** | **CP-HR^a^**  **(N=33104)** | **P-value** | **CP-HR^b^**  **(N=33610)** | **P-value** | **C-HR^c^**  **(N=33104)** | **P-value** | **C-HR^d^**  **(N=33610)** | **P-value** |
| --- | --- | --- | --- | --- | --- | --- | --- | --- |
| **Sex** |  |  |  |  |  |  |  |  |
| Male | 1 |  | 1 |  | 1 |  | 1 |  |
| Female | 0.80(0.75-0.85) | **<0.001** | 0.81(0.77-0.86) | **<0.001** | 0.78(0.73-0.83) | **<0.001** | 0.79(0.75-0.84) | **<0.001** |
| **Age at diagnosis** |  |  |  |  |  |  |  |  |
| 15-39 | 1 |  | 1 |  | 1 |  | 1 |  |
| 40-60 | 2.66(2.14-3.31) | **<0.001** | 2.59(2.10-3.19) | **<0.001** | 2.86(2.29-3.56) | **<0.001** | 2.78(2.25-3.43) | **<0.001** |
| >60 | 3.52(2.83-4.37) | **<0.001** | 3.60(2.93-4.42) | **<0.001** | 5.27(4.23-6.56) | **<0.001** | 5.21(4.23-6.41) | **<0.001** |
| **Race** |  |  |  |  |  |  |  |  |
| White | 1 |  | 1 |  | 1 |  | 1 |  |
| Black | 0.88(0.76-1.03) | 0.12 | 0.84(0.72-0.98) | 0.023 | 0.91(0.78-1.07) | 0.259 | 0.87(0.74-1.00) | 0.06 |
| Others^e^ | 0.80(0.68-0.93) | **0.004** | 0.77(0.66-0.89) | **<0.001** | 0.77(0.66-0.91) | **0.001** | 0.76(0.65-0.88) | **<0.001** |
| **Ethnicity** |  |  |  |  |  |  |  |  |
| Hispanics | 1 |  | 1 |  | 1 |  | 1 |  |
| Non-Hispanics | 1.41(1.26-1.58) | **<0.001** | 1.41(1.27-1.57) | **<0.001** | 1.42(1.26-1.59) | **<0.001** | 1.43(1.28-1.59) | **<0.001** |
| **FL-subtype** |  |  |  |  |  |  |  |  |
| Grade1-2 | 1 |  | 1 |  | 1 |  | 1 |  |
| Grade3 | 0.89(0.81-0.97) | **0.007** | 0.90(0.83-0.97) | **0.008** | 0.93(0.85-1.01) | 0.082 | 0.93(0.86-1.01) | 0.088 |
| Grade NOS | 0.94(0.87-1.01) | 0.10 | 0.95(0.89-1.02) | 0.15 | 1.01(0.94-1.09) | 0.766 | 1.02(0.95-1.09) | 0.658 |
| **Ann Arbor stage** |  |  |  |  |  |  |  |  |
| I/ II | 1 |  | 1 |  | 1 |  | 1 |  |
| III/IV | 0.94(0.87,1.00) | 0.051 | 0.93(0.87-0.99) | **0.027** | 1.04(0.97-1.11) | 0.32 | 1.02(0.96-1.09) | 0.562 |
| Unknown | 0.89(0.80-0.99) | **0.029** | 0.92(0.83-1.01) | 0.082 | 1.06(0.95-1.19) | 0.272 | 1.06(0.96,1.17) | 0.230 |
| **B symptom** |  |  |  |  |  |  |  |  |
| None | 1 |  | 1 |  | 1 |  | 1 |  |
| Any | 0.93(0.79-1.08) | 0.32 | 0.92(0.80-1.06) | 0.25 | 1.05(0.91-1.23) | 0.502 | 1.02(0.89-1.17) | 0.748 |
| Unknown | 1.13(1.04-1.23) | **0.003** | 1.07(0.99-1.15) | 0.091 | 1.04(0.96-1.13) | 0.373 | 0.99(0.92-1.07) | 0.865 |
| **Radiotherapy** | 1.13(1.04-1.22) | **0.002** | 0.91(0.84-0.98) | **0.009** | 0.98(0.90-1.06) | 0.549 | 0.99(0.92-1.07) | 0.775 |
| **Chemotherapy** | 0.99(0.92-1.05) | 0.68 | 1.03(0.97-1.10) | 0.34 | 0.96(0.89-1.02) | 0.179 | 0.98(0.92-1.05) | 0.584 |
| **Surgery** | 1.06(0.99-1.13) | 0.081 | 0.95(0.89-1.00) | 0.068 | 1.04(0.98-1.11) | 0.197 | 1.03(0.97-1.09) | 0.342 |
| **Diagnosis-to-treatment-time** |  |  |  |  |  |  |  |  |
| ≤1month | 1 |  | 1 |  | 1 |  | 1 |  |
| >1month | 0.97(0.89-1.05) | 0.44 | 1.00(0.93-1.08) | 0.99 | 0.96(0.89-1.04) | 0.342 | 0.99(0.92-1.07) | 0.78 |
| **Marital status** |  |  |  |  |  |  |  |  |
| Married | 1 |  | 1 |  | 1 |  | 1 |  |
| Single | 0.76(0.68-0.84) | **<0.001** | 0.78(0.71-0.86) | **<0.001** | 0.77(0.70-0.86) | **<0.001** | 0.79(0.72-0.87) | **<0.001** |
| other^f^ | 0.91(0.84-0.98) | **0.018** | 0.92(0.85-0.99) | **0.021** | 1.12(1.04-1.22) | **0.004** | 1.11(1.03-1.20) | **0.007** |
| **Income** |  |  |  |  |  |  |  |  |
| <$65,000 | 1 |  | 1 |  | 1 |  | 1 |  |
| $65,000 - $74,999 | 0.89(0.82-0.97) | **0.007** | 0.90(0.83-0.97) | **0.008** | 0.86(0.79-0.94) | **<0.001** | 0.87(0.80-0.94) | **<0.001** |
| ≥$75,000 | 0.94(0.87-1.01) | 0.086 | 0.95(0.89-1.02) | 0.17 | 0.86(0.80-0.93) | **<0.001** | 0.88(0.82-0.95) | **<0.001** |
| **Rural-Ubran** |  |  |  |  |  |  |  |  |
| Metropolitan areas | 1 |  | 1 |  | 1 |  | 1 |  |
| Nonmetropolitan counties | 1.10(1.01-1.21) | **0.027** | 1.09(1.00-1.18) | **0.047** | 1.17(1.07-1.28) | **<0.001** | 1.15(1.05-1.25) | **0.001** |
| **Site** |  |  |  |  |  |  |  |  |
| NHL – Extranodal | 1 |  | 1 |  | 1 |  | 1 |  |
| NHL – Nodal | 0.90(0.82-0.98) | **0.019** | 0.89(0.82-0.97) | **0.007** | 0.96(0.87-1.04) | 0.314 | 0.94(0.86-1.02) | 0.114 |
| **Year of diagnosis** |  |  |  |  |  |  |  |  |
| 2000-2004 | 1 |  | 1 |  | 1 |  | 1 |  |
| 2005-2009 | 0.94(0.88-1.02) | 0.13 | 0.97(0.90-1.04) | 0.41 | 0.98(0.91-1.06) | 0.567 | 1.00(0.93-1.08) | 0.936 |
| 2010-2014 | 0.88(0.81-0.96) | **0.004** | 0.91(0.84-0.99) | **0.024** | 0.97(0.88-1.07) | 0.525 | 1.00(0.91-1.09) | 0.945 |
| 2015-2019 | 0.76(0.68-0.86) | **<0.001** | 0.89(0.80-0.99) | **0.030** | 0.97(0.86-1.11) | 0.687 | 1.07(0.96-1.20) | 0.201 |
| 2020 | 0.75(0.35-1.60) | 0.45 | 0.80(0.52-1.24) | 0.32 | 1.98(0.93-4.20) | 0.077 | 1.04(0.67-1.62) | 0.849 |

a Univariate analysis of SPM occurrence using competing risk model analysis (excluding patients with SPMs occurring within less than 6 months from diagnosis). Significant values (P <0.05) are highlighted in bold.

b Univariate analysis of SPM occurrence using competing risk model analysis (including patients with SPMs occurring within less than 6 months from diagnosis). Significant values (P <0.05) are highlighted in bold.

c Univariate analysis of SPM occurrence using Cox proportional hazards model (excluding patients with SPMs occurring within less than 6 months from diagnosis). Significant values (P <0.05) are highlighted in bold.

d Univariate analysis of SPM occurrence using Cox proportional hazards model (including patients with SPMs occurring within less than 6 months from diagnosis). Significant values (P <0.05) are highlighted in bold.

e Others for race represented American Indian/AK Native, Asian/Pacific Islander.

f Others for marital status represented divorced, separated, unmarried or domestic partner, widowed.
