# Supplementary material for: Risk and prognosis of second primary malignancies in patients with follicular lymphoma in the era of rituximab: A population study based on the SEER database
Source: PLoS One. 2025 May 28;20(5):e0324532. doi: 10.1371/journal.pone.0324532 (PMC12118830; doi:10.1371/journal.pone.0324532)
Supplement: S7 Table — (DOCX) [file pone.0324532.s008.docx]

S7 Table

| **characteristic** | **CP-HR^a^**  **(N=33104)** | **P-value** | **CP-HR^b^**  **(N=33610)** | **P-value** | **C-HR^c^**  **(N=33104)** | **P-value** | **C-HR^d^**  **(N=33610)** | **P-value** |
| --- | --- | --- | --- | --- | --- | --- | --- | --- |
| **Sex** |  |  |  |  |  |  |  |  |
| Male | 1 |  | 1 |  | 1 |  | 1 |  |
| Female | 0.79(0.74-0.84) | **<0.001** | 0.80(0.75-0.85) | **<0.001** | 0.72(0.68-0.77) | **<0.001** | 0.74(0.70-0.79) | **<0.001** |
| **Age at diagnosis** |  |  |  |  |  |  |  |  |
| 15-39 | 1 |  | 1 |  | 1 |  | 1 |  |
| 40-60 | 2.60(2.08-3.24) | **<0.001** | 2.54(2.06-3.14) | **<0.001** | 2.81 (2.25-3.51) | **<0.001** | 2.74(2.22-3.38) | **<0.001** |
| >60 | 3.51(2.82-4.38) | **<0.001** | 3.60(2.92-4.44) | **<0.001** | 5.25(4.21-6.55) | **<0.001** | 5.15(4.17-6.35) | **<0.001** |
| **Race** |  |  |  |  |  |  |  |  |
| White | 1 |  | 1 |  | 1 |  | 1 |  |
| Black | 0.95(0.81-1.11) | 0.49 | 0.90(0.77-1.05) | 0.16 | 0.99(0.84-1.16) | 0.899 | 0.93(0.80-1.08) | 0.331 |
| Others^e^ | 0.79(0.68-0.93) | **0.004** | 0.77(0.66-0.90) | **<0.001** | 0.78(0.67-0.92) | **0.003** | 0.77(0.663-0.895) | **<0.001** |
| **Ethnicity** |  |  |  |  |  |  |  |  |
| Hispanics | 1 |  | 1 |  | 1 |  | 1 |  |
| Non-Hispanics | 1.28(1.14-1.44) | **<0.001** | 1.30(1.16-1.45) | **<0.001** | 1.27(1.13-1.43) | **<0.001** | 1.28(1.15-1.43) | **<0.001** |
| **FL-subtype** |  |  |  |  |  |  |  |  |
| Grade1-2 | 1 |  | 1 |  | 1 |  | 1 |  |
| Grade3 | 0.89(0.82-0.98) | **0.012** | 0.90(0.83-0.98) | **0.014** | 0.91(0.83-0.99) | **0.03** | 0.93(0.86-1.01) | 0.077 |
| Grade NOS | 0.92(0.86-0.99) | **0.031** | 0.93(0.87-1.00) | 0.054 | 0.99(0.92-1.06) | 0.77 | 0.99(0.93-1.07) | 0.865 |
| **Ann Arbor stage** |  |  |  |  |  |  |  |  |
| I/ II | 1 |  | 1 |  | 1 |  | - |  |
| III/IV | 1.00(0.93-1.07) | 0.94 | 0.99(0.92-1.06) | 0.76 | 1.05(0.98-1.13) | 0.17 | - |  |
| Unknown | 1.04(0.91-1.19) | 0.58 | 1.00(0.88-1.13) | 0.96 | 1.09(0.95-1.24) | 0.20 | - |  |
| **Radiotherapy** | 0.90(0.82-0.97) | **0.009** | 0.92(0.85-1.00) | **0.04** | - |  |  |  |
| **Chemotherapy** | - |  | - |  | 0.91(0.85-0.99) | **0.02** | - |  |
| **Surgery** | 0.97(0.91-1.04) | 0.40 | 0.96(0.90-1.02) | 0.16 | 0.99(0.93-1.07) | 0.86 | - |  |
| **Marital status** |  |  |  |  |  |  |  |  |
| Married | 1 |  | 1 |  | 1 |  | - |  |
| Single | 0.90(0.81-1.00) | **0.049** | 0.93(0.84-1.02) | 0.14 | 0.97(0.87-1.08) | 0.57 | 1.00(0.90-1.10) | 0.967 |
| Others^f^ | 0.90(0.83-0.97) | **0.009** | 0.90(0.83-0.97) | **0.006** | 1.09(1.00-1.18) | **0.04** | 1.07(0.99-1.15) | 0.111 |
| **Income** |  |  |  |  |  |  |  |  |
| <$65,000 | 1 |  | 1 |  | - | - | 1 |  |
| $65,000 - $74,999 | 0.93(0.84-1.02) | 0.10 | 0.93(0.85-1.01) | 0.10 | - | - | 0.92(0.85-1.01) | 0.069 |
| ≥$75,000 | 0.95(0.87-1.04) | 0.25 | 0.96(0.89-1.05) | 0.38 | - | - | 0.93(0.86-1.00) | 0.052 |
| **Rural-Ubran** |  |  |  |  |  |  |  |  |
| Metropolitan areas | 1 |  | 1 |  | 1 |  | 1 |  |
| Nonmetropolitan areas | 1.00(0.90-1.11) | 0.96 | 0.99(0.90-1.09) | 0.89 | 1.08(0.99-1.18) | 0.09 | 1.01(0.92-1.11) | 0.841 |
| **Site** |  |  |  |  |  |  |  |  |
| NHL– Extranodal | 1 |  | 1 |  | - |  | 1 |  |
| NHL– Nodal | 0.95(0.86-1.04) | 0.26 | 0.93(0.86-1.02) | 0.12 | - |  | 0.99(0.91-1.07) | 0.734 |
| **Year of diagnosis** |  |  |  |  |  |  |  |  |
| 2000-2004 | 1 |  | 1 |  | 1 |  | - |  |
| 2005-2009 | 0.94(0.87-1.02) | 0.12 | 0.97(0.90-1.04) | 0.38 | 0.96(0.89-1.04) | 0.28 | - | - |
| 2010-2014 | 0.88(0.74-1.04) | 0.13 | 0.89(0.76-1.04) | 0.14 | 0.94(0.85-1.03) | 0.17 | - | - |
| 2015-2019 | 0.75(0.60-0.93) | **0.011** | 0.87(0.71-1.06) | 0.16 | 0.90(0.77-1.05) | 0.18 | - | - |
| 2020 | 0.75(0.34-1.65) | 0.48 | 0.77(0.47-1.23) | 0.27 | 1.79(0.83-3.83) | 0.14 | - | - |
| **B symptom** |  |  |  |  |  |  |  |  |
| None | 1 |  | 1 |  |  |  |  |  |
| Any | 0.98(0.84-1.14) | 0.76 | 0.97(0.84-1.12) | 0.68 | - | - | - | - |
| Unknown | 1.01(0.85-1.21) | 0.89 | 0.98(0.84-1.15) | 0.82 | - | - | - | - |

a Multivariable analysis of SPM occurrence using competing risk model analysis (excluding patients with SPMs occurring within less than 6 months from diagnosis). Significant values (P <0.05) are highlighted in bold.

b Multivariable analysis of SPM occurrence using competing risk model analysis (including patients with SPMs occurring within less than 6 months from diagnosis). Significant values (P <0.05) are highlighted in bold.

c Multivariable analysis of SPM occurrence using Cox proportional hazards model (excluding patients with SPMs occurring within less than 6 months from diagnosis). Significant values (P <0.05) are highlighted in bold.

d Multivariable analysis of SPM occurrence using Cox proportional hazards model (including patients with SPMs occurring within less than 6 months from diagnosis). Significant values (P <0.05) are highlighted in bold.

e Others for race represented American Indian/AK Native, Asian/Pacific Islander.

f Others for marital status represented divorced, separated, unmarried or domestic partner, widowed.
