# Supplementary material for: Risk and prognosis of second primary malignancies in patients with follicular lymphoma in the era of rituximab: A population study based on the SEER database
Source: PLoS One. 2025 May 28;20(5):e0324532. doi: 10.1371/journal.pone.0324532 (PMC12118830; doi:10.1371/journal.pone.0324532)
Supplement: S8 Table — (DOCX) [file pone.0324532.s009.docx]

S8 Table

| **characteristic** | **CP-HR^a^**  **(N=33104)** | **P-value** | **CP-HR^b^**  **(N=33610)** | **P-value** | **C-HR^c^**  **(N=33104)** | **P-value** | **C-HR^d^**  **(N=33610)** | **P-value** |
| --- | --- | --- | --- | --- | --- | --- | --- | --- |
| **Sex** |  |  |  |  |  |  |  |  |
| Male | 1 |  | 1 |  | 1 |  | 1 |  |
| Female | 0.89(0.85-0.94) | **<0.001** | 0.89(0.85-0.94) | **<0.001** | 0.89(0.84-0.93) | **<0.001** | 0.88(0.84-0.93) | **<0.001** |
| **Age at diagnosis** |  |  |  |  |  |  |  |  |
| 15-39 | 1 |  | 1 |  | 1 |  | 1 |  |
| 40-60 | 1.53(1.30-1.81) | **<0.001** | 1.53(1.30-1.81) | <0.001 | 1.58(1.34-1.87) | **<0.001** | 1.58(1.34-1.87) | **<0.001** |
| >60 | 3.44(2.92-4.04) | **<0.001** | 3.43(2.91-4.03) | <0.001 | 4.05(3.44-4.76) | **<0.001** | 4.05(3.44-4.76) | **<0.001** |
| **Race** |  |  |  |  |  |  |  |  |
| White | 1 |  | 1 |  | 1 |  | 1 |  |
| Black | 1.17(1.04-1.31) | **0.008** | 1.17(1.04-1.31) | **0.09** | 1.17(1.04-1.31) | **0.009** | 1.16(1.04-1.30) | **0.011** |
| Others^e^ | 0.93(0.82-1.05) | 0.22 | 0.93(0.83-1.05) | 0.25 | 0.91(0.81-1.03) | 0.125 | 0.91(0.81-1.03) | 0.138 |
| **Ethnicity** |  |  |  |  |  |  |  |  |
| Hispanics | 1 |  | 1 |  | 1 |  | 1 |  |
| Non-Hispanics | 1.06(0.97-1.15) | 0.18 | 1.06(0.97-1.15) | 0.18 | 1.07(0.99-1.17) | 0.105 | 1.07(0.99-1.17) | 0.1 |
| **FL-subtype** |  |  |  |  |  |  |  |  |
| Grade1-2 | 1 |  | 1 |  | 1 |  | 1 |  |
| Grade3 | 1.17(1.09-1.25) | **<0.001** | 1.18(1.10-1.26) | **<0.001** | 1.17(1.10-1.26) | **<0.001** | 1.18(1.10-1.27) | **<0.001** |
| Grade NOS | 1.34(1.26-1.42) | **<0.001** | 1.33(1.26-1.41) | **<0.001** | 1.36(1.28-1.44) | **<0.001** | 1.36(1.28-1.44) | **<0.001** |
| **Ann Arbor stage** |  |  |  |  |  |  |  |  |
| I/ II | 1 |  | 1 |  | 1 |  | 1 |  |
| III/IV | 1.76(1.66-1.86) | **<0.001** | 1.77(1.67-1.87) | **<0.001** | 1.77(1.67-1.88) | **<0.001** | 1.78(1.68-1.89) | **<0.001** |
| Unknown | 0.91(0.83-1.00) | **0.042** | 0.91(0.83-1.00) | **0.046** | 0.94(0.86-1.04) | 0.234 | 0.95(0.86-1.04) | 0.259 |
| **Radiotherapy** | 1.58(1.47-1.71) | **<0.001** | 1.58(1.47-1.70) | **p<0.001** | 1.62(1.50-1.74) | **<0.001** | 1.62(1.50-1.74) | **<0.001** |
| **Chemotherapy** | 0.65(0.61-0.69) | **<0.001** | 0.65(0.61-0.69) | **p<0.001** | 0.66(0.62-0.70) | **<0.001** | 0.65(0.62-0.69) | **<0.001** |
| **Surgery** | 1.15(1.09-1.21) | **<0.001** | 1.16(1.10-1.22) | **p<0.001** | 1.18(1.12-1.24) | **<0.001** | 1.18(1.12-1.25) | **<0.001** |
| **Marital status** |  |  |  |  |  |  |  |  |
| Married | 1 |  | 1 |  | 1 |  | 1 |  |
| Single | 1.05(0.96-1.14) | 0.27 | 1.06(0.98-1.15) | 0.15 | 1.05(0.97-1.14) | 0.223 | 1.07(0.98-1.15) | 0.126 |
| Others^f^ | 1.63(1.54-1.73) | **<0.001** | 1.63(1.54-1.73) | **<0.001** | 1.77(1.67-1.88) | **<0.001** | 1.77(1.67-1.88) | **<0.001** |
| **Income** |  |  |  |  |  |  |  |  |
| <$65,000 | 1 |  | 1 |  | 1 |  | 1 |  |
| $65,000 - $74,999 | 0.95(0.89-1.02) | 0.15 | 0.95(0.89-1.02) | 0.13 | 0.94(0.88-1.00) | 0.058 | 0.94(0.88-1.00) | **0.048** |
| ≥$75,000 | 0.83(0.78-0.88) | **<0.001** | 0.82(0.78-0.88) | **<0.001** | 0.80(0.75-0.85) | **<0.001** | 0.80(0.75-0.85) | **<0.001** |
| **Rural-Ubran** |  |  |  |  |  |  |  |  |
| Metropolitan areas | 1 |  | 1 |  | 1 |  | 1 |  |
| Nonmetropolitan counties | 1.25(1.17-1.34) | **<0.001** | 1.24(1.16-1.33) | **<0.001** | 1.27(1.19-1.36) | **<0.001** | 1.26(1.18-1.35) | **<0.001** |
| **Site** |  |  |  |  |  |  |  |  |
| NHL – Extranodal | 1 |  | 1 |  | 1 |  | 1 |  |
| NHL – Nodal | 1.52(1.40-1.66) | **<0.001** | 1.53(1.40-1.67) | **<0.001** | 1.52(1.40-1.66) | **<0.001** | 1.53(1.40-1.67) | **<0.001** |
| **Year of diagnosis** |  |  |  |  |  |  |  |  |
| 2000-2004 | 1 |  | 1 |  | 1 |  | 1 |  |
| 2005-2009 | 0.73(0.69-0.78) | **<0.001** | 0.73(0.69-0.78) | **<0.001** | 0.74(0.70-0.79) | **<0.001** | 0.75(0.70-0.79) | **<0.001** |
| 2010-2014 | 0.55(0.51-0.59) | **<0.001** | 0.55(0.51-0.59) | **<0.001** | 0.57(0.52-0.61) | **<0.001** | 0.57(0.53-0.62) | **<0.001** |
| 2015-2019 | 0.44(0.40-0.49) | **<0.001** | 0.45(0.41-0.49) | **<0.001** | 0.47(0.42-0.51) | **<0.001** | 0.47(0.43-0.52) | **<0.001** |
| 2020 | 0.39(0.25-0.60) | **<0.001** | 0.38(0.25-0.59) | **<0.001** | 0.42(0.27-0.65) | **<0.001** | 0.41(0.27-0.63) | **<0.001** |
| **spm** |  |  |  |  |  |  |  |  |
| No | 1 |  | 1 |  | 1 |  | 1 |  |
| Yes | 0.79(0.73-0.86) | **<0.001** | 0.83(0.77-0.89) | **<0.001** | 0.81(0.75-0.88) | **<0.001** | 0.86(0.80-0.93) | **<0.001** |
| **B symptom** |  |  |  |  |  |  |  |  |
| None | 1 |  | 1 |  | 1 |  | 1 |  |
| Any | 1.83(1.62-2.05) | **<0.001** | 1.80(1.61-2.02) | **<0.001** | 1.85(1.65-2.08) | **<0.001** | 1.83(1.63-2.05) | **<0.001** |
| Unknown | 2.05(1.90-2.20) | **<0.001** | 2.01(1.87-2.17) | **<0.001** | 1.99(1.84-2.14) | **<0.001** | 1.95(1.82-2.10) | **<0.001** |
| **Diagnosis-to-treatment** |  |  |  |  |  |  |  |  |
| ≤1month | 1 |  | 1 |  | 1 |  | 1 |  |
| >1month | 0.72(0.67-0.77) | **<0.001** | 0.72(0.67-0.77) | **<0.001** | 0.72(0.67-0.77) | **<0.001** | 0.72(0.67-0.77) | **<0.001** |

a Univariate competing risks analysis of predictors affecting lymphoma-specific survival (excluding patients with SPMs occurring within less than 6 months from diagnosis). Significant values (P <0.05) are highlighted in bold.

b Univariate competing risks analysis of predictors affecting lymphoma-specific survival (including patients with SPMs occurring within less than 6 months from diagnosis). Significant values (P <0.05) are highlighted in bold.

c Univariate Cox regression analysis of predictors affecting lymphoma-specific survival (excluding patients with SPMs occurring within less than 6 months from diagnosis). Significant values (P <0.05) are highlighted in bold.

d Univariate Cox regression analysis of predictors affecting lymphoma-specific survival (including patients with SPMs occurring within less than 6 months from diagnosis). Significant values (P <0.05) are highlighted in bold.

e Others for race represented American Indian/AK Native, Asian/Pacific Islander.

f Others for marital status represented divorced, separated, unmarried or domestic partner, widowed.
