# Supplementary material for: Risk and prognosis of second primary malignancies in patients with follicular lymphoma in the era of rituximab: A population study based on the SEER database
Source: PLoS One. 2025 May 28;20(5):e0324532. doi: 10.1371/journal.pone.0324532 (PMC12118830; doi:10.1371/journal.pone.0324532)
Supplement: S9 Table — (DOCX) [file pone.0324532.s010.docx]

S9 Table

| **characteristic** | **CP-HR^a^**  **(N=33104)** | **P-value** | **CP-HR^b^**  **(N=33610)** | **P-value** | **C-HR^c^**  **(N=33104)** | **P-value** | **C-HR^d^**  **(N=33610)** | **P-value** |
| --- | --- | --- | --- | --- | --- | --- | --- | --- |
| All patients |  |  |  |  |  |  |  |  |
| **Sex** |  |  |  |  |  |  |  |  |
| Male | 1 |  | 1 |  | 1 |  | 1 |  |
| Female | 0.76(0.72-0.80) | **<0.001** | 0.76(0.72-0.80) | **<0.001** | 0.73(0.69-0.77) | **<0.001** | 0.73(0.69-0.77) | **<0.001** |
| **Age at diagnosis** |  |  |  |  |  |  |  |  |
| 15-39 | 1 |  | 1 |  | 1 |  | 1 |  |
| 40-60 | 1.67(1.41-1.97) | **<0.001** | 1.67(1.41-1.97) | **<0.001** | 1.72(1.46-2.04) | **<0.001** | 1.72(1.45-2.03) | **<0.001** |
| >60 | 4.01(3.40-4.73) | **<0.001** | 4.01(3.40-4.72) | **<0.001** | 4.72(4.00-5.58) | **<0.001** | 4.72(4.00-5.57) | **<0.001** |
| **Race** |  |  |  |  |  |  |  |  |
| White | 1 |  | 1 |  | 1 |  | 1 |  |
| Black | 1.23(1.09-1.38) | **<0.001** | 1.22(1.08-1.37) | **0.001** | 1.23(1.09-1.38) | **0.001** | 1.22(1.08-1.37) | **<0.00**1 |
| Others^e^ | 1.08(0.95-1.22) | 0.24 | 1.08(0.96-1.23) | 0.19 | 1.05(0.93-1.19) | 0.392 | 1.06(0.94-1.20) | 0.336 |
| **Ethnicity** |  |  |  |  |  |  |  |  |
| Hispanics | 1 |  | 1 |  | 1 |  | 1 |  |
| Non-Hispanics | 0.90(0.83-0.98) | **0.018** | 0.90(0.83-0.98) | **0.018** | 0.91(0.83-0.99) | **0.027** | 0.91(0.83-0.99) | **0.027** |
| **FL-subtype** |  |  |  |  |  |  |  |  |
| Grade1-2 | 1 |  | 1 |  | 1 |  | 1 |  |
| Grade3 | 1.09(1.01-1.17) | **0.02** | 1.10(1.02-1.18) | **0.009** | 1.10(1.02-1.18) | **0.009** | 1.11(1.03-1.19) | **0.004** |
| Grade NOS | 1.25(1.18-1.33) | **<0.001** | 1.25(1.17-1.32) | **<0.001** | 1.269(1.20-1.35) | **<0.001** | 1.27(1.19-1.34) | **<0.001** |
| **Ann Arbor stage** |  |  |  |  |  |  |  |  |
| I/ II(1) | 1 |  | 1 |  | 1 |  | 1 |  |
| III/IV(2) | 1.57(1.47-1.68) | **<0.001** | 1.58(1.48-1.69) | **<0.001** | 1.60(1.50-1.70) | **<0.001** | 1.61(1.51-1.71) | <**0.001** |
| Unknown | 1.08(0.96-1.22) | 0.17 | 1.09(0.97-1.22) | 0.17 | 1.10(0.98-1.24) | 0.099 | 1.10(0.98-1.24) | 0.095 |
| **Radiotherapy** | 1.15(1.06-1.25) | **<0.001** | 1.15(1.06-1.25) | **<0.001** | 1.176(1.08-1.28) | **<0.001** | 1.18(1.09-1.28) | **<0.001** |
| **Chemotherapy** | 0.81(0.75-0.87) | **<0.001** | 0.81(0.76-0.87) | **<0.001** | 0.83(0.77-0.89) | **<0.001** | 0.83(0.78-0.89) | **<0.001** |
| **Surgery** | 1.23(1.15-1.31) | **<0.001** | 1.23(1.16-1.31) | **<0.001** | 1.25(1.17-1.33) | **<0.001** | 1.25(1.18-1.33) | **<0.001** |
| **Marital status** |  |  |  |  |  |  |  |  |
| Married | 1 |  | 1 |  | 1 |  | 1 |  |
| Single | 1.29(1.19-1.41) | **<0.001** | 1.31(1.21-1.43) | **<0.001** | 1.34(1.24-1.46) | **<0.001** | 1.36(1.25-1.48) | **<0.001** |
| Others^f^ | 1.50(1.41-1.59) | **<0.001** | 1.50(1.41-1.59) | **<0.001** | 1.63(1.53-1.73) | **<0.001** | 1.63(1.54-1.73) | **<0.001** |
| **Income** |  |  |  |  |  |  |  |  |
| <$65,000 | 1 |  | 1 |  | 1 |  | 1 |  |
| $65,000 - $74,999 | 0.99(0.92-1.06) | 0.75 | 0.98(0.91-1.06) | 0.66 | 0.98(0.91-1.06) | 0.614 | 0.98(0.91-1.05) | 0.517 |
| ≥$75,000 | 0.86(0.81-0.93) | **<0.001** | 0.86(0.80-0.92) | **<0.001** | 0.848(0.79-0.91) | **<0.001** | 0.84(0.79-0.91) | **<0.001** |
| **Rural-Ubran** |  |  |  |  |  |  |  |  |
| Metropolitan areas | 1 |  | 1 |  | 1 |  | 1 |  |
| Nonmetropolitan counties | 1.11(1.02-1.21) | **0.014** | 1.10(1.01-1.20) | **0.021** | 1.11(1.02-1.20) | **0.012** | 1.10(1.02-1.19) | **0.019** |
| **Site** |  |  |  |  |  |  |  |  |
| NHL – Extranodal | 1 |  | 1 |  | 1 |  | 1 |  |
| NHL – Nodal | 1.26(1.15-1.38) | **<0.001** | 1.26(1.15-1.38) | **<0.001** | 1.26(1.15-1.38) | **<0.001** | 1.26(1.15-1.38) | **<0.001** |
| **Year of diagnosis** |  |  |  |  |  |  |  |  |
| 2000-2004 | 1 |  | 1 |  | 1 |  | 1 |  |
| 2005-2009 | 0.66(0.62-0.71) | **<0.001** | 0.67(0.63-0.71) | **<0.001** | 0.67(0.63-0.72) | **<0.001** | 0.68(0.64-0.72) | **<0.001** |
| 2010-2014 | 0.58(0.50-0.66) | **<0.001** | 0.57(0.50-0.66) | **<0.001** | 0.60(0.52-0.68) | **<0.001** | 0.60(0.52-0.68) | **<0.001** |
| 2015-2019 | 0.55(0.46-0.65) | **<0.001** | 0.55(0.46-0.65) | **<0.001** | 0.58(0.48-0.68) | **<0.001** | 0.58(0.49-0.69) | **<0.001** |
| 2020 | 0.53(0.33-0.85) | **0.008** | 0.52(0.32-0.83) | **0.006** | 0.58(0.36-0.92) | **0.020** | 0.56(0.36-0.90) | **0.015** |
| **spm** |  |  |  |  |  |  |  |  |
| No | 1 |  | 1 |  | 1 |  | 1 |  |
| Yes | 0.66(0.61-0.71) | **<0.001** | 0.69(0.64-0.75) | **<0.001** | 0.65(0.6-0.71) | **<0.001** | 0.70(0.65-0.75) | **<0.001** |
| **B symptom** |  |  |  |  |  |  |  |  |
| None | 1 |  | 1 |  | 1 |  | 1 |  |
| Any | 1.56(1.39-1.76) | **<0.001** | 1.55(1.37-1.73) | **<0.001** | 1.60(1.43-1.80) | **<0.001** | 1.58(1.41-1.78) | **<0.001** |
| Unknown | 1.51(1.31-1.74) | **<0.001** | 1.48(1.28-1.70) | **<0.001** | 1.53(1.33-1.76) | **<0.001** | 1.50(1.30-1.72) | **<0.001** |
| **Diagnosis-to-treatment-time** |  |  |  |  |  |  |  |  |
| ≤1month | 1 |  | 1 |  | 1 |  | 1 |  |
| >1month | 0.71(0.66-0.77) | **<0.001** | 0.71(0.66-0.77) | **<0.001** | 0.70(0.65-0.76) | **<0.001** | 0.70(0.65-0.76) | **<0.001** |

a Multivariable competing risks analysis of predictors affecting lymphoma-specific survival (excluding patients with SPMs occurring within less than 6 months from diagnosis). Significant values (P <0.05) are highlighted in bold.

b Multivariable competing risks analysis of predictors affecting lymphoma-specific survival (including patients with SPMs occurring within less than 6 months from diagnosis). Significant values (P <0.05) are highlighted in bold.

c Multivariable Cox regression analysis of predictors affecting lymphoma-specific survival (excluding patients with SPMs occurring within less than 6 months from diagnosis). Significant values (P <0.05) are highlighted in bold.

d Multivariable Cox regression analysis of predictors affecting lymphoma-specific survival (including patients with SPMs occurring within less than 6 months from diagnosis). Significant values (P <0.05) are highlighted in bold.

e Others for race represented American Indian/AK Native, Asian/Pacific Islander.

f Others for marital status represented divorced, separated, unmarried or domestic partner, widowed.
