# Supplementary material for: Risk and prognosis of second primary malignancies in patients with follicular lymphoma in the era of rituximab: A population study based on the SEER database
Source: PLoS One. 2025 May 28;20(5):e0324532. doi: 10.1371/journal.pone.0324532 (PMC12118830; doi:10.1371/journal.pone.0324532)
Supplement: S10 Table — (DOCX) [file pone.0324532.s011.docx]

S10 Table

| **characteristic** | **C-HR^a^**  **(N=33610)** | **p-value** | **C-HR^b^**  **(N=33104)** | **p-value** |
| --- | --- | --- | --- | --- |
| **Sex** |  |  |  |  |
| Male | 1 |  | 1 |  |
| Female | 0.91(0.88-0.94) | **<0.001** | 0.91(0.88-0.94) | **<0.001** |
| **Age at diagnosis** |  |  |  |  |
| 15-39 | 1 |  | 1 |  |
| 40-60 | 1.84(1.61-2.10) | **<0.001** | 1.86(1.63-2.13) | **<0.001** |
| >60 | 6.43(5.65-7.32) | **<0.001** | 6.48(5.68-7.38) | **<0.001** |
| **Race** |  |  |  |  |
| White | 1 |  | 1 |  |
| Black | 1.05(0.97-1.15) | 0.223 | 1.06(0.98-1.16) | 0.153 |
| Others^c^ | 0.83(0.76-0.90) | **<0.001** | 0.83(0.76-0.91) | **<0.001** |
| **Ethnicity** |  |  |  |  |
| Hispanics | 1 |  | 1 |  |
| Non-Hispanics | 1.2(1.13-1.28) | **<0.001** | 1.20(1.13-1.27) | **<0.001** |
| **FL-subtype** |  |  |  |  |
| Grade1-2 | 1 |  | 1 |  |
| Grade3 | 1.13(1.07-1.18) | **<0.001** | 1.124(1.07-1.18) | **<0.001** |
| Grade NOS | 1.26(1.21-1.31) | **<0.001** | 1.26(1.21-1.31) | **<0.001** |
| **Ann Arbor stage** |  |  |  |  |
| I/ II | 1 |  | 1 |  |
| III/IV | 1.30(1.26-1.36) | **<0.001** | 1.31(1.26-1.36) | **<0.001** |
| Unknown | 0.98(0.92-1.05) | 0.53 | 0.98(0.92-1.05) | 0.544 |
| **Radiotherapy** | 1.34(1.27-1.40) | **<0.001** | 1.33(1.27-1.40) | **<0.001** |
| **chem** | 0.87(0.83-0.90) | **<0.001** | 0.86(0.83-0.90) | **<0.001** |
| **Surgery** | 1.13(1.09-1.17) | **<0.001** | 1.13(1.09-1.17) | **<0.001** |
| **Marital status** |  |  |  |  |
| Married | 1 |  | 1 |  |
| Single | 1.02(0.96-1.08) | 0.576 | 1.01(0.96-1.08) | 0.637 |
| Others^d^ | 1.92(1.84-2.00) | **<0.001** | 1.92(1.84-2.00) | **<0.001** |
| **Income** |  |  |  |  |
| <$65,000 | 1 |  | 1 |  |
| $65,000 - $74,999 | 0.91(0.87-0.96) | **<0.001** | 0.92 (0.87-0.96) | **<0.001** |
| ≥$75,000 | 0.78(0.75-0.81) | **<0.001** | 0.776(0.74-0.81) | **<0.001** |
| **Rural-Ubran** |  |  |  |  |
| Metropolitan areas | 1 |  | 1 |  |
| Nonmetropolitan counties | 1.25(1.19-1.31) | **<0.001** | 1.25(1.19-1.32) | **<0.001** |
| **Site** |  |  |  |  |
| NHL – Extranodal | 1 |  | 1 |  |
| NHL – Nodal | 1.16(1.10-1.22) | **<0.001** | 1.16(1.10-1.22) | **<0.001** |
| **Year of diagnosis** |  |  |  |  |
| 2000-2004 | 1 |  | 1 |  |
| 2005-2009 | 0.86(0.83-0.90) | **<0.001** | 0.86(0.82-0.90) | **<0.001** |
| 2010-2014 | 0.73(0.69-0.77) | **<0.001** | 0.72(0.68-0.76) | **<0.001** |
| 2015-2019 | 0.62(0.58-0.67) | **<0.001** | 0.62(0.50-0.66) | **<0.001** |
| 2020 | 0.60(0.43-0.84) | **0.003** | 0.62(0.44-0.86) | **0.005** |
| **spm** |  |  |  |  |
| No | 1 |  | 1 |  |
| Yes | 1.33(1.27-1.39) | **<0.001** | 1.26(1.21-1.33) | **<0.001** |
| **B symptom** |  |  |  |  |
| None | 1 |  | 1 |  |
| Any | 1.48(1.36-1.61) | **<0.001** | 1.49(1.37-1.63) | **<0.001** |
| Unknown | 1.50(1.420-1.58) | **<0.001** | 1.51(1.43-1.60) | **<0.001** |
| **Diagnosis-to-treatment** |  |  |  |  |
| ≤1month | 1 |  | 1 |  |
| >1month | 0.85(0.81-0.89) | **<0.001** | 0.85(0.81-0.89) | **<0.001** |

a Univariate Cox regression analysis of predictors affecting overall survival (including patients with SPMs occurring within less than 6 months from diagnosis). Significant values (P <0.05) are highlighted in bold.

b Univariate Cox regression analysis of predictors affecting overall survival (excluding patients with SPMs occurring within less than 6 months from diagnosis). Significant values (P <0.05) are highlighted in bold.

c Others for race represented American Indian/AK Native, Asian/Pacific Islander.

d Others for marital status represented divorced, separated, unmarried or domestic partner, widowed.
