# Supplementary material for: Risk and prognosis of second primary malignancies in patients with follicular lymphoma in the era of rituximab: A population study based on the SEER database
Source: PLoS One. 2025 May 28;20(5):e0324532. doi: 10.1371/journal.pone.0324532 (PMC12118830; doi:10.1371/journal.pone.0324532)
Supplement: S11 Table — (DOCX) [file pone.0324532.s012.docx]

S11 Table

| **characteristic** | **C-HR^a^**  **(N=33610)** | **p-value** | **C-HR^b^**  **(N=33104)** | **p-value** |
| --- | --- | --- | --- | --- |
| **Sex** |  |  |  |  |
| Male | 1 |  | 1 |  |
| Female | 0.74(0.71-0.77) | **<0.001** | 0.73(0.71-0.76) | **<0.001** |
| **Age at diagnosis** |  |  |  |  |
| 15-39 | 1 |  | 1 |  |
| 40-60 | 1.93(1.69-2.21) | **<0.001** | 1.96(1.72-2.25) | **<0.001** |
| >60 | 6.81(5.97-7.77) | **<0.001** | 6.92(6.06-7.90) | **<0.001** |
| **Race** |  |  |  |  |
| White | 1 |  | 1 |  |
| Black | 1.12(1.03-1.22) | **0.01** | 1.13(1.04-1.23) | **0.006** |
| Others^c^ | 0.93(0.85-1.02) | 0.125 | 0.93(0.85-1.02) | 0.120 |
| **Ethnicity** |  |  |  |  |
| Hispanics | 1 |  | 1 |  |
| Non-Hispanics | 1.00(0.94-1.07) | 0.969 | 1.00(0.94-1.07) | 0.977 |
| **FL-subtype** |  |  |  |  |
| Grade1-2 | 1 |  | 1 |  |
| Grade3 | 1.09(1.033-1.14) | **0.001** | 1.08(1.03-1.14) | **0.003** |
| Grade NOS | 1.19(1.14-1.24) | **<0.001** | 1.19(1.14-1.24) | **<0.001** |
| **Ann Arbor stage** |  |  |  |  |
| I/ II | 1 |  | 1 |  |
| III/IV | 1.28(1.22-1.33) | **<0.001** | 1.28(1.23-1.34) | **<0.001** |
| Unknown | 1.10(1.02-1.19) | **0.020** | 1.10(1.02-1.19) | **0.020** |
| **Radiotherapy** | 1.17(1.11-1.23) | **<0.001** | 1.16(1.10-1.22) | **<0.001** |
| **Chemotherapy** | 0.98(0.94-1.03) | 0.45 | 0.98(0.94-1.03) | 0.395 |
| **Surgery** | 1.19(1.14-1.25) | **<0.001** | 1.20(1.14-1.25) | **<0.001** |
| **Marital status** |  |  |  |  |
| Married | 1 |  | 1 |  |
| Single | 1.42(1.33-1.50) | **<0.001** | 1.41(1.33-1.50) | **<0.001** |
| Others^d^ | 1.73(1.66-1.81) | **<0.001** | 1.73(1.66-1.80) | **<0.001** |
| **Income** |  |  |  |  |
| <$65,000 | 1 |  | 1 |  |
| $65,000 - $74,999 | 0.97(0.92-1.02 | 0.204 | 0.97(0.92-1.02) | 0.260 |
| ≥$75,000 | 0.83(0.79-0.87) | **<0.001** | 0.83(0.79-0.87) | **<0.001** |
| **Rural-Ubran** |  |  |  |  |
| Metropolitan areas | 1 |  | 1 |  |
| Nonmetropolitan counties | 1.07(1.01-1.13) | **0.028** | 1.07(1.01-1.13) | **0.027** |
| **Site** |  |  |  |  |
| NHL – Extranodal | 1 |  | 1 |  |
| NHL – Nodal | 1.11(1.05-1.18) | **<0.001** | 1.11(1.05-1.18) | **<0.001** |
| **Year of diagnosis** |  |  |  |  |
| 2000-2004 | 1 |  | 1 |  |
| 2005-2009 | 0.81(0.77-0.84) | **<0.001** | 0.80(0.77-0.84) | **<0.001** |
| 2010-2014 | 0.72(0.66-0.80) | **<0.001** | 0.72(0.65-0.80) | **<0.001** |
| 2015-2019 | 0.66(0.58-0.74) | **<0.001** | 0.65(0.57-0.74) | **<0.001** |
| 2020 | 0.67(0.47-0.96) | **0.029** | 0.69(0.48-0.98) | **0.04** |
| **spm** |  |  |  |  |
| No | 1 |  | 1 |  |
| Yes | 1.08(1.03-1.13) | **<0.001** | 1.028(0.98-1.08) | 0.272 |
| **B symptom** |  |  |  |  |
| None | 1 |  | 1 |  |
| Any | 1.41(1.29-1.53) | **<0.001** | 1.41(1.30-1.54) | **<0.001** |
| Unknown | 1.27(1.14-1.40) | **<0.001** | 1.28(1.15-1.42) | **<0.001** |
| **Diagnosis-to-treatment** |  |  |  |  |
| ≤1month | 1 |  | 1 |  |
| >1month | 0.81 (0.77-0.85) | **<0.001** | 0.80(0.76-0.85) | **<0.001** |

a Multivariable Cox regression analysis of predictors affecting overall survival (including patients with SPMs occurring within less than 6 months from diagnosis). Significant values (P <0.05) are highlighted in bold.

b Multivariable Cox regression analysis of predictors affecting overall survival (excluding patients with SPMs occurring within less than 6 months from diagnosis). Significant values (P <0.05) are highlighted in bold.

c Others for race represented American Indian/AK Native, Asian/Pacific Islander.

d Others for marital status represented divorced, separated, unmarried or domestic partner, widowed.
