# Supplementary material for: Risk and prognosis of second primary malignancies in patients with follicular lymphoma in the era of rituximab: A population study based on the SEER database
Source: PLoS One. 2025 May 28;20(5):e0324532. doi: 10.1371/journal.pone.0324532 (PMC12118830; doi:10.1371/journal.pone.0324532)
Supplement: S12 Table — (DOCX) [file pone.0324532.s013.docx]

S12 Table

| **characteristic** | **CHR^a^**  **(N=4328)** | **p-value** | **CHR^b^**  **(N=3822)** | **p-value** |
| --- | --- | --- | --- | --- |
| **Sex** |  |  |  |  |
| Male | 1 |  | 1 |  |
| Female | 1.00(0.92-1.09) | 0.991 | 1.00(0.91-1.09) | 0.907 |
| **Age at diagnosis** |  |  |  |  |
| 15-39 | 1 |  | 1 |  |
| 40-60 | 1.10(0.79-1.54) | 0.567 | 1.21(0.85-1.73) | 0.296 |
| >60 | 2.61(1.88-3.64) | **<0.001** | 2.73(1.92-3.88) | **<0.001** |
| **Race** |  |  |  |  |
| White | 1 |  | 1 |  |
| Black | 0.84(0.68-1.04) | 0.10 | 0.86(0.69-1.08) | 0.187 |
| Others^c^ | 0.82(0.66-1.02) | 0.069 | 0.82(0.65-1.03) | 0.089 |
| **Ethnicity** |  |  |  |  |
| Hispanics | 1 |  | 1 |  |
| Non-Hispanics | 0.94(0.81-1.10) | 0.455 | 0.94(0.80-1.10) | 0.452 |
| **FL-subtype** |  |  |  |  |
| Grade1-2 | 1 |  | 1 |  |
| Grade3 | 1.07(0.96-1.19) | 0.249 | 1.04(0.93-1.18) | 0.478 |
| Grade NOS | 1.10(1.00-1.20) | 0.054 | 1.07(0.97-1.19) | 0.167 |
| **Ann Arbor stage** |  |  |  |  |
| I/ II | 1 |  | 1 |  |
| III/IV | 1.15(1.06-1.25) | **0.001** | 1.14(1.04-1.25) | **0.004** |
| Unknown | 1.36(1.17-1.58) | **<0.001** | 1.31(1.11-1.55) | **0.002** |
| **Radiotherapy** | 1.26(1.13-1.39) | **<0.001** | 1.22(1.10-1.36) | **<0.001** |
| **Chemotherapy** | 0.90(0.83-0.98) | **0.018** | 0.90(0.82-0.98) | **0.019** |
| **Surgery** | 1.19(1.09-1.29) | **<0.001** | 1.19(1.09-1.29) | **<0.001** |
| **Marital status** |  |  |  |  |
| Married | 1 |  | 1 |  |
| Single | 1.08(0.94-1.23) | 0.308 | 1.05(0.90-1.22) | 0.53 |
| Others^d^ | 1.56(1.42-1.72) | **<0.001** | 1.52(1.37-1.68) | **<0.001** |
| **Income** |  |  |  |  |
| <$65,000 | 1 |  | 1 |  |
| $65,000 - $74,999 | 0.93(0.84-1.03) | 0.166 | 0.94(0.84-1.05) | 0.247 |
| ≥$75,000 | 0.79(0.72-0.87) | **<0.001** | 0.78(0.70-0.86) | **<0.001** |
| **Rural-Ubran** |  |  |  |  |
| Metropolitan areas | 1 |  | 1 |  |
| Nonmetropolitan counties | 1.16(1.04-1.29) | **0.009** | 1.18(1.05-1.33) | **0.005** |
| **Site** |  |  |  |  |
| NHL – Extranodal | 1 |  | 1 |  |
| NHL – Nodal | 1.10(0.98-1.24) | 0.104 | 1.08(0.96-1.22) | 0.206 |
| **Year of diagnosis** |  |  |  |  |
| 2000-2004 | 1 |  | 1 |  |
| 2005-2009(2) | 1.14(1.04-1.26) | **0.007** | 1.15(1.03-1.27) | **0.01** |
| 2010-2014(3) | 1.66(1.46-1.88) | **<0.001** | 1.71(1.49-1.95) | **<0.001** |
| 2015-2020(4) | 2.53(2.07-3.09) | **<0.001** | 2.85(2.25-3.61) | **<0.001** |
| **B symptom** |  |  |  |  |
| None | 1 |  | 1 |  |
| Any | 1.08(0.87-1.35) | 0.497 | 1.04(0.82-1.33) | 0.736 |
| Unknown | 0.64(0.56-0.72) | **<0.001** | 0.62(0.54-0.71) | **<0.001** |
| **Diagnosis-to-treatment** |  |  |  |  |
| ≤1month | 1 |  | 1 |  |
| >1month | 1.05(0.94-1.16) | 0.413 | 1.04(0.93-1.16) | 0.525 |

a Univariate Cox regression analysis of predictors affecting overall survival in SPMs patients (including patients with SPMs occurring within less than 6 months from diagnosis). Significant values (P <0.05) are highlighted in bold.

b Univariate Cox regression analysis of predictors affecting overall survival in SPMs patients (excluding patients with SPMs occurring within less than 6 months from diagnosis). Significant values (P <0.05) are highlighted in bold.

c Others for race represented American Indian/AK Native, Asian/Pacific Islander.

d Others for marital status represented divorced, separated, unmarried or domestic partner, widowed.
