# Supplementary material for: Risk and prognosis of second primary malignancies in patients with follicular lymphoma in the era of rituximab: A population study based on the SEER database
Source: PLoS One. 2025 May 28;20(5):e0324532. doi: 10.1371/journal.pone.0324532 (PMC12118830; doi:10.1371/journal.pone.0324532)
Supplement: S13 Table — (DOCX) [file pone.0324532.s014.docx]

S13 Table

| **characteristic** | **CHR^a^**  **(N=4328)** | **p-value** | **CHR^b^**  **(N=3822)** | **p-value** |
| --- | --- | --- | --- | --- |
| **Age at diagnosis** |  |  |  |  |
| 15-39 | 1 |  | 1 |  |
| 40-60 | 1.13(0.81-1.59) | 0.469 | 1.24(0.87-1.77) | 0.238 |
| >60 | 2.61(1.87-3.65) | **<0.001** | 2.72(1.91-3.88) | **<0.001** |
| **Race** |  |  |  |  |
| White | 1 |  | 1 |  |
| Black | 0.93(0.75-1.16) | 0.525 | 0.98(0.78-1.22) | 0.835 |
| Others^c^ | 0.87(0.70-1.09) | 0.228 | 0.86(0.68-1.08) | 0.186 |
| **FL-subtype** |  |  |  |  |
| Grade1-2 | 1 |  | 1 |  |
| Grade3 | 1.03(0.92-1.16) | 0.584 | 1.00(0.89-1.13) | 0.996 |
| Grade NOS | 1.08(0.98-1.18) | 0.132 | 1.05(0.95-1.16) | 0.388 |
| **Ann Arbor stage** |  |  |  |  |
| I/ II | 1 |  | 1 |  |
| III/IV | 1.09(0.99-1.20) | 0.078 | 1.10(0.99-1.21) | 0.076 |
| Unknown | 1.10(0.93-1.30) | 0.265 | 1.09(0.91-1.31) | 0.350 |
| **Radiotherapy** | 1.19(1.06-1.33) | **0.002** | 1.15(1.02-1.29) | **0.019** |
| **Chemotherapy** | 0.99(0.89-1.10) | 0.812 | 0.97(0.87-1.08) | 0.596 |
| **Surgery** | 1.10(1.00-1.21) | 0.051 | 1.10(0.99-1.21) | 0.067 |
| **Marital status** |  |  |  |  |
| Married | 1 |  | 1 |  |
| Single | 1.30(1.13-1.50) | **<0.001** | 1.26(1.09-1.47) | **0.002** |
| Others^d^ | 1.42(1.29-1.57) | **<0.001** | 1.38(1.25-1.53) | **<0.001** |
| **Income** |  |  |  |  |
| <$65,000 | 1 |  | 1 |  |
| $65,000 - $74,999 | 0.99(0.88-1.11) | 0.864 | 1.02(0.90-1.15) | 0.811 |
| ≥$75,000 | 0.86(0.77-0.96) | **0.007** | 0.86(0.76-0.96) | **0.008** |
| **Rural-Ubran** |  |  |  |  |
| Metropolitan areas | 1 |  | 1 |  |
| Nonmetropolitan counties | 1.07(0.94-1.22) | 0.287 | 1.09(0.96-1.25) | 0.193 |
| **Site** |  |  |  |  |
| NHL – Extranodal | 1 |  | - |  |
| NHL – Nodal | 1.052(0.931-1.189) | 0.413 | - |  |
| **Year of diagnosis** |  |  |  |  |
| 2000-2004 | 1 |  | 1 |  |
| 2005-2009 | 1.06(0.96-1.17) | 0.241 | 1.06(0.95-1.18) | 0.288 |
| 2010-2014 | 1.61(1.26-2.06) | **<0.001** | 1.74(1.34-2.27) | **<0.001** |
| 2015-2020 | 2.32(1.70-3.16) | **<0.001** | 2.73(1.93-3.85) | **<0.001** |
| **B symptom** |  |  |  |  |
| None | 1 |  | 1 |  |
| Any | 1.07(0.86-1.33) | 0.558 | 1.01(0.79-1.29) | 0.930 |
| Unknown | 1.16(0.90-1.49) | 0.258 | 1.23(0.94-1.61) | 0.139 |

a Multivariable Cox regression analysis of predictors affecting overall survival in SPMs patients (including patients with SPMs occurring within less than 6 months from diagnosis). Significant values (P <0.05) are highlighted in bold.

b Multivariable Cox regression analysis of predictors affecting overall survival in SPMs patients (excluding patients with SPMs occurring within less than 6 months from diagnosis). Significant values (P <0.05) are highlighted in bold.

c Others for race represented American Indian/AK Native, Asian/Pacific Islander.

d Others for marital status represented divorced, separated, unmarried or domestic partner, widowed.
