# Supplementary material for: Risk and prognosis of second primary malignancies in patients with follicular lymphoma in the era of rituximab: A population study based on the SEER database
Source: PLoS One. 2025 May 28;20(5):e0324532. doi: 10.1371/journal.pone.0324532 (PMC12118830; doi:10.1371/journal.pone.0324532)
Supplement: S14 Table — (DOCX) [file pone.0324532.s015.docx]

S14 Table

| **characteristic** | **CP-HR^a^**  **(N=4328)** | **p-value** | **C-HR^b^**  **(N=4328)** | **p-value** | **CP-HR^c^**  **(N=3822)** | **p-value** | **C-HR^d^**  **(N=3822)** | **p-value** |
| --- | --- | --- | --- | --- | --- | --- | --- | --- |
| **Sex** |  |  |  |  |  |  |  |  |
| Male | 1 |  | 1 |  | 1 |  | 1 |  |
| Female | 1.00(0.87-1.15) | 0.97 | 1.00(0.87-1.15) | 0.98 | 1.02(0.88-1.19) | 0.79 | 1.02(0.88-1.19) | 0.78 |
| **Age at diagnosis** |  |  |  |  |  |  |  |  |
| 15-39 | 1 |  | 1 |  | 1 |  | 1 |  |
| 40-60 | 1.04(0.60-1.79) | 0.89 | 1.07(0.62-1.83) | 0.809 | 1.03(0.58-1.82) | 0.91 | 1.09(0.63-1.91) | 0.755 |
| >60 | 1.45(0.85-2.49) | 0.17 | 1.93(1.14-3.29) | **0.015** | 1.39(0.79-2.43) | 0.25 | 1.88(1.08-3.27) | **0.0251** |
| **Race** |  |  |  |  |  |  |  |  |
| White | 1 |  | 1 |  | 1 |  | 1 |  |
| Black | 1.11(0.79-1.56) | 0.53 | 1.01(0.72-1.42) | 0.936 | 1.14(0.80-1.62) | 0.47 | 1.04(0.73-1.49) | 0.812 |
| Others^e^ | 1.10(0.78-1.56) | 0.58 | 1.02(0.72-1.44) | 0.915 | 1.06(0.73-1.53) | 0.77 | 0.984(0.68-1.43) | 0.932 |
| **Ethnicity** |  |  |  |  |  |  |  |  |
| Hispanics | 1 |  | 1 |  | 1 |  | 1 |  |
| Non-Hispanics | 1.16(0.87-1.53) | 0.31 | 1.12(0.85-1.48) | 0.439 | 1.21(0.89-1.64) | 0.22 | 1.16(0.86-1.57) | 0.345 |
| **FL-subtype** |  |  |  |  |  |  |  |  |
| Grade1-2 | 1 |  | 1 |  | 1 |  | 1 |  |
| Grade3 | 1.03(0.85-1.25) | 0.75 | 1.04(0.86-1.26) | 0.666 | 0.94(0.76-1.16) | 0.56 | 0.95(0.77-1.18) | 0.66 |
| Grade NOS | 0.95(0.81-1.12) | 0.54 | 0.98(0.83-1.16) | 0.84 | 0.93(0.78-1.11) | 0.41 | 0.96(0.80-1.14) | 0.612 |
| **Ann Arbor stage** |  |  |  |  |  |  |  |  |
| I/ II | 1 |  | 1 |  | 1 |  | 1 |  |
| III/IV | 1.40(1.21-1.62) | **<0.001** | 1.42(1.22-1.65) | **<0.001** | 1.28(1.09-1.49) | **0.003** | 1.31(1.12-1.53) | **<0.001** |
| Unknown | 1.04(0.78-1.38) | 0.81 | 1.22(0.91-1.62) | 0.18 | 0.96(0.69-1.33) | 0.80 | 1.11(0.80-1.53) | 0.525 |
| **Radiotherapy** | 1.41(1.17-1.69) | **<0.001** | 1.50(1.25-1.82) | **<0.001** | 1.40(1.15-1.70) | **<0.001** | 1.48(1.21-1.80) | **<0.001** |
| **Chemotherapy** | 0.77(0.66-0.90) | **0.001** | 0.76(0.65-0.89) | **<0.001** | 0.80(0.68-0.94) | **0.007** | 0.78(0.66-0.92) | **0.003** |
| **Surgery** | 1.17(1.02-1.35) | **0.029** | 1.25(1.09-1.44) | **0.002** | 1.12(0.96-1.30) | 0.15 | 1.20(1.03-1.40) | **0.018** |
| **Marital status** |  |  |  |  |  |  |  |  |
| Married | 1 |  | 1 |  | 1 |  | 1 |  |
| Single | 1.28(1.02-1.61) | **0.032** | 1.30(1.04-1.62) | **0.022** | 1.16(0.90-1.48) | 0.26 | 1.18(0.92-1.51) | 0.189 |
| Others^f^ | 1.30(1.10-1.54) | **0.002** | 1.51(1.28-1.79) | **<0.001** | 1.27(1.06-1.52) | **0.01** | 1.46(1.22-1.76) | **<0.001** |
| **Income** |  |  |  |  |  |  |  |  |
| <$65,000 | 1 |  | 1 |  | 1 |  | 1 |  |
| $65,000 - $74,999 | 0.87(0.72-1.05) | 0.14 | 0.85(0.70-1.02) | 0.076 | 0.86(0.71-1.05) | 0.14 | 0.84(0.69-1.03) | 0.09 |
| ≥$75,000 | 0.87(0.74-1.02) | 0.087 | 0.80(0.68-0.95) | **0.008** | 0.87(0.73-1.04) | 0.13 | 0.8(0.67-0.95) | **0.013** |
| **Rural-Ubran** |  |  |  |  |  |  |  |  |
| Metropolitan areas | 1 |  | 1 |  | 1 |  | 1 |  |
| Nonmetropolitan counties | 1.06(0.87-1.29) | 0.54 | 1.10(0.91-1.34) | 0.321 | 1.11(0.90-1.36) | 0.32 | 1.16(0.94-1.42) | 0.163 |
| **Site** |  |  |  |  |  |  |  |  |
| NHL – Extranodal | 1 |  | 1 |  | 1 |  | 1 |  |
| NHL – Nodal | 1.20(0.97-1.47) | 0.09 | 1.23(1.00-1.51) | 0.055 | 1.12(0.90-1.39) | 0.31 | 1.14(0.92-1.43) | 0.227 |
| **Year of diagnosis** |  |  |  |  |  |  |  |  |
| 2000-2004 | 1 |  | 1 |  | 1 |  | 1 |  |
| 2005-2009 | 0.91(0.78-1.07) | 0.25 | 1.04(0.88-1.23) | 0.639 | 0.89(0.75-1.06) | 0.19 | 1.03(0.86-1.22) | 0.785 |
| 2010-2014 | 0.99(0.81-1.21) | 0.92 | 1.382(1.12-1.71) | **0.003** | 0.98(0.79-1.21) | 0.84 | 1.38(1.10-1.74) | **0.005** |
| 2015-2020 | 0.92(0.65-1.29) | 0.62 | 1.65(1.15-2.36) | **0.006** | 0.75(0.48-1.19) | 0.23 | 1.48(0.93-2.37) | 0.099 |
| **B symptom** |  |  |  |  |  |  |  |  |
| None |  |  |  |  |  |  |  |  |
| Any | 1.11(0.75-1.63) | 0.61 | 1.12(0.77-1.64) | 0.562 | 1.12(0.72-1.75) | 0.61 | 1.11(0.72-1.72) | 0.629 |
| Unknown | 1.01(0.83-1.24) | 0.92 | 0.75(0.61-0.93) | **0.008** | 1.08(0.86-1.37) | 0.50 | 0.79(0.62-1.01) | 0.055 |
| **Diagnosis-to-treatment** |  |  |  |  |  |  |  |  |
| ≤1month |  |  |  |  |  |  |  |  |
| >1month | 0.95(0.78-1.14) | 0.55 | 0.99(0.82-1.19) | 0.88 | 0.95(0.78-1.16) | 0.63 | 0.99(0.81-1.21) | 0.894 |

a Univariate competing risks analysis of predictors affecting lymphoma-specific survival in Second Primary Malignancies patients (including patients with SPMs occurring within less than 6 months from diagnosis). Significant values (P <0.05) are highlighted in bold.

b Univariate Cox regression analysis of predictors affecting lymphoma-specific survival in Second Primary Malignancies patients (including patients with SPMs occurring within less than 6 months from diagnosis). Significant values (P <0.05) are highlighted in bold.

c Univariate competing risks analysis of predictors affecting lymphoma-specific survival in Second Primary Malignancies patients (excluding patients with SPMs occurring within less than 6 months from diagnosis). Significant values (P <0.05) are highlighted in bold.

d Univariate Cox regression analysis of predictors affecting lymphoma-specific survival in Second Primary Malignancies patients (excluding patients with SPMs occurring within less than 6 months from diagnosis). Significant values (P <0.05) are highlighted in bold.

e Others for race represented American Indian/AK Native, Asian/Pacific Islander.

f Others for marital status represented divorced, separated, unmarried or domestic partner, widowed.
