# Supplementary material for: Risk and prognosis of second primary malignancies in patients with follicular lymphoma in the era of rituximab: A population study based on the SEER database
Source: PLoS One. 2025 May 28;20(5):e0324532. doi: 10.1371/journal.pone.0324532 (PMC12118830; doi:10.1371/journal.pone.0324532)
Supplement: S15 Table — (DOCX) [file pone.0324532.s016.docx]

S15 Table

| **characteristic** | **CP-HR^a^**  **(N=4328)** | **p-value** | **C-HR^b^**  **(N=4328)** | **p-value** | **CP-HR^c^**  **(N=3822)** | **p-value** | **C-HR^d^**  **(N=3822)** | **p-value** |
| --- | --- | --- | --- | --- | --- | --- | --- | --- |
| **Age at diagnosis** |  |  |  |  |  |  |  |  |
| 15-39 | 1 |  | 1 |  | - | - | 1 |  |
| 40-60 | 1.07(0.62-1.86) | 0.81 | 1.12(0.65-1.92) | 0.69 | - | - | 1.12(0.64-1.96) | 0.697 |
| >60 | 1.53(0.89-2.65) | 0.13 | 2.06(1.21-3.53) | **0.008** | - | - | 1.94(1.11-3.40) | **0.019** |
| **Ann Arbor stage** |  |  |  |  |  |  |  |  |
| I/ II | 1 |  | 1 |  | 1 |  | 1 |  |
| III/IV | 1.27(1.08-1.51) | **0.004** | 1.27(1.07-1.50) | **0.005** | 1.14(0.96-1.35) | 0.14 | 1.17(0.98-1.39) | 0.075 |
| Unknown | 0.98(0.73-1.32) | 0.90 | 1.04(0.76-1.43) | 0.789 | 0.98(0.7-1.36) | 0.88 | 1.01(0.72-1.42) | 0.967 |
| **Radiotherapy** | 1.23(1.00-1.51) | **0.049** | 1.32(1.08-1.62) | **0.008** | 1.3(1.05-1.61) | **0.018** | 1.34(1.08-1.66) | **0.008** |
| **Chemotherapy** | 0.90(0.75-1.07) | 0.23 | 0.90(0.75-1.08) | 0.267 | 0.9(0.75-1.09) | 0.28 | 0.89(0.74-1.08) | 0.24 |
| **Surgery** | 1.08(0.93-1.26) | 0.32 | 1.14(0.97-1.34) | 0.108 | 1.09(0.92-1.3) | 0.31 | 1.11(0.94-1.32) | 0.218 |
| **Marital status** |  |  |  |  |  |  |  |  |
| Married | 1 |  | 1 |  | 1 |  | 1 |  |
| Single | 1.37(1.09-1.73) | **0.007** | 1.50(1.19-1.88) | **<0.001** | 1.17(0.91-1.5) | 0.22 | 1.36(1.06-1.75) | **0.017** |
| Others^f^ | 1.23(1.04-1.46) | **0.015** | 1.40(1.18-1.66) | **<0.001** | 1.26(1.05-1.51) | **0.013** | 1.37(1.14-1.64) | **<0.001** |
| **Income** |  |  |  |  |  |  |  |  |
| <$65,000 | 1 |  | 1 |  | 1 |  | 1 |  |
| $65,000 - $74,999 | 0.89(0.73-1.07) | 0.20 | 0.86(0.72-1.04) | 0.127 | 0.86(0.71-1.06) | 0.16 | 0.88(0.70-1.09) | 0.231 |
| ≥$75,000 | 0.89(0.76-1.05) | 0.17 | 0.83(0.70-0.98) | **0.027** | 0.87(0.73-1.04) | 0.13 | 1.37(1.14-1.64) | 0.086 |
| **Rural-Ubran** |  |  |  |  |  |  |  |  |
| Metropolitan areas | - | - | - | - | - | - | 1 |  |
| Nonmetropolitan counties | - | - | - | - | - | - | 1.03(0.81-1.30) | 0.811 |
| **Site** |  |  |  |  |  |  |  |  |
| NHL – Extranodal | 1 |  | 1 |  | - | - | - | - |
| NHL – Nodal | 1.02(0.82-1.27) | 0.84 | 1.04(0.84-1.29) | 0.728 | - | - | - | - |
| **Year of diagnosis** |  |  |  |  |  |  |  |  |
| 2000-2004 | - | - | 1 |  | 1 |  | 1 |  |
| 2005-2009 | - | - | 0.94(0.80-1.11) | 0.484 | 0.85(0.71-1.00) | 0.056 | 0.93(0.78-1.12) | 0.442 |
| 2010-2014 | - | - | 1.29(0.84-1.98) | 0.252 | 0.88(0.7-1.1) | 0.27 | 1.51(0.97-2.37) | 0.069 |
| 2015-2019 | - | - | 1.59(0.91-2.75) | 0.101 | 0.72(0.45-1.15) | 0.17 | 1.63(0.86-3.09) | 0.131 |
| **B symptom** |  |  |  |  |  |  |  |  |
| None | - | - | 1 |  | - | - | 1 |  |
| Any | - | - | 1.06(0.72-1.54) | 0.779 | - | - | 1.04(0.68-1.61) | 0.848 |
| Unknown | - | - | 1.15(0.74-1.79) | 0.534 | - | - | 1.38(0.86-2.21) | 0.184 |

a Multivariable competing risks analysis of predictors affecting lymphoma-specific survival in Second Primary Malignancies patients (including patients with SPMs occurring within less than 6 months from diagnosis). Significant values (P <0.05) are highlighted in bold.

b Multivariable Cox regression analysis of predictors affecting lymphoma-specific survival in Second Primary Malignancies patients (including patients with SPMs occurring within less than 6 months from diagnosis). Significant values (P <0.05) are highlighted in bold.

c Multivariable competing risks analysis of predictors affecting lymphoma-specific survival in Second Primary Malignancies patients (excluding patients with SPMs occurring within less than 6 months from diagnosis). Significant values (P <0.05) are highlighted in bold.

d Multivariable Cox regression analysis of predictors affecting lymphoma-specific survival in Second Primary Malignancies patients (excluding patients with SPMs occurring within less than 6 months from diagnosis). Significant values (P <0.05) are highlighted in bold.

e Others for race represented American Indian/AK Native, Asian/Pacific Islander.

f Others for marital status represented divorced, separated, unmarried or domestic partner, widowed.
