# Supplementary material for: Risk and prognosis of second primary malignancies in patients with follicular lymphoma in the era of rituximab: A population study based on the SEER database
Source: PLoS One. 2025 May 28;20(5):e0324532. doi: 10.1371/journal.pone.0324532 (PMC12118830; doi:10.1371/journal.pone.0324532)
Supplement: Attachment — Submitted filename: PONE-D-25-03313_reviewer.pdf [file pone.0324532.s019.pdf]

PLOS ONE

Risk and prognosis of second primary malignancies in patients with follicular lymphoma in the era of rituximab: a population study based on the SEER database

--Manuscript Draft--

**Manuscript Number:**	PONE-D-25-03313
**Article Type:**	Research Article
**Full Title:**	Risk and prognosis of second primary malignancies in patients with follicular lymphoma in the era of rituximab: a population study based on the SEER database
**Short Title:**	Risk and prognosis of second primary malignancies in patients with follicular lymphoma in the era of rituximab
**Corresponding Author:**	Caigang Xu<br>Sichuan University West China Hospital Department of Hematology<br>CHINA
**Keywords:**	Follicular lymphoma; second primary malignancies; Prognosis; SEER
**Abstract:**	Objective: Follicular lymphoma (FL) patients have achieved favorable long-term survival since the introduction of rituximab. However, the development of second primary malignancies (SPMs) indicates a poor survival prognosis for FL patients, and large-scale studies in this field remain limited. This study investigates the prognostic factors for FL patients in the rituximab era, as well as the clinical characteristics, risk factors, and prognosis for patients who developed SPMs.<br>Methods: From 2000 to 2020, a total of 33,104 patients with pathologically confirmed FL were identified within the Surveillance, Epidemiology, and End Results (SEER) database. Competing-risk regression analysis was used to assess prognostic factors for lymphoma-specific survival (LSS), risk factors for developing SPMs, and prognosis in FL patients.<br>Results: Risk factors for LSS in FL patients included age≥40 years, B symptoms, advanced disease, intranodal involvement, FL Grade 3, and chemotherapy used. 91 months after FL diagnosis, the risk of death increased for patients with SPMs. Female (HR: 0.79, 95% CI: 0.74-0.85, P<0.001), FL Grade 3 (HR: 0.89, 95% CI: 0.82-0.97, P=0.011) showed a reduced risk of developing SPMs. Age 40-60 years (HR: 2.59, 95% CI: 2.07-3.23, P<0.001), >60 years (HR: 3.48, 95% CI: 2.79-4.33, P<0.001), non-Hispanic ethnicity (HR: 1.30, 95% CI: 1.16-1.46, P<0.001), and initial treatment with radiotherapy or surgery (HR: 1.12, 95% CI: 1.03-1.22, P=0.007; HR: 1.07, 95% CI: 1.00-1.14, P=0.037) were associated with an increased risk of SPMs. For patients with SPMs, age>60 years (HR: 2.75, 95% CI: 1.93-3.92, P<0.001) and being unmarried (HR: 1.28, 95% CI: 1.10-1.48, P=0.002) were independent factors.
**Order of Authors:**	Ying Tian
	Wanxi Yang
	Juan Xu
	Yuanxiao Li
	Wenjiao Tang
	Caigang Xu
**Additional Information:**	
**Question**	**Response**
**Financial Disclosure**<br><br>Enter a financial disclosure statement that describes the sources of funding for the work included in this submission. Review the submission guidelines for detailed requirements. View published research	Yes

articles from *PLOS ONE* for specific examples.<br><br>This statement is required for submission and **will appear in the published article** if the submission is accepted. Please make sure it is accurate.<br><br>**Funded studies**<br>Enter a statement with the following details:<br>• Initials of the authors who received each award<br>• Grant numbers awarded to each author<br>• The full name of each funder<br>• URL of each funder website<br>• Did the sponsors or funders play any role in the study design, data collection and analysis, decision to publish, or preparation of the manuscript?<br><br>Did you receive funding for this work?	
Please add funding details.<br>as follow-up to "**Financial Disclosure**<br><br>Enter a financial disclosure statement that describes the sources of funding for the work included in this submission. Review the submission guidelines for detailed requirements. View published research articles from *PLOS ONE* for specific examples.<br><br>This statement is required for submission and **will appear in the published article** if the submission is accepted. Please make sure it is accurate.	Caigang Xu received funding from Sichuan Science and Technology Department for Key Research and Development Program (2019YFS0027).The funders had no role in study design, data collection and analysis, decision to publish, or preparation of the manuscript.

Powered by Editorial Manager® and ProduXion Manager® from Aries Systems Corporation

**Funded studies**<br>Enter a statement with the following details:<br>• Initials of the authors who received each award<br>• Grant numbers awarded to each author<br>• The full name of each funder<br>• URL of each funder website<br>• Did the sponsors or funders play any role in the study design, data collection and analysis, decision to publish, or preparation of the manuscript?<br><br>Did you receive funding for this work?"	
Please select the country of your main research funder (please select carefully as in some cases this is used in fee calculation).<br>as follow-up to "**Financial Disclosure**<br><br>Enter a financial disclosure statement that describes the sources of funding for the work included in this submission. Review the submission guidelines for detailed requirements. View published research articles from *PLOS ONE* for specific examples.<br><br>This statement is required for submission and **will appear in the published article** if the submission is accepted. Please make sure it is accurate.<br><br>**Funded studies**<br>Enter a statement with the following details:<br>• Initials of the authors who received each award<br>• Grant numbers awarded to each author<br>• The full name of each funder<br>• URL of each funder website<br>• Did the sponsors or funders play any role in the study design, data collection and analysis, decision to publish, or preparation of the manuscript?<br><br>Did you receive funding for this work?"	CHINA - CN
**Competing Interests**	The authors have declared that no competing interests exist.

Use the instructions below to enter a competing interest statement for this submission. On behalf of all authors, disclose any competing interests that could be perceived to bias this work—acknowledging all financial support and any other relevant financial or non-financial competing interests.<br><br>This statement is required for submission and **will appear in the published article** if the submission is accepted. Please make sure it is accurate and that any funding sources listed in your Funding Information later in the submission form are also declared in your Financial Disclosure statement.<br><br>View published research articles from *PLOS ONE* for specific examples.<br><br>**NO authors have competing interests**<br><br>Enter: *The authors have declared that no competing interests exist.*<br><br>**Authors with competing interests**<br><br>Enter competing interest details beginning with this statement:<br><br>*I have read the journal's policy and the authors of this manuscript have the following competing interests: [insert competing interests here]*<br><br>* typeset	
**Ethics Statement**<br><br>Enter an ethics statement for this submission. This statement is required if the study involved:<br><br>• Human participants<br>• Human specimens or tissue<br>• Vertebrate animals or cephalopods<br>• Vertebrate embryos or tissues	The need for obtaining written informed consent for participation in this study was not required, given that the data within the SEER database has been de-identified and is freely accessible to the public upon authorization. Consequently, ethical approval from the institutional review board of West China Hospital was waived for this research.

Powered by Editorial Manager® and ProduXion Manager® from Aries Systems Corporation

• Field research<br><br>Write "N/A" if the submission does not require an ethics statement.<br><br>General guidance is provided below. Consult the submission guidelines for detailed instructions. **Make sure that all information entered here is included in the Methods section of the manuscript.**	

**Format for specific study types**<br><br>**Human Subject Research (involving human participants and/or tissue)**<br>• Give the name of the institutional review board or ethics committee that approved the study<br>• Include the approval number and/or a statement indicating approval of this research<br>• Indicate the form of consent obtained (written/oral) or the reason that consent was not obtained (e.g. the data were analyzed anonymously)<br><br>**Animal Research (involving vertebrate animals, embryos or tissues)**<br>• Provide the name of the Institutional Animal Care and Use Committee (IACUC) or other relevant ethics board that reviewed the study protocol, and indicate whether they approved this research or granted a formal waiver of ethical approval<br>• Include an approval number if one was obtained<br>• If the study involved *non-human primates*, add *additional details* about animal welfare and steps taken to ameliorate suffering<br>• If anesthesia, euthanasia, or any kind of animal sacrifice is part of the study, include briefly which substances and/or methods were applied<br><br>**Field Research**<br><br>Include the following details if this study involves the collection of plant, animal, or other materials from a natural setting:<br>• Field permit number<br>• Name of the institution or relevant body that granted permission	
**Data Availability**<br><br>Authors are required to make all data underlying the findings described fully available, without restriction, and from the time of publication. PLOS allows rare exceptions to address legal and ethical concerns. See the PLOS Data Policy and FAQ for detailed information.	Yes - all data are fully available without restriction

A Data Availability Statement describing where the data can be found is required at submission. Your answers to this question constitute the Data Availability Statement and **will be published in the article**, if accepted.<br><br>**Important:** Stating 'data available on request from the author' is not sufficient. If your data are only available upon request, select 'No' for the first question and explain your exceptional situation in the text box.<br><br>Do the authors confirm that all data underlying the findings described in their manuscript are fully available without restriction?	
**Describe where the data may be found in full sentences. If you are copying our sample text, replace any instances of XXX with the appropriate details.**<br><br>• If the data are **held or will be held in a public repository**, include URLs, accession numbers or DOIs. If this information will only be available after acceptance, indicate this by ticking the box below. For example: *All XXX files are available from the XXX database (accession number(s) XXX, XXX.*).<br>• If the data are all contained **within the manuscript and/or Supporting Information files**, enter the following: *All relevant data are within the manuscript and its Supporting Information files.*<br>• If neither of these applies but you are able to provide **details of access elsewhere**, with or without limitations, please do so. For example:<br><br>*Data cannot be shared publicly because of [XXX]. Data are available from the XXX Institutional Data Access / Ethics Committee (contact via XXX) for researchers who meet the criteria for access to confidential data.*<br><br>*The data underlying the results presented in the study are available from (include the name of the third party*	All relevant data are within the manuscript and its Supporting Information files.

and contact information or URL).<br>• This text is appropriate if the data are owned by a third party and authors do not have permission to share the data.<br><br>* typeset	
Additional data availability information:	

Manuscript Click here to access/download;Manuscript;Manuscript-FL.docx

Risk and prognosis of second primary malignancies in patients with follicular lymphoma in the era of rituximab: a population study based on the SEER database

Ying Tian[1#a], Wanxi Yang[1#a], Juan Xu[1#a], Yuanxiao Li[2#b], Wenjiao Tang[1#a], Caigang Xu[1#a*]

1. Department of Hematology, Institute of Hematology, West China Hospital, Sichuan University, Chengdu, China

2. Department of Pediatric Gastroenterology, Lanzhou University Second Hospital, Lanzhou, China

#a Current Address: No. 37 Guoxue Xiang, District Wuhou, Chengdu, Sichuan, 10084, China

#b Current Address: 82 Cuiyingmen Street, Chengguan District, Lanzhou, Gansu Province, 730030, China

*Corresponding author

E-mail: xucaigang@wchscu.cn

Abstract

Objective: Follicular lymphoma (FL) patients have achieved favorable long-term survival since the introduction of rituximab. However, the development of second primary malignancies (SPMs) indicates a poor survival prognosis for FL patients, and large-scale studies in this field remain limited. This study investigates the prognostic factors for FL patients in the rituximab era, as well as the clinical characteristics, risk factors, and prognosis for patients who developed SPMs.

Methods: From 2000 to 2020, a total of 33,104 patients with pathologically confirmed FL were identified within the Surveillance, Epidemiology, and End Results (SEER) database. Competing-risk regression analysis was used to assess prognostic factors for lymphoma-specific survival (LSS), risk factors for developing SPMs, and prognosis in FL patients.

Results: Risk factors for LSS in FL patients included age≥40 years, B symptoms, advanced disease, intranodal involvement, FL Grade 3, and chemotherapy used. 91 months after FL diagnosis, the risk of death increased for patients with SPMs. Female (HR: 0.79, 95% CI: 0.74-0.85, P<0.001), FL Grade 3 (HR: 0.89, 95% CI: 0.82-0.97, P=0.011) showed a reduced risk of developing SPMs. Age 40-60 years (HR: 2.59, 95% CI: 2.07-3.23, P<0.001), >60 years (HR: 3.48, 95% CI: 2.79-4.33, P<0.001), non-Hispanic ethnicity (HR: 1.30, 95% CI: 1.16-1.46, P<0.001), and initial

treatment with radiotherapy or surgery (HR: 1.12, 95% CI: 1.03-1.22, P=0.007; HR: 1.07, 95% CI: 1.00-1.14, P=0.037) were associated with an increased risk of SPMs. For patients with SPMs, age>60 years (HR: 2.75, 95% CI: 1.93-3.92, P<0.001) and being unmarried (HR: 1.28, 95% CI: 1.10-1.48, P=0.002) were independent factors.

1.Introduction

Follicular lymphoma (FL), originating from the germinal center B cells in lymph nodes or lymphoid tissue, is one of the most common indolent forms of non-Hodgkin's lymphoma (NHL). In Western countries, FL accounts for about 20-35% of NHL cases[1,2]. For patients needing treatment, immunochemotherapy, particularly with the CD20 monoclonal antibody rituximab, has significantly improved FL prognosis[3,4]. However, the prolonged survival and recurrent nature of FL may increase the risk of developing second primary malignancies (SPMs), which negatively impact long-term survival[5,6], and previous studies have showed that FL patients face a higher SPMs risk than the general population, with factors such as older age, male, chemotherapy, radiotherapy, radioimmunotherapy, autologous stem cell transplantation following high-dose chemotherapy, multiple treatments, and B symptoms linked to SPM development[2,7-11].Findings on these risk factors, however, vary across studies, and due to the rarity of SPMs and the need for long-term follow-up, the clinical characteristics, survival outcomes, and prognostic factors for FL patients

after developing SPMs have not been well delineated.

In this study, we conducted a large, population-based analysis across the United States to identify risk factors for the development of SPMs and to delineate prognostic indicators for FL patients in the context of rituximab therapy, along with the clinical characteristics, risk factors, and prognostic features associated with SPMs.

2. Methods

2.1 Ethics Statement

The need for obtaining written informed consent for participation in this study was not required, given that the data within the SEER database has been de-identified and is freely accessible to the public upon authorization. Consequently, ethical approval from the institutional review board of West China Hospital was waived for this research.

2.2 Data Source

The Surveillance, Epidemiology, and End Results (SEER) database, established by the National Cancer Institute (NCI), is a public database that systematically collects, curates, and maintains epidemiological and clinical data on cancer patients across the United States (available at: http://seer.cancer.gov/seerstat/). We extracted data from the SEER database(version 8.4.3)on patients with histologically confirmed primary FL (ICD-O-3 histological codes 9690-9698) diagnosed in the United States from 2000 to 2020. This study excluded patients who met any of the

following criteria: age≤14 years, incomplete clinical or epidemiological data, diagnosis established post-mortem, SPMs diagnosed within a six-month interval following the initial FL diagnosis, SPMs of B-cell lineage, or documented relapse of FL.

2.3 Analytical variables

Gender, age at diagnosis, race, ethnicity, Grade classification, Ann Arbor stage, B symptoms, treatment strategy, time from diagnosis to treatment, marital status, income level, residential area, primary site, year of diagnosis, incidence of SPMs, and cause of death were collected from the SEER database. The occurrence of SPMs, overall survival (OS), and lymphoma-specific survival (LSS) were the endpoints of our study. SPMs were defined as malignancies occurring more than 6 months after the diagnosis of primary FL. OS was defined as the time from diagnosis to death, regardless of the cause of death. LSS was defined as the time from diagnosis to death due to lymphoma. The time to the occurrence of a SPM was defined as the interval from the diagnosis of FL to the diagnosis of the SPM. Survival time was the duration from the initial diagnosis to death or to the last follow-up.

2.4 Study objectives

The study's primary goal was to elucidate risk factors for SPMs in FL patients, leveraging the specified variables. Subsequently, the research sought to determine predictors of overall and lymphoma-specific survival

in FL patients with and without subsequent SPMs. Additionally, the analysis aimed to contrast mortality etiologies between FL cohorts with and without SPMs.

2.5 Statistical Analysis

Baseline characteristics were analyzed using the Pearson $\chi 2$ test or Fisher's exact test for unordered categorical variables, ordinal categorical variables were compared using the rank-sum test. Continuous variables, such as age and time to development of SPMs, were described using median values, interquartile ranges, and the range of maximum and minimum values. Survival analysis was conducted using the Kaplan-Meier method, and the log-rank test and the Landmark test were used to compare the survival curves of the two groups.

Given that patient mortality terminates the possibility of developing SPMs, death was considered a competing event for the occurrence of SPMs. The competing-risk regression analysis was employed to assess the risk factors for the development of SPMs in FL patients. Similarly, the competing-risk regression analysis was also used to assess the risk factors for lymphoma-specific mortality, with death from non-lymphoma diseases considered as a competing event for lymphoma-specific death. Cox hazards proportional analyses were performed to determine risk factors for all-cause mortality in FL patients and those with SPMs. Factors found to be statistically significant ($P<0.05$), marginally significant ($P\leqslant 0.2$), and

those considered clinically relevant by the researchers were included in the multivariate analysis. P<0.05 was indicative of statistical significance. All statistical analyses were performed using R software (version 4.4.1).

3.Results

3.1 Epidemiology

Data from the SEER registry reveals that the incidence rate of FL in the American population decreased from 3.7 per 100,000 in 2000 to 2.8 per 100,000 in 2020, with a highest rate of 4.1 per 100,000 seen in year 2003-2004 and year 2007. The annual percent change (APC) from 2000 to 2020 was -1.6%.

In 2000, the incidence rate was 4.0 per 100,000 in males and 3.5 per 100,000 in females, peaking at 4.5 and 3.9 per 100,000 in year 2004 and year 2007, respectively. By 2020, these rates had gradually declined to 3.0 per 100,000 for males and 2.6 per 100,000 for females, with males generally showing a higher average incidence (3.8 per 100,000 versus 3.3 per 100,000).

Among different age groups, those aged ≥75 years, 65-74 years, 55-64 years, and 45-54 years saw peak incidence in year 2003-2004, reaching 12.6, 12.5, 8.7, and 4.6 per 100,000, respectively, but these rates decreased by 2020 to 8.7, 8.0, 5.2, and 3.1 per 100,000. The 15-44 age group had a relatively stable rate between 0.6 and 0.9 per 100,000 from 2000 to 2020. Individuals aged ≥65 consistently had the highest incidence among all age

groups (12.5 per 100,000 versus 7.0 per 100,000 versus 3.9 per 100,000 versus 0.8 per 100,000) (Supplementary Figure S1, Supplementary Table S1).

By race and primary tumor site, FL incidence was higher in White individuals (4.0 per 100,000 versus 1.6 per 100,000 versus 1.8 per 100,000), and more patients with primary involvement of the lymph nodes (3.0 per 100,000 versus 0.5 per 100,000) (Supplementary Table S1).

3.2 Clinical Characteristics

From 2000 to 2020, a total of 33,104 FL patients were included in the study. The median age at diagnosis was 62 years (IQR 52-72years), and the gender distribution was approximately equal (50.4% male, 49.6% female). Most patients were White (29,671/33,104, 89.6%), Grade 1-2 (17,404/33,104, 52.6%), and with advanced disease (13,757/33,104, 41.6%).

Among the cohort, 3,822 individuals (11.5%) with FL developed SPMs, with a median age at FL diagnosis of 70 years (IQR 62-77 years). In contrast, 29,282 patients (88.5%) remained free of SPMs, with a median age at FL diagnosis of 61 years (IQR 52-72 years). Compared with non-SPMs group, patients with SPMs were more likely to be male (55.3% vs. 49.8%, $P<0.0001$), older (median age 70 years vs. 61 years), non-Hispanic (91.8% vs. 86.9%, $P<0.001$), and White (91.6% vs. 89.4%, $P<0.001$). SPMs group also included more non-urban residents (15.0% vs. 13.3%,

P=0.001), more advanced disease (47.5% vs. 40.8%, P<0.001), a higher frequency of extranodal primary sites (15.0% vs. 13.5%, P=0.006), and less B symptoms (5.6% vs. 10.1%, P<0.001). A significantly higher proportion of patients in the SPMs group received treatment within one month of FL diagnosis (80.7% vs. 76.0%, P<0.001). A greater percentage of individuals with SPMs underwent radiotherapy (21.5% vs. 18.9%, P<0.001) or surgery (53.1% vs. 44.4%, P<0.001) (Table 1).

Further analysis of treatment modalities and primary lymphoma sites showed that the SPMs group more frequently received combination therapy (38.8% vs. 31.6%, P<0.001) than the non-SPMs group (Supplementary Table S2). The non-SPMs group had a higher proportion of patients with primary sites in intraperitoneal lymph nodes and multiple regions involved at diagnosis (8.7% vs. 7.2%, P=0.002; 42.4% vs. 40.3%, P=0.012), whereas the SPMs group more often had primary sites in the head, face, neck lymph nodes, and skin (13.1% vs. 10.5%, P<0.001; 2.8% vs. 2.2%, P=0.018) (Supplementary Table S3).

Table 1 Characteristics of patients with follicular lymphoma at diagnosis: Surveillance, Epidemiology, and End Results (SEER) 2000-2020.

**characteristic(N,%)**	**All patients**	**non-SPMs**	**SPMs**	**P value***	**time to SPMs, m**		
					**Median†**	**IQR**	**Range**
All patients	N=33104	29282 (88.5%)	3822 (11.5%)	-	61	29-105	6-245
**Sex**							
Male	16683 (50.4%)	14569 (49.8%)	2114 (55.3%)	**<0.001**	60	29-106	6-245

Female	16421 (49.6%)	14713 (50.2%)	1708 (44.7%)		62	29-105	6-240
**Age at diagnosis**							
15-39	1955 (5.9%)	1872 (6.4%)	83 (2.2%)	**<0.001**	83	53-138	8-245
40-60	13423 (40.5%)	11998 (41.0%)	1425 (37.3%)		78	39-129	6-240
>60	17726 (53.6%)	15412 (52.6%)	2314 (60.5%)		52.5	25-91	6-237
**Race**							
White	29671 (89.6%)	26171 (89.4%)	3500 (91.6%)	**<0.001**	60	29-105	6-245
Black	1551(4.7%)	1390 (4.7%)	161 (4.2%)		68	34-117	6-208
Others‡	1882 (5.7%)	1721 (5.9%)	161 (4.2%)		66	31.5-107	6-231
**Ethnicity**							
Hispanics	4161 (12.6%)	3846 (13.1%)	315 (8.2%)	**<0.001**	59	29-114	6-237
Non-Hispanics	28943 (87.4%)	25436 (86.9%)	3507 (91.8%)		61	29-105	6-245
**FL-subtype**							
Grade1-2	17409 (52.6%)	15346 (52.4%)	2063 (54.0%)	**0.007**	61	29-105	6-245
Grade3	6450 (19.5%)	5779 (19.7%)	671 (17.6%)		57	31-100	6-237
Grade NOS	9245 (27.9%)	8157 (29.9%)	1088 (28.5%)		63	29-103	6-237
**Ann Arbor stage**							
I/ II	10925 (33.0%)	9342 (31.9%)	1583 (41.4%)	**<0.001**	63	32-112	6-239
III/IV	13750 (41.5%)	11936 (40.8%)	1814 (47.5%)		66	32-106	6-245
Unknown	8429 (25.5%)	8004 (27.3%)	425 (11.1%)		31	15-68	6-219
**B symptom**							
None	10836 (32.7%)	10101 (34.5%)	735 (19.2%)	**<0.001**	36	18-59	6-125
Any	3176 (9.6%)	2962 (10.1%)	214 (5.6%)		36	18.75-68	6-127
Unknown	19092 (57.7%)	16219 (55.4%)	2873 (75.2%)		75	36-120	6-245
**Radiotherapy**	6365 (19.2%)	5543 (18.9%)	822 (21.5%)	**<0.001**	68.5	34-119	6-239
**Chemotherapy**	22148 (66.9%)	19574 (66.8%)	2574 (67.0%)	0.451	60	29-105	6-245
**Surgery**	15044 (45.4%)	13015 (44.4%)	2029 (53.1%)	**<0.001**	67	32-113	6-237
**Diagnosis-to-treatment-time**							
≤1month	25345 (76.6%)	22261 (76.0%)	3084(80.7%)	**<0.001**	64	31-109	6-240
＞1month	7759 (23.4%)	7021 (24.0%)	738 (19.3%)		51	24.75-90	6-245
**Income**							
<$65,000	10430 (31.5%)	9167 (31.3%)	1263 (33.0%)	0.065	59	28-100	6-245
$65,000 - $74,999	8492 (25.6%)	7530 (25.7%)	962 (25.2%)		60	30-101	6-231
≥$75,000	14182 (42.8%)	12585 (43.0%)	1597 (41.8%)		63	30-113	6-240
**Rural-Ubran**							
Metropolitan areas	28636 (86.5%)	25389 (86.7%)	3247 (85.0%)	**0.001**	61	29-105	6-245
Nonmetropolitan	4468 (13.5%)	3893 (13.3%)	575 (15.0%)		60	29-107	6-240

counties							
**Site**							
NHL – Extranodal	4527 (13.7%)	3957 (13.5%)	570 (15.0%)	**0.006**	61	31-109	6-239
NHL – Nodal	28577 (86.3%)	25325 (86.5%)	3252 (85.0%)		61	29-105	6-245
**Year of diagnosis**							
2000-2004	8299 (25.1%)	6864 (23.4%)	1435 (37.5%)	**<0.001**	86	43-139	6-245
2005-2009	8727 (26.4%)	7444 (25.4%)	1283 (33.6%)		70	34-109	6-182
2010-2014	7372 (22.3%)	6607 (22.6%)	765 (20.1%)		44	23.5-74	6-131
2015-2019	7491 (22.6%)	7159 (22.4%)	332 (8.6%)		22.5	14-36	6-68
2020	1215 (3.7%)	1208 (4.1%)	7 (0.2%)		7	6-8	6-10

IQR: interquartile range

* $\chi 2$ test was used for comparison. Significant values (P <0 .05) are highlighted in bold.

† Kruskall-Wallis test used for calculations and comparisons.

‡ Others for race represented American Indian/AK Native, Asian/Pacific Islander.

3.3 Risk of SPMs

Out of the total population, 3,844 patients (11.6%) had developed SPMs. The median time from first diagnose to SPMs was 61 months (IQR 29-105, range 6-245 months). The cumulative incidence rates for SPMs were 5.8% at 5 years, 9.4% at 10 years, and 11.1% at 15 years. The overall incidence rate of SPMs was 0.0016 per person-year. Among SPMs, lung tumors were the most common solid tumors, and myeloid/monocytic leukemias were the most common hematologic malignancies. (Supplementary Table S4).

Univariate competing-risk regression analysis revealed that radiotherapy (HR: 1.13, 95% CI: 1.04-1.22, P=0.002) and primary skin

involvement (HR: 1.28, 95% CI: 1.05-1.55, P=0.014) significantly increased the risk of developing SPMs. Age between 40-60 years (HR: 2.66, 95% CI: 2.14-3.31, P<0.001), age>60 years (HR: 3.52, 95% CI: 2.83-4.37, P<0.001), non-Hispanic ethnicity (HR: 1.41, 95% CI: 1.26-1.58, P<0.001), and non-urban residency (HR: 1.10, 95% CI: 1.01-1.21, P=0.027) were also identified as risk factors for the development of SPMs. Conversely, female (HR: 0.80, 95% CI: 0.75-0.85, P<0.001), primary lymph node involvement (HR: 0.90, 95% CI: 0.82-0.98, P=0.019), and Grade 3 (HR: 0.89, 95% CI: 0.81-0.97, P=0.007) were associated with a lower risk of developing SPMs (Figure 1).

Figure 1 Cumulative incidence of Second Primary Malignancies in patients with FL diagnosed between 2000 and 2020. TTT: time to treatment. Others of the race: American Indian/AK Native, Asian/Pacific Islander. Others of martial status: Divorced, Separated, Widowed, Unknown, Unmarried or Domestic Partner.

Multivariate competing-risk regression found that patients aged 40-60 years (HR: 2.59, 95% CI: 2.07-3.23, P<0.001), >60 years (HR: 3.48, 95% CI: 2.79-4.33, P<0.001), non-Hispanic ethnicity (HR: 1.30, 95% CI: 1.16-1.46, P<0.001), and initial treatment with radiotherapy or surgery (HR: 1.12, 95% CI: 1.03-1.22, P=0.007; HR: 1.07, 95% CI: 1.00-1.14, P=0.037) were associated with an increased risk of SPMs. In contrast, female patients (HR: 0.79, 95% CI: 0.74-0.85, P<0.001), single status (HR: 0.89,

95% CI: 0.80-0.99, P=0.029) and those with Grade 3 (HR: 0.89, 95% CI: 0.82-0.97, P=0.011) had a reduced risk of developing SPMs (Figure 2).

Figure 2 Forest plot of multivariate competing risks regression analysis for risks of second primary malignancies in follicular lymphoma patients. Others of the race: American Indian/AK Native, Asian/Pacific Islander. Others of martial status: Divorced, Separated, Widowed, Unknown, Unmarried or Domestic Partner.

3.4 Survival Outcomes

In our cohort, 11,562 FL patients (34.9%) died, with a crude mortality rate of 0.045 per person per year. The 5-year overall survival (OS) and lymphoma-specific survival (LSS) rates were 81.1% and 88.0%, respectively. The median OS was 192 months, while the median LSS was not reached. In the SPMs group, 2,053 patients died (2,053/3,822, 53.7%), compared to 9,509 deaths (9,509/29,282, 32.5%) in the non-SPM group, indicating a significantly higher mortality rate in the SPMs group (P<0.001). The 5-year OS rates for the SPMs and non-SPMs groups were 83.9% and 80.7%, with median OS of 150 months and 210 months, respectively (P<0.001). The 5-year LSS rates were 93.9% for the SPM group and 87.2% for the non-SPM group, with median LSS of 116 and 76 months, respectively (P<0.001). Mortality risk in the SPMs group increased significantly 91 months after FL diagnosis, and lymphoma-specific death risk increased 175 months after diagnosis (Figure 3).

Figure 3 Kaplan–Meier analysis overall survival. (A) and Lymphoma-specific survival (B) by Log-Rank test and overall survival (C) and Lymphoma-specific survival (D) by Landmark test of follicular lymphoma patients with or without the secondary primary malignancies.

Further analysis of causes of death among FL patients revealed that 5,732 patients (49.6%) died from lymphoma, 5,721 patients (49.5%) died from non-malignant diseases, and 109 patients (0.9%) died from other cancers. The top five causes of death were lymphoma, heart disease, Chronic Obstructive Pulmonary Disease (COPD), cerebrovascular disease, and lung infection. Patients in the SPMs group had a significantly higher rate of death from other cancers compared to the non-SPMs group (5.3% vs. 0.0%, P<0.001), making other cancers the fifth leading cause of death in the SPM group. Among non-malignant causes of death, the SPMs group had a lower proportion of deaths due to heart disease and COPD compared to the non-SPMs group (7.7% vs. 16.6%, P<0.001; 2.9% vs. 3.2%, P<0.001) (Table 2).

Table 2 Causes of death in patients with follicular lymphoma

**Death cause** (N, %)	**All deaths** (N = 11,562)	**non-SPMs** (N = 9,509)	**SPMs** (N = 2,053)	***p* value**
Lymphoma	5,732 (49.6%)	5,060(53.2%)	672(32.7%)	0.726
Another Malignancy	109 (0.9%)	1(0.0%)	108(5.3%)	**<0.001**
Non-cancer causes	5,721 (49.5%)	4,448(46.8%)	1,273(62.0%)	**<0.001**
Diseases of Heart	1,737 (15.0%)	1,578(16.6%)	159(7.7%)	**0.001**
COPD	363 (3.1%)	304(3.2%)	59(2.9%)	**0.003**

Cerebrovascular Diseases	301 (2.6%)	275(2.9%)	26(1.3%)	0.106
Pneumonia and Influenza	245 (2.1%)	219(2.3%)	26(1.3%)	0.627
Alzheimer's Disease	220 (1.9%)	201(2.1%)	19(0.9%)	0.168
Septicemia	117 (1.0%)	99(1.0%)	18(0.9%)	0.201
Other Non-cancer causes	2,738(23.7%)	1,772(18.6%)	966(47.1%)	**<0.001**

* χ^2 test was used for comparison. Significant values (P <0 .05) are highlighted in bold.

COPD: chronic obstructive pulmonary disease

3.5 Survival Analysis

A multivariate analysis of FL patients found that age≥40 years, Black race, single status, non-urban residency, primary lymphoma site within lymph nodes, Grade 3, advanced Ann Arbor stage, and B symptoms were independent risk factors for both OS and LSS. Additionally, FL patients who received chemotherapy as their initial treatment had a worse LSS. Female, higher income, diagnosis after 2005, diagnosis-to-treatment time (DTT) over one month, and radiotherapy or surgery were linked to reduced risks of both OS and LSS. The development of SPMs was associated with a decreased risk of LSS in FL patients (Table 3).

Table 3 Multivariate analysis of prognostic factors in patients with follicular lymphoma after initial diagnosis

**Characteristics**	**Overall survival***		**Lymphoma-specific overall survival†**	
	**HR (95% CI)**	**P**	**HR (95% CI)**	**P**
**Sex**				
Male	1	-	1	-
Female	0.73 (0.71-0.76)	**<0.001**	0.76 (0.72-0.81)	**<0.001**
**Age**				
15-39	1	-	1	-

40-60	1.96 (1.72-2.25)	**<0.001**	1.66 (1.40-1.96)	**<0.001**
>60	6.92 (6.06-7.90)	**<0.001**	3.98 (3.37-4.69)	**<0.001**
**Race**				
White	1	-	1	-
Black	1.13 (1.04-1.23)	**0.006**	1.21 (1.07-1.36)	**0.002**
Others	0.93 (0.85-1.02)	0.131	1.06 (0.94-1.20)	0.32
**Ethnicity**				
Hispanics	1	-	-	-
Non-Hispanics	1.00 (0.94-1.07)	0.961	-	**-**
**Martial status**				
Married	1	-	1	-
Single	1.41 (1.33-1.50)	**<0.001**	1.30 (1.19-1.41）	**<0.001**
other	1.73 (1.66-1.80)	**<0.001**	1.50 (1.41-1.59)	**<0.001**
**Income**				
<$65,000	1	-	1	-
$65,000 - $74,999	0.97 (0.92-1.02)	0.244	0.99 (0.92-1.07)	0.87
≥$75,000	0.83 (0.79-0.87)	**<0.001**	0.86 (0.80-0.93)	**<0.001**
**Rural-Ubran**				
metropolitan areas	1	-	1	-
Nonmetropolitan counties	1.07 (1.01-1.13)	**0.026**	1.10 (1.01-1.20)	**0.022**
**Year of diagnosis**				
2000-2004	1	-	1	-
2005-2009	0.80 (0.77-0.84)	**<0.001**	0.67 (0.63-0.71)	**<0.001**
2010-2014	0.72 (0.65-0.80)	**<0.001**	0.58 (0.50-0.66)	**<0.001**
2015-2019	0.65 (0.57-0.74)	**<0.001**	0.55 (0.46-0.65)	**<0.001**
2020	0.69 (0.48-0.98)	**0.040**	0.53 (0.33-0.85)	**0.009**
**Site**				
NHL – Extranodal	1	-	1	-
NHL – Nodal	1.11 (1.05-1.18)	**<0.001**	1.26 (1.15-1.38)	**<0.001**
**FL-subtype**				
Grade1-2	1	-	1	-
Grade3	1.08 (1.03-1.14)	**0.002**	1.09 (1.02-1.17)	**0.017**
Grade NOS	1.19 (1.14-1.24)	**<0.001**	1.25 (1.18-1.33)	**<0.001**
**Ann Arbor stage**				
I/ II	1	-	1	-
III/IV	1.28 (1.23-1.34)	**<0.001**	1.57 (1.47-1.68)	**<0.001**
Unknown	1.10 (1.02-1.19)	**0.018**	1.09 (0.97-1.22)	0.15
**B symptom**				
None	1	-	1	-
Any	1.41 (1.29-1.54)	**<0.001**	1.57 (1.39-1.77)	**<0.001**

Unknown	1.28 (1.15-1.42)	**<0.001**	1.51 (1.31-1.74)	**<0.001**
**Diagnosis-to-treatment time**				
≤1month	1	-	1	-
＞1month	0.80 (0.76-0.84)	**<0.001**	0.72 (0.66-0.77)	**<0.001**
**Radiotherapy**				
Not received	1	-	1	-
Received	0.86 (0.82-0.91)	**<0.001**	0.87 (0.80-0.95)	**0.001**
**Chemotherapy**				
Not received	1	-	1	-
Received	1.02 (0.97-1.07)	0.408	1.24 (1.16-1.33)	**<0.001**
**Surgery**				
Not received	1	-	1	-
Received	0.84 (0.80-0.87)	**<0.001**	0.81 (0.76-0.87)	**<0.001**
**SPMs**				
No	1	-	1	-
Yes	1.03 (0.98-1.08)	0.181	0.65 (0.60-0.71)	**<0.001**

* Cox hazards proportional analysis of prognostic factors for patients with follicular lymphoma. Significant values (P <0 .05) are highlighted in bold.

† Competing-risk regression analysis of prognostic factors for patients died from cancer, Including lymphomas. Non-cancer causes of death as competing events. Significant values (P <0 .05) are highlighted in bold.

Survival analysis indicated that the development of SPMs influences the long-term prognosis of FL patients. A multivariate analysis revealed that FL patients over 60 years, those who were single, and those diagnosed after 2010 faced a higher risk of OS after developing SPMs. However, surgery was associated with a reduced risk of OS in these patients. Additionally, initial radiotherapy reduced both OS (HR: 0.87, 95% CI: 0.77-0.97, P=0.015) and LSS (HR: 0.80, 95% CI: 0.65-0.99, P=0.037) among patients who developed SPMs (Table 4).

Table 4 Multivariate analysis of prognostic factors in patients with follicular lymphoma after

SPMs（N=3844）

Characteristics	Overall survival[*]		Lymphoma-specific overall survival[†]	
	**HR (95% CI)**	**P**	**HR (95% CI)**	**P**
**Age**				
15-39	1	-	-	-
40-60	1.25 (0.87-1.78)	0.227	-	-
>60	2.75 (1.93-3.92)	**<0.001**	-	-
**Martial status**				
Married	1	-	1	-
Single	1.28 (1.10-1.48)	**0.002**	1.14 (0.89-1.47)	0.29
other	1.39 (1.26-1.54)	**<0.001**	1.25 (1.05-1.50)	**0.014**
**Income**				
<$65,000	1	-	-	-
$65,000 - $74,999	0.99 (0.88-1.12)	0.935	-	-
≥$75,000	0.84 (0.75-0.94)	**0.003**	-	-
**Rural-Ubran**				
metropolitan areas	1	-	-	-
Nonmetropolitan counties	1.10 (0.96-1.26)	0.162	-	-
**Year of diagnosis**				
2000-2009	1	-	-	-
2010-2020	1.75 (1.36-2.25)	**<0.001**	-	-
**Ann Arbor stage**				
I/ II	1	-	1	-
III/IV	1.09 (0.99-1.20)	0.094	1.15 (0.97-1.37)	0.11
Unknown	1.21 (1.02-1.42)	0.031	0.94 (0.68-1.30)	0.71
**B symptom**				
None	1	-	-	-
Any	1.01 (0.79-1.30)	0.920	-	-
Unknown	1.19 (0.91-1.56)	0.198	-	-
**Radiotherapy**				
Not received	1	-	1	-
Received	0.87 (0.77-0.97)	**0.015**	0.80 (0.65-0.99)	**0.037**
**Chemotherapy**				
Not received	1	-	1	-
Received	1.03 (0.92-1.14)	0.608	1.13 (0.95-1.35)	0.17
**Surgery**				
Not received	1	-	-	-

Received	0.89 (0.81-0.99)	**0.025**	-	-

* Cox hazards proportional analysis of prognostic factors for patients with follicular lymphoma. Significant values (P <0 .05) are highlighted in bold.

† Competing-risk regression analysis of prognostic factors for patients died from cancer, Including lymphomas. Non-cancer causes of death as competing events. Significant values (P <0 .05) are highlighted in bold.

4.Discussion

Follicular lymphoma (FL) is a typically indolent lymphoma originating from germinal center B cells. Advances in treatment have significantly improved FL patient survival[12]. In the era of rituximab therapy, patients diagnosed with FL generally have a promising prognosis. Recent data from French and U.S. cohorts show 10-year OS rates of FL patients were 79.8% and 76.6%, respectively[13], although patients may still experience late effects following cancer treatment, impacting their quality of life.

Previous studies have reported that the APC for FL in Taiwan from 2002 to 2019 was 3.49%, in Japan from 2001 to 2008 was 12.66%, and from 2014 to 2019 was 4.95%. In South Korea, the change was 5.72% from 2001 to 2012, and 7.93% from 2015 to 2022[14]. In contrast, the incidence rate among the Whites decreased annually by -2.1% from 2008 to 2017, dropping from 3.42 cases in 2008 to 2.74 cases per 100,000 person-years in 2017, a decrease of 20%[15]. Between 2000 and 2014 within the Polish population, no significant trend was noted in the standardized overall

incidence rates of FL[16]. Consistent with the findings of the above studies, our research shows an APC of -1.6% for FL in the U.S. population from 2000 to 2020. This suggests that greater attention to FL in Asian countries is needed, along with etiological studies that focus especially on modifiable risk factors, to understand and address the factors driving the rising incidence of FL group.

Among 33,104 FL patients in our study, the median age at diagnosis was 62 years, with a balanced male-to-female ratio, matching typical FL incidence patterns[1,17]. Consistent with prior research, male and older patients had a higher incidence of SPMs[18]. Notably, a higher proportion of patients with skin as the primary site developed SPMs compared to those without such a history. While the existing literature on this is limited, we hypothesize that this may be related to the immunosuppressive therapies, such as radiation therapy (a common treatment for primary cutaneous follicular lymphoma) and chemotherapy[19]. In our study, the SPM subtypes significantly elevated in FL patients are those thought to be linked to immune dysfunction and the use of chemotherapeutic agents, including acute myeloid leukemia (AML) and malignancies of the lung and bronchus, skin[6]. It has been reported that adjusting for FLIPI, B-symptoms, and treatment lines, revealed three or more treatment lines as a significant independent risk factor for developing secondary hematological malignancies (odds ratio: 5.0, 95% CI: 1.2–20.7, P = 0.026)[11]. Variations

in our results compared to it may stem from population heterogeneity, with data derived from Finnish and Spanish cohorts. Our study specifically focused on SPM as the outcome, and the analysis did not account for competing risks. Giri's group extracted data from 1992 to 2011 from the SEER database, encompassing 15,517 cases, and conducted a multivariate analysis using the Fine-Gray regression model. This analysis identified age> 65 years, male gender, and history of radiation therapy as independent predictors of an increased cumulative incidence of SPMs[5]. Conversely, no significant differences in SPM incidence were observed across diagnosis years, FL grade, or between black and white racial groups. Our investigation concurs with the Giri group's report, affirming advanced age, male gender, and radiotherapy as pivotal risk factors for SPMs. Our analysis further identifies non-Hispanic ethnicity and surgical procedures as novel risk factors, with single marital status and Grade 3 disease conferring protection. These variations likely reflect temporal shifts and advancements in therapeutic approaches.

RT is a powerful treatment that can effectively destroy cancer but also poses risks to healthy tissue. When radiation doses exceed the repair capacity of surrounding normal tissues, irreversible damage may occur[20]. RT can trigger immunosuppressive responses, including the polarization of macrophages towards an M2 phenotype[21], the shift of neutrophils to an N2 phenotype[22], and the accumulation of myeloid-derived suppressor

cells[23]. These immune cells typically exert their immunosuppressive influence through the upregulation of PD-L1 and through various other mechanisms[24].

Thus, Clinical practitioners should be alerted to the importance of screening for SPMs in male and elderly patients. And early screening with low-dose CT may improve early diagnosis of lung cancer and the survival rate in this group[25]. Clinicians should carefully select patients and radiation techniques to minimize radiation-induced cancers, and close follow-up can enable early detection and improve treatment outcomes. Furthermore, researchers should further investigate the mechanisms linking immunosuppressive therapies to the elevated risk of SPMs in FL patients, as a deeper understanding of these relationships could help inform therapeutic decision-making and preventive strategies.

The survival analysis found that FL patients with SPMs did not show a decrease in survival within the first 91 months compared to those without SPMs, with LSS increasing only after 175 months. This could be attributed to the early identification of high-risk patients in lymphoma, targeted therapies, cellular treatments, and other interventions have demonstrated promising efficacy[26,27].

Only a limited number of studies have previously investigated the risk factors associated with FL[5,28,29]. In concordance with prior studies, multivariate analysis reveals the Ann Arbor stage as a significant predictor

of OS and LSS in FL patients, emphasizing the critical role of early diagnosis, indicating the importance of early detection[30,31]. Moreover, the data demonstrated a decline in patient survival with older ages. This condition may be associated with immunological insufficiency and somatic decline in elderly people. Studies have revealed that individuals with B symptoms have a poor prognosis, emphasizing the need for careful monitoring of high-risk patients[32].

In terms of treatment impact on survival, initial chemotherapy increased LSS, while radiotherapy or surgery was associated with reduced mortality risks in FL patients. For patients who developed SPMs, surgery was associated with a decreased risk of OS and initial radiotherapy lowered both OS and LSS. Therefore, treatment selection should be approached with caution, considering these survival impacts.

Our study has limitations: it is a retrospective study, which may be subject to selection and information bias. The data from the SEER database may not fully represent the global epidemiological landscape. Additionally, the SEER database lacks some crucial clinical variables, such as specific radiation and chemotherapy regimens, surgical methods, fusion genes, imaging details, and FLIPI scores, which are closely associated with the prognosis of FL patients. Future studies should use prospective data to validate and expand these findings.

In conclusion, using the largest FL cohort to date, our study meticulously describes the epidemiological characteristics of FL. After excluding the potential for transformation to more aggressive subtypes and the competing risk of death, the study identifies the risk factors contributing to the development of SPMs in FL patients and delves into the causes of death among patients. Additionally, we analyzed the risk factors affecting the survival rates of FL patients, as well as those patients with SPMs. These findings offer clinicians valuable tools for earlier identification of high-risk patients, enabling more intensive monitoring and evaluation.

Acknowledgements

The authors thank all the study patients, investigators, and coordinators.

Reference

1. Carbone A, Roulland S, Gloghini A, Younes A, von Keudell G, López-Guillermo A, et al. Follicular lymphoma. Nature reviews Disease primers. 2019;5(1):83. Epub 2019/12/14. doi: 10.1038/s41572-019-0132-x.
2. Jacobsen E. Follicular lymphoma: 2023 update on diagnosis and management. American journal of hematology. 2022;97(12):1638-51. Epub 2022/10/19. doi: 10.1002/ajh.26737.

3. Hiddemann W, Kneba M, Dreyling M, Schmitz N, Lengfelder E, Schmits R, et al. Frontline therapy with rituximab added to the combination of cyclophosphamide, doxorubicin, vincristine, and prednisone (CHOP) significantly improves the outcome for patients with advanced-stage follicular lymphoma compared with therapy with CHOP alone: results of a prospective randomized study of the German Low-Grade Lymphoma Study Group. Blood. 2005;106(12):3725-32. Epub 2005/08/27. doi: 10.1182/blood-2005-01-0016.

4. Salles G, Mounier N, de Guibert S, Morschhauser F, Doyen C, Rossi JF, et al. Rituximab combined with chemotherapy and interferon in follicular lymphoma patients: results of the GELA-GOELAMS FL2000 study. Blood. 2008;112(13):4824-31. Epub 2008/09/19. doi: 10.1182/blood-2008-04-153189.

5. Giri S, Bhatt VR, Verma V, Pathak R, Bociek RG, Vose JM, et al. Risk of Second Primary Malignancies in Patients With Follicular Lymphoma: A United States Population-based Study. Clinical lymphoma, myeloma & leukemia. 2017;17(9):569-74. Epub 2017/07/16. doi: 10.1016/j.clml.2017.06.028.

6. Dinnessen MAW, Visser O, Tonino SH, Posthuma EFM, Blijlevens NMA, Kersten MJ, et al. Risk of second primary malignancies in patients with follicular lymphoma: a population-based study in the

Netherlands, 1989-2018. Blood cancer journal. 2021;11(11):179. Epub 2021/11/15. doi: 10.1038/s41408-021-00574-5.

7. Watanabe T, Tobinai K, Wakabayashi M, Morishima Y, Kobayashi H, Kinoshita T, et al. Outcomes after R-CHOP in patients with newly diagnosed advanced follicular lymphoma: a 10-year follow-up analysis of the JCOG0203 trial. The Lancet Haematology. 2018;5(11):e520-e31. Epub 2018/11/06. doi: 10.1016/s2352-3026(18)30155-8.

8. Hiddemann W, Barbui AM, Canales MA, Cannell PK, Collins GP, Dürig J, et al. Immunochemotherapy With Obinutuzumab or Rituximab for Previously Untreated Follicular Lymphoma in the GALLIUM Study: Influence of Chemotherapy on Efficacy and Safety. Journal of clinical oncology : official journal of the American Society of Clinical Oncology. 2018;36(23):2395-404. Epub 2018/06/02. doi: 10.1200/jco.2017.76.8960.

9. Guadagnolo BA, Li S, Neuberg D, Ng A, Hua L, Silver B, et al. Long-term outcome and mortality trends in early-stage, Grade 1-2 follicular lymphoma treated with radiation therapy. International journal of radiation oncology, biology, physics. 2006;64(3):928-34. Epub 2005/10/26. doi: 10.1016/j.ijrobp.2005.08.010.

10. Barzenje DA, Cvancarova Småstuen M, Liestøl K, Fosså A, Delabie J, Kolstad A, et al. Radiotherapy Compared to Other Strategies in the

Treatment of Stage I/II Follicular Lymphoma: A Study of 404 Patients with a Median Follow-Up of 15 Years. PloS one. 2015;10(7):e0131158. Epub 2015/07/07. doi: 10.1371/journal.pone.0131158.

11. Prusila REI, Sorigue M, Jauhiainen J, Mercadal S, Postila A, Salmi P, et al. Risk of secondary haematological malignancies in patients with follicular lymphoma: an analysis of 1028 patients treated in the rituximab era. British journal of haematology. 2019;187(3):364-71. Epub 2019/07/04. doi: 10.1111/bjh.16090.

12. Zinzani PL, Muñoz J, Trotman J. Current and future therapies for follicular lymphoma. Experimental hematology & oncology. 2024;13(1):87. Epub 2024/08/23. doi: 10.1186/s40164-024-00551-1.

13. Sarkozy C, Maurer MJ, Link BK, Ghesquieres H, Nicolas E, Thompson CA, et al. Cause of Death in Follicular Lymphoma in the First Decade of the Rituximab Era: A Pooled Analysis of French and US Cohorts. Journal of clinical oncology : official journal of the American Society of Clinical Oncology. 2019;37(2):144-52. Epub 2018/11/28. doi: 10.1200/jco.18.00400.

14. Chiu LC, Lin CW, Li HJ, Chen JH, Chuang FC, Lin SF, et al. Incidence Trend of Follicular Lymphoma in Taiwan Compared to Japan and Korea, 2001-2019. Journal of clinical medicine. 2023;12(4). Epub 2023/02/26. doi: 10.3390/jcm12041417.

15. Su YC, Chiu BC, Li HJ, Yang WC, Chen TY, Yeh SP, et al. Investigation of the incidence trend of follicular lymphoma from 2008 to 2017 in Taiwan and the United States using population-based data. PloS one. 2022;17(3):e0265543. Epub 2022/03/18. doi: 10.1371/journal.pone.0265543.
16. Szumera-Ciećkiewicz A, Wojciechowska U, Didkowska J, Poleszczuk J, Rymkiewicz G, Paszkiewicz-Kozik E, et al. Population-based epidemiological data of follicular lymphoma in Poland: 15 years of observation. Scientific reports. 2020;10(1):14610. Epub 2020/09/05. doi: 10.1038/s41598-020-71579-6.
17. Dada R. Diagnosis and management of follicular lymphoma: A comprehensive review. European journal of haematology. 2019;103(3):152-63. Epub 2019/07/05. doi: 10.1111/ejh.13271.
18. Liu Y, Chu Y, Liu J, Ge X, Ding M, Li P, et al. Incidence and mortality of second primary malignancies after lymphoma: a population-based analysis. Annals of medicine. 2023;55(2):2282652. Epub 2023/11/27. doi: 10.1080/07853890.2023.2282652.
19. Nikolaou V, Koumprentziotis IA, Papadavid E, Patsatsi A, Diavati S, Tsimpidakis A, et al. Clinical features, treatment options and outcomes in primary cutaneous B-cell lymphomas: a real-world, multicenter, retrospective study. International journal of dermatology. 2024. Epub 2024/11/13. doi: 10.1111/ijd.17564.

20. Guo S, Yao Y, Tang Y, Xin Z, Wu D, Ni C, et al. Radiation-induced tumor immune microenvironments and potential targets for combination therapy. Signal transduction and targeted therapy. 2023;8(1):205. Epub 2023/05/20. doi: 10.1038/s41392-023-01462-z.
21. Genard G, Lucas S, Michiels C. Reprogramming of Tumor-Associated Macrophages with Anticancer Therapies: Radiotherapy versus Chemo- and Immunotherapies. Frontiers in immunology. 2017;8:828. Epub 2017/08/05. doi: 10.3389/fimmu.2017.00828.
22. Boivin G, Ancey PB, Vuillefroy de Silly R, Kalambaden P, Contat C, Petit B, et al. Anti-Ly6G binding and trafficking mediate positive neutrophil selection to unleash the anti-tumor efficacy of radiation therapy. Oncoimmunology. 2021;10(1):1876597. Epub 2021/02/26. doi: 10.1080/2162402x.2021.1876597.
23. Liang H, Deng L, Hou Y, Meng X, Huang X, Rao E, et al. Host STING-dependent MDSC mobilization drives extrinsic radiation resistance. Nature communications. 2017;8(1):1736. Epub 2017/11/25. doi: 10.1038/s41467-017-01566-5.
24. Wang NH, Lei Z, Yang HN, Tang Z, Yang MQ, Wang Y, et al. Radiation-induced PD-L1 expression in tumor and its microenvironment facilitates cancer-immune escape: a narrative review. Annals of translational medicine. 2022;10(24):1406. Epub 2023/01/21. doi: 10.21037/atm-22-6049.

25. Oudkerk M, Liu S, Heuvelmans MA, Walter JE, Field JK. Lung cancer LDCT screening and mortality reduction - evidence, pitfalls and future perspectives. Nature reviews Clinical oncology. 2021;18(3):135-51. Epub 2020/10/14. doi: 10.1038/s41571-020-00432-6.
26. Sant M, Vener C, Lillini R, Rossi S, Bonfarnuzzo S, Marcos-Gragera R, et al. Long-term survival for lymphoid neoplasms and national health expenditure (EUROCARE-6): a retrospective, population-based study. The Lancet Oncology. 2024;25(6):731-43. Epub 2024/05/05. doi: 10.1016/s1470-2045(24)00141-4.
27. Batlevi CL, Sha F, Alperovich A, Ni A, Smith K, Ying Z, et al. Follicular lymphoma in the modern era: survival, treatment outcomes, and identification of high-risk subgroups. Blood cancer journal. 2020;10(7):74. Epub 2020/07/18. doi: 10.1038/s41408-020-00340-z.
28. Morton LM, Curtis RE, Linet MS, Bluhm EC, Tucker MA, Caporaso N, et al. Second malignancy risks after non-Hodgkin's lymphoma and chronic lymphocytic leukemia: differences by lymphoma subtype. Journal of clinical oncology : official journal of the American Society of Clinical Oncology. 2010;28(33):4935-44. Epub 2010/10/14. doi: 10.1200/jco.2010.29.1112.
29. Zheng W, Wang S, Liang Y, Ning H. Is radiotherapy still the optimal initial choice for patients with early-stage low-grade follicular

lymphoma in the modern era? A population-based study. Annals of hematology. 2024;103(11):4589-98. Epub 2024/09/28 22:44. doi: 10.1007/s00277-024-06022-1.

30. Guevara DL, Bernard S, Manhood S, Melani S, Yerovi F, Rodríguez M. [Prognostic value of interim PET/CT in non-hodgkin lymphoma]. Revista medica de Chile. 2020;148(11):1558-67. Epub 2021/04/13. doi: 10.4067/s0034-98872020001101558..

31. Li X, Yang Z, Li J, Wang G, Sun A, Wang Y, et al. The development of a prediction model based on random survival forest for the prognosis of non- Hodgkin lymphoma: A prospective cohort study in China. Heliyon. 2024;10(12):e32788. Epub 2024/07/18. doi: 10.1016/j.heliyon.2024.e32788.

32. Iqbal M, Jiang L, Li KD, Moustafa MA, Kimbrough EO, Ansell SM, et al. Poly-lymphomatous Syndrome With Concurrent or Sequential Hodgkin and Non-Hodgkin lymphoma. Clinical lymphoma, myeloma & leukemia. 2023;23(2):138-44. Epub 2022/12/13. doi: 10.1016/j.clml.2022.11.004.

Supporting information

S1 Fig. Prevalence of follicular lymphoma. (A)Prevalence of follicular lymphoma by gender (B)Prevalence of follicular lymphoma by age (C) per 10,000: Surveillance, Epidemiology, and End Results (SEER) 2000–2020.

S1 Table. Follicular Lymphoma prevalence with different characteristics per 100,000: Surveillance, Epidemiology, and End Results (SEER) 2000–2020.

S2 Table. Follicular Lymphoma Treatment Modalities.

S3 Table. Primary locations of patients with follicular lymphoma at diagnosis.

S4 Table. Second Primary Malignancies in Follicular Lymphoma.

Data Availability Statement:

All relevant data are within the paper and its Supporting Information files

Funding:

This study was supported by Sichuan Science and Technology Department for Key Research and Development Program (2019YFS0027).The funders had no role in study design, data collection and analysis, decision to publish, or preparation of the manuscript.

Author Contributions:

Conceptualization: Caigang Xu.

Data curation: Ying Tian, Wanxi Yang.

Methodology: Yuanxiao Li, Wenjiao Tang.

Resources: Juan Xu, Wenjiao Tang, Caigang Xu.

Software: Ying Tian.

Supervision: Juan Xu, Caigang Xu.

Writing – original draft: Ying Tian, Wanxi Yang, Juan Xu, Yuanxiao Li.

Writing – review & editing: Caigang Xu

Competing interests:

The authors have declared that no competing interests exist.

Figure 1

Click here to access/download;Figure;Fig1.tif

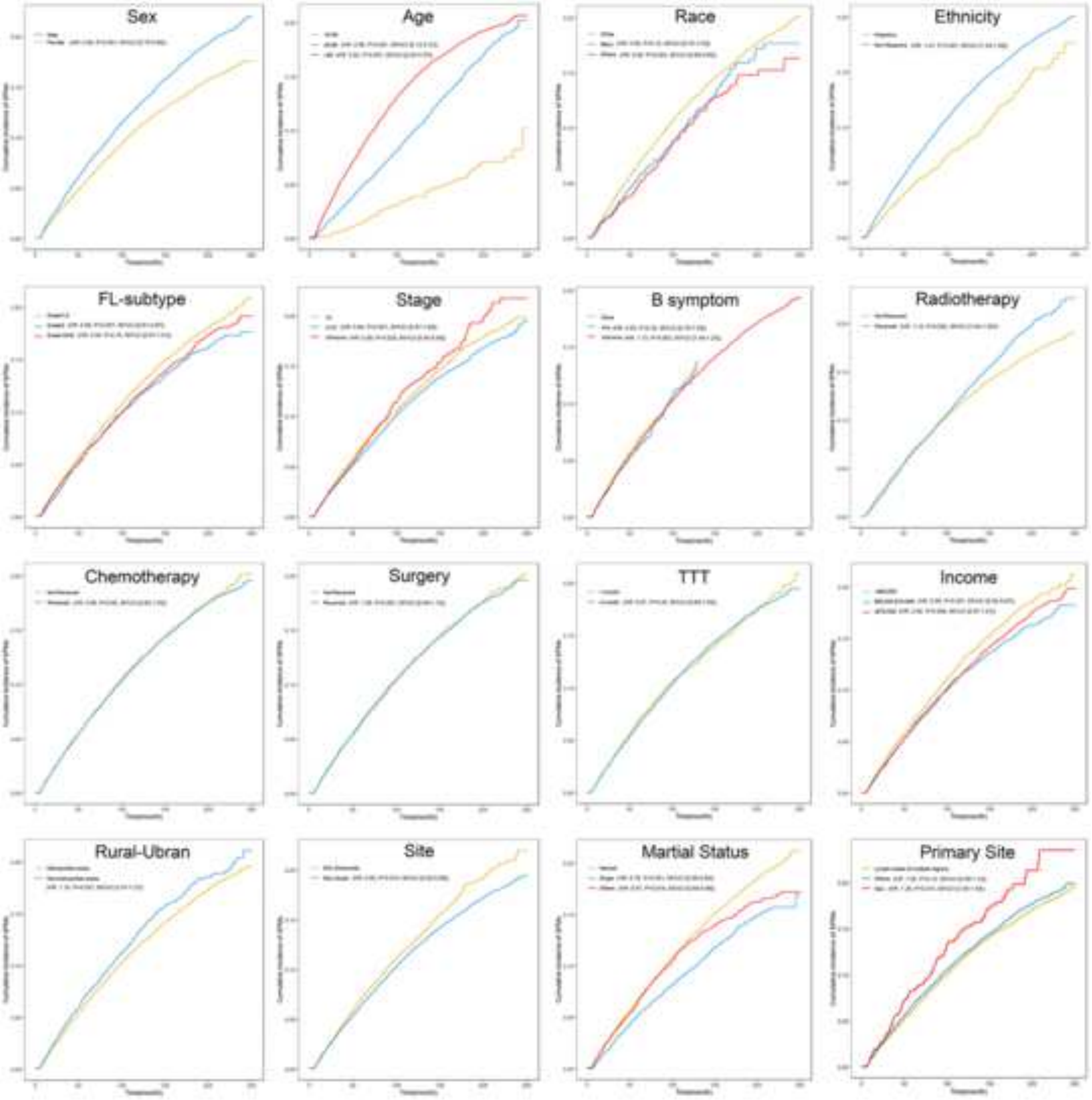

Figure 2 Click here to access/download;Figure;Fig2.tif

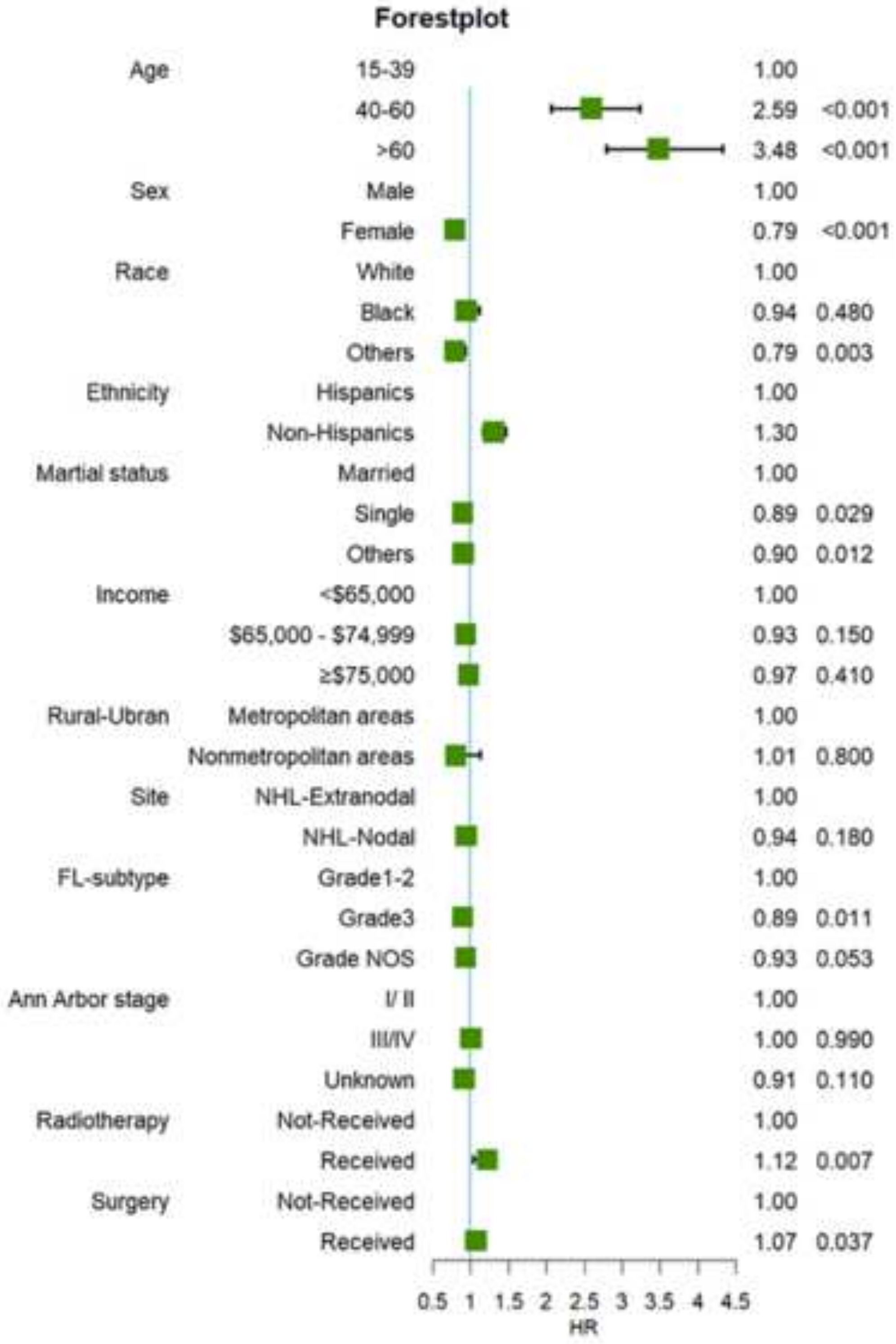

Figure 3 Click here to access/download;Figure;Fig3.tif

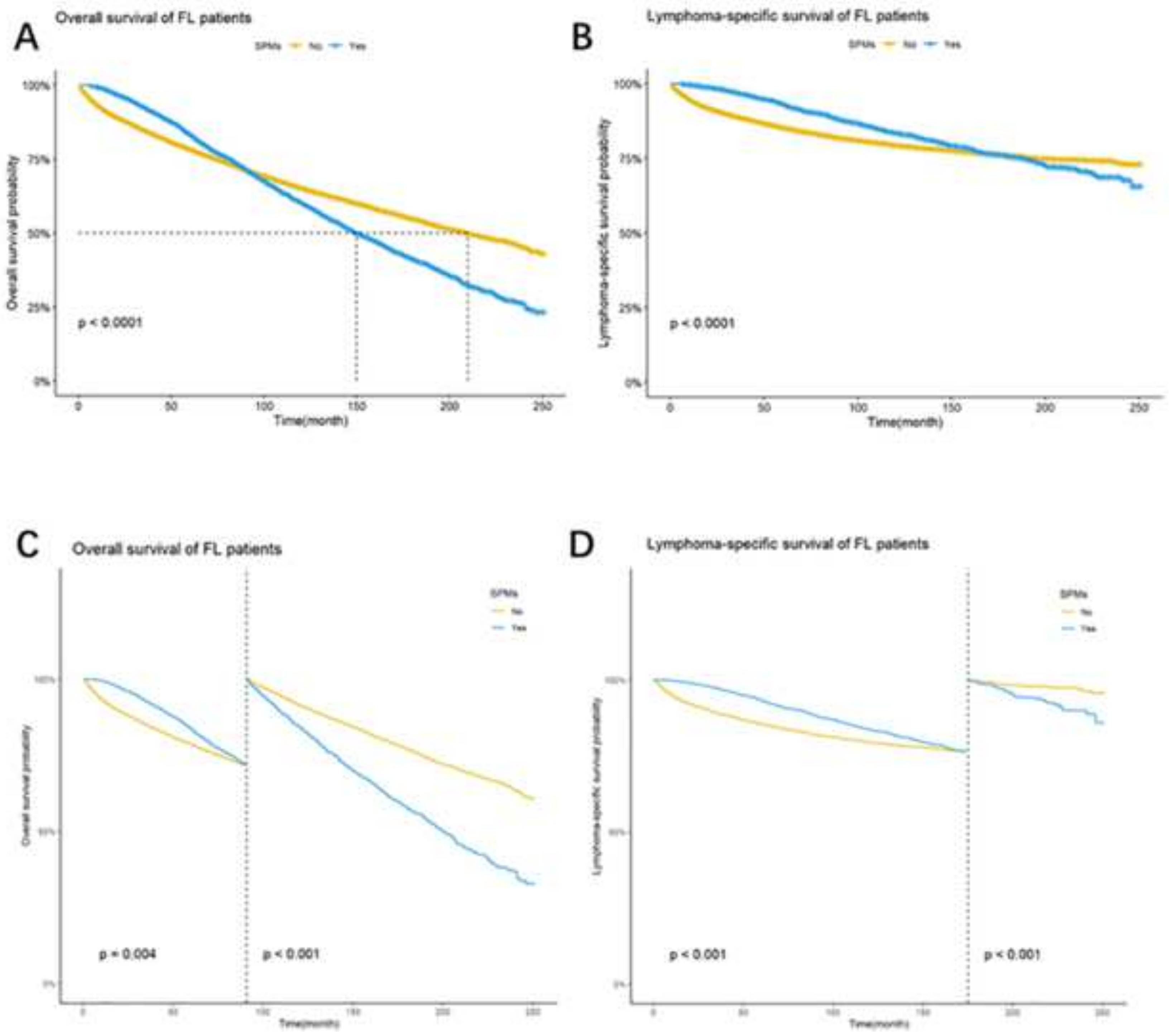

Click here to access/download
Supporting Information
Supplementary Data-FL.docx
