# Supplementary material for: Risk and prognosis of second primary malignancies in patients with follicular lymphoma in the era of rituximab: A population study based on the SEER database
Source: PLoS One. 2025 May 28;20(5):e0324532. doi: 10.1371/journal.pone.0324532 (PMC12118830; doi:10.1371/journal.pone.0324532)
Supplement: Attachment — Submitted filename: Response to reviewers.docx [file pone.0324532.s021.docx]

Dear Editor and Esteemed Reviewers,

We sincerely appreciate your valuable insights and constructive suggestions on our manuscript. All raised concerns have been systematically addressed through the following point-by-point responses: each query has been bolded and numbered sequentially for efficient navigation, critical revisions are highlighted in red font for instant visual identification.

Should any clarification or additional information be required, please contact us at xucaigang@wchscu.cn. We remain fully available for further modifications as needed.

We are profoundly grateful for your expert guidance in enhancing the scientific rigor of this work, and respectfully await your further instruction.

Sincerely,

Caigang Xu

Corresponding Author

**Additional Editor Comments:**

**1.Line 50 Introduction: It would benefit from a more detailed discussion of the current gaps in the literature, particularly regarding the impact of rituximab on SPM risk. The authors should also clarify why their study is necessary and how it advances the field beyond previous studies.**

We sincerely appreciate the editor's insightful comments. In accordance with the suggestions, we have substantially revised the Introduction section, with key modifications highlighted in red to explicitly address the editorial concerns and enhance the clarity of our responses. These revisions have been consistently incorporated throughout the manuscript to ensure uniformity with the updated content.

**Introduction**

Follicular lymphoma (FL), originating from the germinal center B cells in lymph nodes or lymphoid tissue, is one of the most common indolent forms of non-Hodgkin's lymphoma (NHL). In Western countries, FL accounts for about 20-35% of NHL cases[1, 2], with an age-standardized incidence of 2–4 per 100,000 person-years[1]. The therapeutic landscape for FL has been revolutionized by anti-CD20 monoclonal antibodies, particularly rituximab-based immunochemotherapy, which has elevated 10-year overall survival (OS) rates beyond 80%[2]. While these advancements have substantially improved survival outcomes, emerging survivorship challenges including long-term treatment toxicities and second primary malignancies (SPMs) demand increased clinical attention. Notably, contemporary studies indicate that SPM incidence remains unaffected by the incorporation of rituximab into therapeutic regimens[3-6], emphasizing the critical need for optimized surveillance strategies and preventive measures in the current era of rituximab-dominated therapies.

Previous studies have showed that FL patients face a higher SPMs risk than the general population, with factors such as older age, male, chemotherapy, radiotherapy, radioimmunotherapy, autologous stem cell transplantation following high-dose chemotherapy, multiple treatments, and B symptoms linked to SPM development[2, 5, 7-10]. However, existing research presents conflicting conclusions regarding risk factor associations, compounded by methodological limitations: (1) most analysis predate widespread rituximab adoption, potentially obscuring modern risk profiles shaped by newer combination therapies (e.g., rituximab plus lenalidomide) [11]; (2) conventional Kaplan-Meier analysis overestimate SPM incidence by neglecting competing mortality risks; and (3) the clinical characteristics, survival outcomes, and prognostic determinants for FL patients post-SPM diagnosis remain poorly characterized due to SPM rarity and insufficient longitudinal data.

To address these knowledge gaps, we conducted a population-based cohort study with three principal objectives: First, to identify clinical predictors of SPM development in FL patients treated during the rituximab era (post-2000). Second, to identify distinct prognostic determinants that differentiate OS from lymphoma-specific survival (LSS) between FL patients and the subgroup developing secondary malignancies following FL diagnosis. Third, to delineate cause-specific mortality patterns distinguishing these patient subgroups. Methodologically, we employ Fine-Gray competing risk models to provide more accurate SPM risk estimation compared to traditional survival analysis. Our findings aim to inform risk-adapted surveillance protocols and refine therapeutic decision-making in FL management.

**2.Line 73 Methods: Please add study design and period. Please write the inclusion criteria. Further justification is needed for excluding SPMs diagnosed within six months of FL diagnosis. Were sensitivity analyses conducted to validate this choice? Please mention sample size calculations and sampling method.**

2.2 Patient selection

This retrospective cohort study analyzed clinical data and follow-up information from patients with follicular lymphoma from 2000 to 2020. Inclusion criteria were (1) first primary FL with histologically confirmed (ICD-O-3 histological codes 9690-9698); (2) age>14 years; (3) complete and reliable follow-up data. The exclusion criteria included (1)did not have complete and reliable clinical characteristics; (2)diagnosis established post-mortem; (3)SPMs diagnosed within a six-month interval following the initial FL diagnosis; (4)SPMs of B-cell lineage, or documented relapse of FL; (5) survival time was 0. Notably, our sensitivity analysis included all SPM cases regardless of diagnostic interval(Table S5-15).The SPM definition followed National Cancer Institute guidelines as described by Morris et al[12], requiring ≥6-month interval between FL diagnosis and subsequent malignancy. As a population-based registry study utilizing the SEER-17 dataset (2000-2020), our analysis included all eligible follicular lymphoma cases meeting predefined criteria. This exhaustive sampling approach eliminates selection bias inherent to calculated sample sizes, ensuring maximal representation of the US population covered by SEER registries (covers approximately 26.5% of the U.S. population). The aforementioned text has been incorporated into the manuscript at Line 147-166.

**3.Line 74-79: The authors mention that ethical approval was waived due to the use of de-identified data. However, they should provide more details on how they ensured the ethical use of the data, including any specific guidelines or regulations they followed.**

The data is public and patient information is anonymous, so our study does not require ethical approval or informed consent of patients. Our research follows the regulations published by the SEER database and the Declaration of Helsinki. The aforementioned text has been incorporated into the manuscript at Line 143-146.

**4.Line 80-92: The authors should provide more details on the SEER database, including its limitations and potential biases. For example, how representative is the SEER database of the general population, and are there any known biases in the data collection process? The absence of FLIPI scores, genetic markers, and detailed treatment regimens should be acknowledged more explicitly.**

This study extracted de-identified patient-level records from the SEER Research Data (17 Registries, November 2022 Submission, 2000-2020) using SEER*Stat 8.4.3. While the SEER-17 registries collectively cover approximately 26.5% of the U.S. population, their geographic distribution requires careful interpretation: The participating regions predominantly consist of selected states [California (excluding San Francisco/San Jose-Monterey/Los Angeles areas)，Connecticut, Hawaii, Iowa, Kentucky, Louisiana, New Jersey, New Mexico, Utah] and metropolitan areas (San Francisco-Oakland, Los Angeles, Seattle-Puget Sound, Atlanta, Greater Georgia, Rural Georgia, San Jose-Monterey), with intentional oversampling of specific populations (e.g., Alaska Natives). This deliberate sampling design may introduce geographic and demographic imbalances, particularly underrepresenting rural populations in non-SEER states. Three key limitations merit explicit acknowledgment. First, the absence of follicular lymphoma international prognostic index (FLIPI) scores prevents risk stratification, potentially masking survival differences among biological subgroups. Second, the absence of documented genetic biomarkers (e.g., BCL2 expression status, EZH2 mutation profiles, and chromosomal translocation patterns) hinders comprehensive assessment of genomic heterogeneity's prognostic implications. Third, while therapeutic modalities (chemotherapy, radiotherapy, and surgical resection) are recorded as binary variables, the lack of detailed treatment specifications including protocol variations, dose-intensity metrics, and temporal coordination of interventions may introduce confounding bias in comparative effectiveness research. The aforementioned text has been incorporated into the manuscript at Line 118-142.

**5.Line 107-113: The study objectives should be summarized and included as the final paragraph of the introduction.**

In response to the editor's comment, we have refined the research objectives and incorporated the updated summary into the Introduction (Lines [105-110]).

In this population-based cohort study, we aimed to (1) identify clinical risk factors for SPMs in FL patients, (2) establish prognostic predictors for both overall survival (OS) and lymphoma-specific survival (LSS) comparing FL patients with versus without SPMs, and (3) delineate differential patterns of cause-specific mortality between these two clinically distinct populations.

**6.Line 114-135: The authors mention using competing-risk regression analysis, but they should provide more details on the specific models used and how they handled competing risks. Additionally, they should clarify how they addressed potential confounding factors in their analysis.**

We employed the Fine-Gray proportional subdistribution hazards model as the primary analytical framework to quantify associations between covariates and outcomes while accounting for the interdependence of competing events. Specifically, two distinct competing-risk regression analysis were conducted: (1) For SPMs, the primary event was defined as time from FL diagnosis to SPM detection, with all-cause mortality treated as the competing event; (2) For lymphoma-specific survival, the primary event was death from FL, with non-FL-related deaths constituting the competing event. Patients were censored at last follow-up if neither event occurred. Cumulative incidence functions were estimated using the Aalen-Johansen estimator to avoid overestimation biases inherent in Kaplan-Meier methodology. To address confounding, we implemented a rigorous covariate selection protocol: first, clinically plausible variables were pre-specified through literature review; second, univariate screening with an inclusive threshold (P≤0.2) retained marginally significant factors; finally, multivariable models incorporated these predictors alongside established prognostic variables. Sensitivity analysis evaluated model robustness through subgroup stratification by SPM latency periods (≤6 vs. >6 months post-diagnosis) and methodological cross-validation using conventional Cox proportional hazards models, confirming the stability of identified risk factors across analytical paradigms(Table S5-15). The aforementioned text has been incorporated into the manuscript at Line 190-211.

**7.Line 194- 199: Table 1: The table provides a good summary of patient characteristics, but it would be helpful to include more details on the treatment modalities (e.g., specific chemotherapy regimens, radiation doses) and how they were categorized.**

We sincerely appreciate the editor's valuable insights regarding treatment modality specifications. As detailed in our Methods section, the SEER database presents inherent limitations in chemotherapy documentation, restricting classification to a binary system (Yes/No or Unknown) without details regarding specific therapeutic regimens. For radiotherapy parameters, while the original dataset contained eight categorical classifications [including: Beam radiation, Radioactive implants (brachytherapy; 1988+), Radioisotopes (1988+), Combination therapies, Radiation NOS (not otherwise specified), Recommended (unknown administration status), Refused (1988+), and None/Unknown], we intentionally consolidated these into clinically interpretable binary variables (Yes/No or Unknown) to facilitate meaningful analysis.

We fully concur with the editor's recognition of the critical importance of treatment particulars such as radiation dosimetry parameters and chemotherapy protocol specifics in outcome evaluation. However, it is essential to emphasize that these operational-level treatment details fundamentally exceed the current documentation scope of the SEER registry infrastructure. This structural limitation inherently constrains the therapeutic information available for secondary analysis, while maintaining the database's strengths in population-level observational research.

**8.Figure 1 and 2: The figures are informative, but the authors should ensure that all abbreviations are defined in the figure legends. Additionally, they should consider adding confidence intervals to the forest plot in Figure 2 to provide a more complete picture of the results**

We appreciate the reviewer's insightful suggestion. Here's the revised figure legends section with modifications to address the editor's comments:

Revised Figure Legends

Figure 1. Cumulative incidence of second primary malignancies in patients with follicular lymphoma diagnosed between 2000 and 2020.

Abbreviations: TTT, time to treatment; NHL, non-Hodgkin's lymphoma; Grade NOS, Not Otherwise Specified.

Race categories: "Others" includes American Indian/AK Native and Asian/Pacific Islander.

Marital status categories: "Others" includes divorced, separated, unmarried/domestic partner, and widowed.

Figure 2. Forest plot of multivariate competing risks regression analysis for risks of second primary malignancies in follicular lymphoma patients.

Abbreviations: NHL, non-Hodgkin's lymphoma; Grade NOS, Not Otherwise Specified.

Race categories: "Others" includes American Indian/AK Native and Asian/Pacific Islander.

Marital status categories: "Others" includes divorced, separated, unmarried/domestic partner, and widowed.

Figure 3. Survival analysis of follicular lymphoma patients based on secondary primary malignancy status.

(A) Overall survival (OS) stratified by occurrence of secondary primary malignancies (SPMs);

(B) Lymphoma-specific survival (LSS) stratified by occurrence of SPMs;

(C) Log-rank test comparison of OS between patients with and without SPMs;

(D) Log-rank test comparison of LSS between patients with and without SPMs.

These changes ensure each figure legend is self-contained, abbreviations are properly defined, and statistical completeness is maintained with the addition of confidence intervals in Figure 2.

**9.Line 278-287: The authors should provide more details on the survival analysis, including the specific time points used for the Kaplan-Meier curves and how they handled censoring in their analysis.**

In accordance with the editor's valuable recommendations, we have implemented substantive revisions to optimize the section's organizational structure and analytical precision. The key modifications include: (1) Integration of original Sections 3.4-3.5 into a cohesive Survival Analysis framework to eliminate redundancy; (2) Strategic expansion with two specialized subsections: 3.5 Prognostic factors among FL patients;3.6 Prognostic factors among FL patients developing SPMs.

We executed comprehensive survival analysis adhering to the following methodological standards:

**Survival Probabilities:**

Kaplan-Meier curves were specifically calibrated to capture survival rates at four clinically pivotal intervals (1-, 3-, 5-, and 10-year landmarks), reflecting both the indolent biological behavior of FL and standardized clinical surveillance protocols.

**Censoring Protocol:**

A rigorous right-censoring algorithm was implemented at the earliest occurrence of (1) Study closure (December 31, 2020); (2)Last confirmed alive date for lost-to-follow-up patients; (3)Mortality events unrelated to FL (exclusively for lymphoma-specific survival calculations)

**Endpoint Operationalization:**

Overall Survival (OS): Defined by all-cause mortality.

Lymphoma-Specific Survival (LSS): Defined as deaths directly attributable to FL progression or complications, ascertained through rigorous adjudication integrating two standardized SEER database parameters: "SEER Cause-Specific Death Classification" and "SEER Other Cause of Death Classification".

The aforementioned text has been incorporated into the manuscript at Line 332-342.

**10.Line 311: Discussion:**

**(1) The authors should provide a more detailed comparison of their findings with previous studies, particularly regarding the risk factors for SPMs and the impact of rituximab therapy. They should also discuss any discrepancies between their findings and those of previous studies. For example, the observation that Grade 3 FL patients had a lower risk of developing SPMs contradicts previous studies. A more thorough exploration of potential biological mechanisms is needed.**

We appreciate your insightful question, which prompted us to expand our discussion. We have incorporated this perspective into Line 451-497 of the revised manuscript and included annotated revisions in the attached document highlighting the specific modifications.

**Discussion**

Follicular lymphoma (FL) is a typically indolent lymphoma originating from germinal center B cells. Advances in treatment have significantly improved FL patient survival[13]. In the era of rituximab therapy, patients diagnosed with FL generally have a promising prognosis. Recent data from French and U.S. cohorts show 10-year OS rates of FL patients were 79.8% and 76.6%, respectively[14], although patients may still experience late effects following cancer treatment, impacting their quality of life.

Previous studies have reported that the annual percent change (APC) for FL in Taiwan from 2002 to 2019 was 3.49%, in Japan from 2001 to 2008 was 12.66%, and from 2014 to 2019 was 4.95%. In South Korea, the change was 5.72% from 2001 to 2012, and 7.93% from 2015 to 2022[15]. In contrast, the incidence rate among the Whites decreased annually by -2.1% from 2008 to 2017, dropping from 3.42 cases in 2008 to 2.74 cases per 100,000 person-years in 2017, a decrease of 20%[16]. Between 2000 and 2014 within the Polish population, no significant trend was noted in the standardized overall incidence rates of FL[17]. Consistent with the findings of the above studies, our research shows an APC of -1.6% for FL in the U.S. population from 2000 to 2020. This suggests that greater attention to FL in Asian countries is needed, along with etiological studies that focus especially on modifiable risk factors, to understand and address the factors driving the rising incidence of FL group.

Among 33,104 FL patients in our study, the median age at diagnosis was 62 years, with a balanced male-to-female ratio, matching typical FL incidence patterns[1, 18]. Consistent with prior research, male and older patients had a higher incidence of SPMs[19]. Notably, a higher proportion of patients with skin as the primary site developed SPMs compared to those without such a history. While the existing literature on this is limited, we hypothesize that this may be related to the immunosuppressive therapies, such as radiation therapy (a common treatment for primary cutaneous follicular lymphoma) and chemotherapy[20]. In our study, the SPM subtypes significantly elevated in FL patients are those thought to be linked to immune dysfunction and the use of chemotherapeutic agents, including acute myeloid leukemia (AML) and malignancies of the lung and bronchus, skin[21].

Giri et al. [22]analyzed SEER data (1992–2011; N=15,517) excluding ICD-O-3 9690/3 (FL NOS) cases due to insufficient diagnostic specificity, applying Fine-Gray competing risk models. Their analysis identified age>65 years, male sex, and prior radiotherapy(RT) as independent SPM predictors, while no significant associations observed for diagnosis year (1992–1999 vs 2000–2011), FL histological grade, or Black/White racial disparities. Our findings corroborate Giri et al.'s established SPM risk factors (age>65 years, male sex, RT) while identifying novel predictors (non-Hispanic ethnicity) and protective factors [unmarried status, Grade 3 histology, recent diagnosis (2015-2019)] through competing risk analysis. The underlying mechanisms contributing to the reduced incidence of SPMs in grade 3 FL remain underexplored in current literature, highlighting a critical knowledge gap in FL pathogenesis. A pivotal competing risk analysis demonstrated significantly higher cumulative histological transformation(HT) rates in FL3a patients compared to their FL1-2 counterparts, with 10% (5-year) and 13% (10-year) versus 2.1% and 2.9%, respectively (P < 0.001) [23]. This striking divergence suggests potential biological competition between transformation events and secondary carcinogenesis. Microenvironmental remodeling is a well-known oncogenic and procarcinogenic factor in FL. Through transcriptome analysis, Zhang[24] group explored the relationship between FL pathological grade and microenvironmental composition. From FL1-2 to FL3B, the cell cycle pathways become progressively more active, metabolic activity gradually increases, and immune pathway activity gradually decreases. These dynamic molecular shifts may create an evolutionary landscape favoring rapid clonal expansion over secondary carcinogenic processes. However, this hypothesis requires validation through multi-omics investigations characterizing the distinct genomic landscapes, tumor microenvironment interactions, and clonal evolutionary patterns distinguishing HT-prone from SPM-susceptible FL subtypes. The protective effect of recent diagnosis (2015-2019) may be attributed to advancements in FL management. First, the widespread adoption of observation without immediate therapy for asymptomatic advanced-stage FL effectively delays or avoids alkylating agents (e.g., cyclophosphamide) and radiation, both recognized as independent risk factors for therapy-related myeloid neoplasms and solid tumors. Second, for early-stage FL requiring intervention, precision radiotherapy techniques have replaced extended-field irradiation. Crucially, the therapeutic landscape has shifted toward targeted agents with lower mutagenic potential: CD20-directed monoclonal antibodies, PI3Kδ inhibitors, and CAR-T cells targeting CD19/20 now dominate relapse/refractory settings, displacing traditional regimens like CHOP that confer higher cumulative DNA damage[2]. The association between unmarried status and reduced SPM risk is intriguing. As cancer diagnosis and treatment often create prolonged psychosocial burdens for both patients and spouses, remaining single potentially circumvents these marital stressors that studies suggest marital quality is associated with health outcomes and mortality risk[25]. Notably, our findings contrast with Nie et al.'s [26]logistic regression analysis (2003-2014 cohort) that applied full covariate adjustment and demonstrated no radiotherapy-second primary cancer (SPC) association (OR = 0.92, 95%CI: 0.53–1.58) in FL patients with ≥1-year latency between malignancies, potentially reflecting methodological differences in outcome definition (SPC latency ≥1year) and analytical approaches (competing risks vs conventional regression).

**(2) The authors should expand on the clinical implications of their findings, particularly regarding the management of FL patients at risk for SPMs. They should also discuss any potential strategies for reducing the risk of SPMs in this population.**

Our findings highlight critical clinical considerations for managing FL patients at risk of SPMs. Clinicians should prioritize SPM surveillance in high-risk subgroups, including males ≥ 40years, non-Hispanic individuals, and those treated with radiotherapy (RT). RT is a powerful treatment that can effectively destroy cancer but also poses risks to healthy tissue. When radiation doses exceed the repair capacity of surrounding normal tissues, irreversible damage may occur[27]. RT can trigger immunosuppressive responses, including the polarization of macrophages towards an M2 phenotype[28], the shift of neutrophils to an N2 phenotype[29], and the accumulation of myeloid-derived suppressor cells[30]. These immune cells typically exert their immunosuppressive influence through the upregulation of PD-L1 and through various other mechanisms[31]. To mitigate risks, we propose a two-pronged approach: (1) Enhanced Surveillance: Implement annual low-dose CT screening for high-risk patients to improve early lung cancer detection and survival[32]. (2) Treatment Optimization: Employ advanced RT techniques (e.g., involved-site RT) to minimize radiation exposure to healthy tissues. Treatment decisions should weigh histologic grade (Grade 3 cases showed reduced SPM risk) and marital status (unmarried patients had lower risk). For recently diagnosed cases (2015-2019 cohort with lower SPM risk), analyze modern treatment protocols as potential models for risk reduction. Future research should clarify how immunosuppressive therapies interact with FL biology to drive SPMs, which could guide targeted interventions.

The survival analysis found that FL patients with SPMs did not show a decrease in survival within the first 91 months compared to those without SPMs, with LSS increasing only after 175 months. This could be attributed to the early identification of high-risk patients in lymphoma, targeted therapies, cellular treatments, and other interventions have demonstrated promising efficacy[33, 34].

The aforementioned text has been incorporated into the manuscript at Line 516-527.

**(3) Some hazard ratios (HRs) have confidence intervals (CIs) that approach 1.00. The clinical significance of these borderline findings should be addressed.**

Only a limited number of studies have previously investigated the risk factors associated with FL[22, 35, 36] . In concordance with prior studies, multivariate analysis reveals the Ann Arbor stage as a significant predictor of OS and LSS in FL patients, emphasizing the critical role of early diagnosis, indicating the importance of early detection[37, 38]. Moreover, the data demonstrated a decline in patient survival with older ages. This condition may be associated with immunological insufficiency and somatic decline in elderly people. Studies have revealed that individuals with B symptoms have a poor prognosis, emphasizing the need for careful monitoring of high-risk patients[39].

Notably, in the multivariate analysis of OS, several covariates demonstrated statistically significant hazard ratios (HRs) with confidence intervals (CIs) approaching 1.00. Specifically, higher income, nonmetropolitan residence, Grade 3 histology, and SPMs showed borderline effect sizes. While statistically significant, the narrow deviation of these HRs from 1.00 suggests limited clinical impact. This phenomenon may reflect either intrinsic biological effects (e.g., socioeconomic factors like income and geography exerting subtle systemic influences) or methodological constraints (e.g., inability to distinguish Grade 3A from 3B in registry data, potentially diluting prognostic discrimination). Notably, the delayed OS impact of SPMs at 91 months in Kaplan-Meier analysis further implies temporal effect modification. Similar patterns emerged in LSS analysis, where non-Hispanic ethnicity, higher income, and Grade 3 demonstrated marginal associations. These findings warrant cautious interpretation: although meeting statistical significance thresholds, the clinical relevance of such small-magnitude effects requires validation through risk-stratified interventions or mechanistic studies. Future studies should incorporate detailed histopathological subclassification and longitudinal socioeconomic metrics to improve prognostic precision.

The aforementioned text has been incorporated into the manuscript at Line 544-562.

(4) The potential impact of changing treatment strategies over time on survival outcomes should be further discussed.

Dear Editor,

Through rigorous methodological refinements in our extended analysis, we observed no statistically significant association between surgical intervention and clinical outcomes in patients with SPMs. This conclusion supersedes our prior preliminary findings due to enhanced adjustment for treatment-era covariates and more stringent data curation protocols. The complete analytical dataset, including reproducibility scripts, has been systematically cataloged in Attachment TableS16-17 for independent verification.

Our survival analysis revealed distinct therapeutic impacts on patient outcomes. Initial chemotherapy emerged as a significant risk factor for diminished LSS, whereas it showed no measurable impact on OS. In contrast, both radiotherapy and surgical intervention demonstrated significant associations with reduced all-cause mortality in FL patients. Notably, among patients developing SPMs, initial radiotherapy maintained protective effects for both OS and LSS. These findings emphasize the necessity for personalized therapeutic decision-making that carefully weighs these differential survival outcomes. The consistency of these findings with current clinical guidelines reinforces the validity of our study conclusions[2].

The aforementioned text has been incorporated into the manuscript at Line 563-573.

**(5) The authors acknowledge some limitations of their study, but they should provide a more detailed discussion of these limitations and how they might have affected the results. For example, they should discuss the potential impact of missing data, selection bias, and the lack of information on specific treatment regimens.**

Our study has limitations: First, the retrospective SEER-based design introduces potential selection bias through uneven population representation, possibly compromising the generalizability of survival outcomes (particularly OS/LSS) beyond U.S. healthcare contexts. Second, absent granular treatment data (radiation fields, chemotherapy regimens/schedules, surgical approaches) and FLIPI scores prevents adjustment for critical prognostic confounders, potentially distorting therapeutic effect estimates. The inability to distinguish histological grades 3A vs 3B—subtypes with divergent clinical behaviors—may have introduced misclassification bias, obscuring true survival differences. While we conducted rigorous multivariable adjustments, these data gaps likely introduced residual confounding. Future studies should use prospective data to validate and expand these findings.

The aforementioned text has been incorporated into the manuscript at Line 574-586.

(6) The conclusion is concise and summarizes the main findings of the study. However, the authors should consider adding a brief discussion of the broader implications of their findings for future research and clinical practice.

In conclusion, using the largest FL cohort to date, our study meticulously describes the epidemiological characteristics of FL. After excluding the potential for transformation to more aggressive subtypes and the competing risk of death, the study identifies the risk factors contributing to the development of SPMs in FL patients and delves into the causes of death among patients. Additionally, we analyzed the risk factors affecting the survival rates of FL patients, as well as those patients with SPMs. These results carry translational significance by informing risk-adapted surveillance strategies - particularly the need for enhanced malignancy screening protocols in high-risk subgroups and personalized comorbidity management in elderly populations.  Future investigations should focus on validating these predictive models across diverse healthcare settings, elucidating the molecular mechanisms underlying SPM pathogenesis in FL, and evaluating the clinical utility of targeted screening interventions through prospective trials.

The aforementioned text has been incorporated into the manuscript at Line 595-602.

Review Comments to the Author

Reviewer #1: There are several comments that the authors need to improvised in order for the manuscript to more understandable. For example:

**11.ABSTRACT**

**The authors should address the conclusion in the abstract as it can summarize the author’s overall findings and the future plan of the study.**

We appreciate the reviewer's insightful comment. In response to the suggestion regarding conclusion presentation, we have substantially revised the abstract section.

Conclusion:

In this study, we conducted a large, population-based analysis across the United States to identify risk factors for the development of SPMs and to delineate prognostic indicators for FL patients in the context of rituximab therapy, along with the clinical characteristics, risk factors, and prognostic features associated with SPMs. These findings have translational implications for risk-adapted surveillance. Future studies should validate predictive models across diverse healthcare settings, elucidate molecular mechanisms of SPM pathogenesis in FL, and evaluate targeted screening interventions through prospective trials.

The aforementioned text has been incorporated into the manuscript at Line 53-61.

**12.INTRODUCTION**

**It would be more beneficial if the authors could elaborate more in regards to the mortality rate of FL in recent years, the global prevalence of FL and how it sparks your interest to conduct this retrospective study.**

We sincerely appreciate the reviewer's insightful comments. In accordance with the suggestions, we have substantially revised the Introduction section, with key modifications highlighted in red to explicitly address the editorial concerns and enhance the clarity of our responses. These revisions have been consistently incorporated throughout the manuscript to ensure uniformity with the updated content.

Follicular lymphoma (FL), originating from the germinal center B cells in lymph nodes or lymphoid tissue, is one of the most common indolent forms of non-Hodgkin's lymphoma (NHL). In Western countries, FL accounts for about 20-35% of NHL cases[1, 2], with an age-standardized incidence of 2–4 per 100,000 person-years[1]. The therapeutic landscape for FL has been revolutionized by anti-CD20 monoclonal antibodies, particularly rituximab-based immunochemotherapy, which has elevated 10-year overall survival (OS) rates beyond 80%[2]. While these advancements have substantially improved survival outcomes, emerging survivorship challenges including long-term treatment toxicities and second primary malignancies (SPMs) demand increased clinical attention. Notably, contemporary studies indicate that SPM incidence remains unaffected by the incorporation of rituximab into therapeutic regimens[3-6], emphasizing the critical need for optimized surveillance strategies and preventive measures in the current era of rituximab-dominated therapies.

The aforementioned text has been incorporated into the manuscript at Line 66-78.

**13.METHODS**

**Is it possible for the authors to mention the specific date of the data were taken, as I can see the year 2000 to 2020 is too general.**

Thank you for raising this important point regarding the data timeframe. In response to the comment, we have clarified the exact time span as January 2000 to December 2020. This specific date range has been incorporated in Lines 149-150 of the revised manuscript to precisely define our data collection period.

**14.RESULTS**

**As the years of FL incidence were reported at the first 2 paragraphs of the result section (Line 138-148), I would advise the authors to summarized it as well in a simple line graph for the readers to understand in more easily.**

We sincerely appreciate the constructive feedback on optimizing data visualization. In response, we have added a refined line graph (Figure S1A) in the Supplementary Materials section to illustrate FL incidence trends between 2000 and 2020. Furthermore, gender-stratified (Figure S1B) and age group-stratified (Figure S1C) analyses were performed to identify demographic-specific epidemiological patterns.

**15.DISCUSSION**

**Discussion part were presented quite well and easy to be understood. In addition to this, I would suggest that the author to add 1-2 more sentences to explain the strength of the current study.**

Thank you for your positive feedback regarding the clarity of the Discussion section. In response to your valuable suggestion, we have incorporated two additional sentences in the Conclusion section (Line 587-602) to explicitly articulate the methodological strengths and unique contributions of this study. This enhancement aligns with your recommendation and serves to better emphasize the scientific value of our research outcomes for readers' reference.

**16.CONCLUSION**

**I would suggest that the conclusion section to be separated from the discussion section.**

We sincerely appreciate this insightful suggestion. In accordance with your recommendation, we have restructured the manuscript by creating a standalone Conclusions section (now Section 5), distinct from the Discussion (Section 4). This modification enhances organizational clarity.

**17.Reviewer #2: Just a small grammatical error correction needed. It is also suggested that the author updated the list of references (at least in the last five years).**

We gratefully acknowledge your valuable suggestions. The reference section has been systematically updated, and all grammatical errors have been corrected.

1.	Carbone A, Roulland S, Gloghini A, Younes A, von Keudell G, López-Guillermo A, Fitzgibbon J: **Follicular lymphoma**. *Nature reviews Disease primers* 2019, **5**(1):83.

2.	Jacobsen E: **Follicular lymphoma: 2023 update on diagnosis and management**. *American journal of hematology* 2022, **97**(12):1638-1651.

3.	Bachy E, Houot R, Morschhauser F, Sonet A, Brice P, Belhadj K, Cartron G, Audhuy B, Fermé C, Feugier P *et al*: **Long-term follow up of the FL2000 study comparing CHVP-interferon to CHVP-interferon plus rituximab in follicular lymphoma**. *Haematologica* 2013, **98**(7):1107-1114.

4.	Moccia AA, Taverna C, Schär S, Vanazzi A, Rondeau S, Hitz F, Mingrone W, Pabst T, Cevreska L, Del Giglio A *et al*: **Prolonged rituximab maintenance in follicular lymphoma patients: long-term results of the SAKK 35/03 randomized trial**. *Blood advances* 2020, **4**(23):5951-5957.

5.	Prusila REI, Sorigue M, Jauhiainen J, Mercadal S, Postila A, Salmi P, Tanhua T, Tikkanen S, Kakko S, Kuitunen H *et al*: **Risk of secondary haematological malignancies in patients with follicular lymphoma: an analysis of 1028 patients treated in the rituximab era**. *British journal of haematology* 2019, **187**(3):364-371.

6.	Fleury I, Chevret S, Pfreundschuh M, Salles G, Coiffier B, van Oers MH, Gisselbrecht C, Zucca E, Herold M, Ghielmini M *et al*: **Rituximab and risk of second primary malignancies in patients with non-Hodgkin lymphoma: a systematic review and meta-analysis**. *Annals of oncology : official journal of the European Society for Medical Oncology* 2016, **27**(3):390-397.

7.	Watanabe T, Tobinai K, Wakabayashi M, Morishima Y, Kobayashi H, Kinoshita T, Suzuki T, Yamaguchi M, Ando K, Ogura M *et al*: **Outcomes after R-CHOP in patients with newly diagnosed advanced follicular lymphoma: a 10-year follow-up analysis of the JCOG0203 trial**. *The Lancet Haematology* 2018, **5**(11):e520-e531.

8.	Hiddemann W, Barbui AM, Canales MA, Cannell PK, Collins GP, Dürig J, Forstpointner R, Herold M, Hertzberg M, Klanova M *et al*: **Immunochemotherapy With Obinutuzumab or Rituximab for Previously Untreated Follicular Lymphoma in the GALLIUM Study: Influence of Chemotherapy on Efficacy and Safety**. *Journal of clinical oncology : official journal of the American Society of Clinical Oncology* 2018, **36**(23):2395-2404.

9.	Guadagnolo BA, Li S, Neuberg D, Ng A, Hua L, Silver B, Stevenson MA, Mauch P: **Long-term outcome and mortality trends in early-stage, Grade 1-2 follicular lymphoma treated with radiation therapy**. *International journal of radiation oncology, biology, physics* 2006, **64**(3):928-934.

10.	Barzenje DA, Cvancarova Småstuen M, Liestøl K, Fosså A, Delabie J, Kolstad A, Holte H: **Radiotherapy Compared to Other Strategies in the Treatment of Stage I/II Follicular Lymphoma: A Study of 404 Patients with a Median Follow-Up of 15 Years**. *PloS one* 2015, **10**(7):e0131158.

11.	Kimby E, Schär S, Pirosa MC, Vanazzi A, Mey UM, Rauch D, Wahlin BE, Hitz F, Hernberg M, Johansson AS *et al*: **Six-month rituximab-lenalidomide regimen in advanced untreated follicular lymphoma: SAKK 35/10 trial 10-year update**. *Blood advances* 2025, **9**(7):1712-1719.

12.	Morris LG, Sikora AG, Patel SG, Hayes RB, Ganly I: **Second primary cancers after an index head and neck cancer: subsite-specific trends in the era of human papillomavirus-associated oropharyngeal cancer**. *Journal of clinical oncology : official journal of the American Society of Clinical Oncology* 2011, **29**(6):739-746.

13.	Zinzani PL, Muñoz J, Trotman J: **Current and future therapies for follicular lymphoma**. *Experimental hematology & oncology* 2024, **13**(1):87.

14.	Sarkozy C, Maurer MJ, Link BK, Ghesquieres H, Nicolas E, Thompson CA, Traverse-Glehen A, Feldman AL, Allmer C, Slager SL *et al*: **Cause of Death in Follicular Lymphoma in the First Decade of the Rituximab Era: A Pooled Analysis of French and US Cohorts**. *Journal of clinical oncology : official journal of the American Society of Clinical Oncology* 2019, **37**(2):144-152.

15.	Chiu LC, Lin CW, Li HJ, Chen JH, Chuang FC, Lin SF, Chang Y, Su YC: **Incidence Trend of Follicular Lymphoma in Taiwan Compared to Japan and Korea, 2001-2019**. *Journal of clinical medicine* 2023, **12**(4).

16.	Su YC, Chiu BC, Li HJ, Yang WC, Chen TY, Yeh SP, Wang MC, Huang WT, Lee MY, Lin SF: **Investigation of the incidence trend of follicular lymphoma from 2008 to 2017 in Taiwan and the United States using population-based data**. *PloS one* 2022, **17**(3):e0265543.

17.	Szumera-Ciećkiewicz A, Wojciechowska U, Didkowska J, Poleszczuk J, Rymkiewicz G, Paszkiewicz-Kozik E, Sokół K, Prochorec-Sobieszek M, Walewski J: **Population-based epidemiological data of follicular lymphoma in Poland: 15 years of observation**. *Scientific reports* 2020, **10**(1):14610.

18.	Dada R: **Diagnosis and management of follicular lymphoma: A comprehensive review**. *European journal of haematology* 2019, **103**(3):152-163.

19.	Liu Y, Chu Y, Liu J, Ge X, Ding M, Li P, Liu F, Zhou X, Wang X: **Incidence and mortality of second primary malignancies after lymphoma: a population-based analysis**. *Annals of medicine* 2023, **55**(2):2282652.

20.	Nikolaou V, Koumprentziotis IA, Papadavid E, Patsatsi A, Diavati S, Tsimpidakis A, Kruger-Krasagakis S, Doxastaki A, Marinos L, Kaliampou S *et al*: **Clinical features, treatment options and outcomes in primary cutaneous B-cell lymphomas: a real-world, multicenter, retrospective study**. *International journal of dermatology* 2024.

21.	Dinnessen MAW, Visser O, Tonino SH, Posthuma EFM, Blijlevens NMA, Kersten MJ, Lugtenburg PJ, Dinmohamed AG: **Risk of second primary malignancies in patients with follicular lymphoma: a population-based study in the Netherlands, 1989-2018**. *Blood cancer journal* 2021, **11**(11):179.

22.	Giri S, Bhatt VR, Verma V, Pathak R, Bociek RG, Vose JM, Armitage JO: **Risk of Second Primary Malignancies in Patients With Follicular Lymphoma: A United States Population-based Study**. *Clinical lymphoma, myeloma & leukemia* 2017, **17**(9):569-574.

23.	Zha J, Chen Q, Ye J, Yu H, Yi S, Zheng Z, Xu W, Li Z, Ping L, He X *et al*: **Differences in clinical characteristics and outcomes between patients with grade 3a and grades 1-2 follicular lymphoma: a real-world multicenter study**. *Biomarker research* 2023, **11**(1):16.

24.	Sun C, Li W, Yu J, Zhang T, Gong W, Liu H, Gao F, Song Z, Li L, Qiu L *et al*: **Molecular landscape of distinct follicular lymphoma histologic grades: insights from genomic and transcriptome analyses**. *Leukemia* 2025.

25.	An H, Chen C, Du R, Cheng C, Wang P, Dong S: **Self-efficacy, psychological distress, and marital quality in young and middle-aged couples facing lymphoma: The mediating effect of dyadic coping**. *Psycho-oncology* 2021, **30**(9):1492-1501.

26.	Li L, He Z, Zhou Q, Nie B: **The association between radiotherapy and the prognosis of follicular lymphoma patients with different characteristics in the rituximab era: a cohort study based on Surveillance, Epidemiology and End Results**. *Annals of hematology* 2025, **104**(1):691-700.

27.	Guo S, Yao Y, Tang Y, Xin Z, Wu D, Ni C, Huang J, Wei Q, Zhang T: **Radiation-induced tumor immune microenvironments and potential targets for combination therapy**. *Signal transduction and targeted therapy* 2023, **8**(1):205.

28.	Genard G, Lucas S, Michiels C: **Reprogramming of Tumor-Associated Macrophages with Anticancer Therapies: Radiotherapy versus Chemo- and Immunotherapies**. *Frontiers in immunology* 2017, **8**:828.

29.	Boivin G, Ancey PB, Vuillefroy de Silly R, Kalambaden P, Contat C, Petit B, Ollivier J, Bourhis J, Meylan E, Vozenin MC: **Anti-Ly6G binding and trafficking mediate positive neutrophil selection to unleash the anti-tumor efficacy of radiation therapy**. *Oncoimmunology* 2021, **10**(1):1876597.

30.	Liang H, Deng L, Hou Y, Meng X, Huang X, Rao E, Zheng W, Mauceri H, Mack M, Xu M *et al*: **Host STING-dependent MDSC mobilization drives extrinsic radiation resistance**. *Nature communications* 2017, **8**(1):1736.

31.	Wang NH, Lei Z, Yang HN, Tang Z, Yang MQ, Wang Y, Sui JD, Wu YZ: **Radiation-induced PD-L1 expression in tumor and its microenvironment facilitates cancer-immune escape: a narrative review**. *Annals of translational medicine* 2022, **10**(24):1406.

32.	Oudkerk M, Liu S, Heuvelmans MA, Walter JE, Field JK: **Lung cancer LDCT screening and mortality reduction - evidence, pitfalls and future perspectives**. *Nature reviews Clinical oncology* 2021, **18**(3):135-151.

33.	Sant M, Vener C, Lillini R, Rossi S, Bonfarnuzzo S, Marcos-Gragera R, Maynadié M, Innos K, Paapsi K, Visser O *et al*: **Long-term survival for lymphoid neoplasms and national health expenditure (EUROCARE-6): a retrospective, population-based study**. *The Lancet Oncology* 2024, **25**(6):731-743.

34.	Batlevi CL, Sha F, Alperovich A, Ni A, Smith K, Ying Z, Soumerai JD, Caron PC, Falchi L, Hamilton A *et al*: **Follicular lymphoma in the modern era: survival, treatment outcomes, and identification of high-risk subgroups**. *Blood cancer journal* 2020, **10**(7):74.

35.	Morton LM, Curtis RE, Linet MS, Bluhm EC, Tucker MA, Caporaso N, Ries LA, Fraumeni JF, Jr.: **Second malignancy risks after non-Hodgkin's lymphoma and chronic lymphocytic leukemia: differences by lymphoma subtype**. *Journal of clinical oncology : official journal of the American Society of Clinical Oncology* 2010, **28**(33):4935-4944.

36.	Zheng W, Wang S, Liang Y, Ning H: **Is radiotherapy still the optimal initial choice for patients with early-stage low-grade follicular lymphoma in the modern era? A population-based study**. *Annals of hematology* 2024, **103**(11):4589-4598.

37.	Guevara DL, Bernard S, Manhood S, Melani S, Yerovi F, Rodríguez M: **[Prognostic value of interim PET/CT in non-hodgkin lymphoma]**. *Revista medica de Chile* 2020, **148**(11):1558-1567.

38.	Li X, Yang Z, Li J, Wang G, Sun A, Wang Y, Zhang W, Liu Y, Lei H: **The development of a prediction model based on random survival forest for the prognosis of non- Hodgkin lymphoma: A prospective cohort study in China**. *Heliyon* 2024, **10**(12):e32788.

39.	Iqbal M, Jiang L, Li KD, Moustafa MA, Kimbrough EO, Ansell SM, Tun HW: **Poly-lymphomatous Syndrome With Concurrent or Sequential Hodgkin and Non-Hodgkin lymphoma**. *Clinical lymphoma, myeloma & leukemia* 2023, **23**(2):138-144.
